# Effects and Moderators of Acute Aerobic Exercise on Subsequent Interference Control: A Systematic Review and Meta-Analysis

**DOI:** 10.3389/fpsyg.2019.02616

**Published:** 2019-11-21

**Authors:** Max Oberste, Florian Javelle, Sophia Sharma, Niklas Joisten, David Walzik, Wilhelm Bloch, Philipp Zimmer

**Affiliations:** ^1^Department of Molecular and Cellular Sport Medicine, Institute of Cardiovascular Research and Sports Medicine, German Sport University Cologne, Cologne, Germany; ^2^Medical Faculty, University of Cologne, Cologne, Germany; ^3^Department of Exercise and Health, Institute of Sports Science, Leibniz University Hannover, Hanover, Germany

**Keywords:** exercise, physical activity, cognition, interference control, Stroop, Flanker

## Abstract

**Background:** Acute aerobic exercise leads to positive physiological adaptations within the central nervous system. These findings inspired research on potential cognitive benefits following acute aerobic exercise. The effects of acute aerobic exercise on subsequent cognitive performance, by far, have been the most researched for interference control, a subcomponent of executive function. The results of primary studies on the effects of acute aerobic exercise on subsequent interference control performance are inconsistent. Therefore, we used meta-analytic methods to pool available effect sizes, and to identify covariates that determine the magnitude of exercise-induced interference control benefits.

**Methods:** Medline, PsycINFO, and SPORTDiscus were searched for eligible records. Hedges' g corrected standardized mean difference values (SMDs) were used for analyses. Random-effects weights were used to pool effect sizes. Moderator analyses were conducted using meta-regressions and subgroups analyses. Covariates that were here tested for moderation included parameters of the applied exercise regimen (exercise intensity and exercise duration), characteristics of examined participants (age and fitness), and methodological features of existing research (type of control group, familiarization with test procedure, type of test variable, delay between exercise cessation, and testing).

**Results:** Fifty studies, with data from 2,366 participants, were included in qualitative and quantitative synthesis. A small, significant beneficial effect of acute aerobic exercise on time-dependent measures of interference control was revealed (k = 49, Hedges' g = −0.26, 95%CI: −34 to −0.18). Effect sizes from time-dependent measures of interference control varied widely and heterogeneity reached statistical significance (*T*^2^ = 0.0557, *I*^2^ = 28.8%). Moderator analyses revealed that higher exercise intensities (vigorous intensity and high-intensity interval training), also participants at younger or older age, and participants who are familiar with the testing procedure prior to the experiment, benefitted most from acute aerobic exercise. However, noticeable heterogeneity remained unexplained within specific subgroups (high-intensity interval training, preadolescent children, and active and supervised control group).

**Conclusion:** Acute aerobic exercise improves subsequent interference control performance. However, the covariates exercise intensity, participants' age, and familiarization with testing procedure determine the magnitude of that effect. Methodological features were not found to influence the magnitude of effects. This dismisses some doubts that exercise induced benefits for interference control performance are scientific artifacts. The fact that large heterogeneity remained unexplained in some subgroups indicates the need for further research on covariates within these subgroups. It should be noted that effect sizes for all analyses were small.

## Introduction

A large volume of studies shows physiological adaptations to acute aerobic exercise within the central nervous system. Acute aerobic exercise increases prefrontal oxygenation (Endo et al., 2013) and cortical activation (Yanagisawa et al., 2010). It is associated with an increase of circulating neurotrophins (Schmolesky et al., 2013), catecholamines (Chmura et al., 1994), and hypothalamic-pituitary-adrenal axis hormones (Wideman et al., 2002). Acute aerobic exercise improves the metabolic status of cerebral neurons (Dalsgaard et al., 2004). The physiological changes resulting from acute aerobic exercise have raised questions about its effects on central nervous system functions. A large and growing body of literature has investigated the effects of acute aerobic exercise on subsequent cognitive performances. In the recent 15–20 years, research has increasingly focused on higher cognitive performances. Interference control is, by far, the most researched higher cognitive domain in the acute exercise-cognition paradigm (Pontifex et al., 2019). It is a subcomponent of inhibition, which is a core executive function (Diamond, 2013). Interference control was defined as the capacity to prevent disruption by competing stimuli, in order to maintain goal-directed interactions with the environment (Nigg, 2000; Friedman and Miyake, 2004).

Study results on the effects of acute aerobic exercise on subsequent interference control performance are inconsistent. Several studies reported beneficial effects (Kamijo et al., 2007; Endo et al., 2013; Chen et al., 2014). At the same time, there is a group of large and well-powered trials that failed to replicate beneficial effects (Gothe et al., 2013; De Marco et al., 2014; Weng et al., 2015; Oberste et al., 2016). One potential explanation for the inconsistency of results are differences between studies concerning the applied exercise regimen. The physiological adaptations to acute aerobic exercise within the central nervous system are strongly influenced by intensity and duration of the applied aerobic exercise session (Knaepen et al., 2010; McMorris, 2015; Anderson et al., 2019). This seems to be particularly the case for the exercise-induced changes in the dorsolateral prefrontal cortex (Yanagisawa et al., 2010; Kao et al., 2017; Ligeza et al., 2018). A region that is consistently associated with interference control performances (Leung et al., 2000). Therefore, we conducted a moderator analysis that investigated the influence of intensity and duration of an acute bout of aerobic exercise on its effect on subsequent interference control performance.

Differences between studies concerning the characteristics of examined participants might also explain the inconsistency of results. Existing studies investigated the effects of acute aerobic exercise on subsequent interference control in a wide range of age groups. However, the development of interference control performance across the life span resembles an inverted-U shaped curve. During childhood, adolescence, and older adulthood, interference control performance is noticeably lower (Brydges et al., 2013; Pettigrew and Martin, 2014). During young adulthood, individuals typically reach their peak performance (Zelazo et al., 2004; Lustig and Jantz, 2015). It is questionable to what extent healthy young adults, who are at the peak of their cognitive capacities, have potential left for exercise induced cognitive improvements (Stillman et al., 2016; Whitley et al., 2016). On the contrary, preadolescent children, adolescents, and older adults might benefit substantially from acute aerobic exercise, as their baseline performance is lower. The same applies for participants' aerobic fitness. Cross-sectional research showed that higher aerobic fitness is associated with better baseline interference control performances than lower aerobic fitness (Buck et al., 2008; Huang et al., 2015). There is evidence, which shows that individuals with lower baseline cognitive performance benefit most from acute aerobic exercise (Sibley and Beilock, 2007; Drollette et al., 2014; Dimitrova et al., 2017). Therefore, participants' age and aerobic fitness was included in the moderator analysis.

Finally, methodological features of existing research might explain the inconsistency of results. Doubts were expressed that beneficial effects of acute aerobic exercise on subsequent cognitive performances might represent scientific artifacts due to methodological shortcomings (Szabo, 2013; Oberste et al., 2017). Several existing studies used control group treatment that exhibits far less psychosocial stimulation compared to the exercise treatment. While the participants in the experimental group completed a supervised exercise session, the participants in the control group sat alone and physically inactive in a waiting room (Sibley et al., 2006; Byun et al., 2014; Chang Y. K. et al., 2015; Chang et al., 2017). Differences between experimental and control group in any factor, but the independent variable, are a threat to a trial's internal validity (Srinagesh, 2006). It was argued that supervised exercise treatments raise higher expectations in the participants for cognitive benefits than inactive, non-supervised control group treatments (Szabo, 2013; Oberste et al., 2017). Expectations play a key role in the placebo effect (Brown, 2015). Therefore, it was discussed if reported improvement in interference control performance might not be a result of the preceding exercise but rather reflect expectation-driven placebo effects (Szabo, 2013; Oberste et al., 2016, 2017).

Another methodological feature that potentially explains variation of reported acute aerobic exercise induced effects on subsequent interference control is whether or not participants had been familiarized with the cognitive testing procedure prior to the actual experiment. Participants should be familiarized with a cognitive test procedure before the actual experiment starts in order to minimize practice effects (e.g., improvement of handling and operation of computerized testing; Theisen et al., 1998). Practice effects threaten a trial's internal validity (Srinagesh, 2006). However, several studies did not report familiarization with the cognitive testing procedures before the actual experiment (Sibley et al., 2006; Byun et al., 2014; Lowe et al., 2014; Chang Y. K. et al., 2015; Chang et al., 2017). If participants were not given the chance to practice the test procedure before the actual experiment begins, it remains unclear whether a beneficial effect of acute aerobic exercise reflects an improvement of interference control or a facilitation of familiarization with the test procedure.

Another potentially moderating factor associated with methodological quality of studies, which might explain inconsistency of results, is the type of variable that studies used to measure interference control performance. Tests that measure interference control, like e.g., the Stroop and Flanker task, consist of a congruent (or basic) and an incongruent (or interference) condition. In the congruent condition, participants are instructed to react as quickly and correctly as possible to target stimuli that are presented consecutively. Each target stimulus is presented within an array of neutral or identical stimuli. Performance in the congruent condition, therefore, does not depend on interference control but on basic information processing. In the incongruent condition, participants are also instructed to react as quickly and correctly as possible to consecutively presented target stimuli. Here, however, each presented target stimulus is surrounded by distracting stimuli or stimuli that induce an opposing automatic response to the target behavior. Resolving the interference between target and distracting stimuli in the incongruent condition requires not only interference control processes, but also basic information processing. The result is a decrease of a participant's performance in the incongruent condition compared to the congruent condition. A valid measure of interference control performance can be obtained, if the portion of incongruent condition-performance related to interference control is separated from the portion of incongruent condition-performance related to basic information processing. This can approximately be achieved by comparing performance in the congruent and in the incongruent condition for each participant (Stroop, 1935; Eriksen and Eriksen, 1974; Golden, 1978). Nevertheless, several studies that examine the effect of acute aerobic exercise on interference control performance, use only participants' performance in the incongruent condition as dependent measure (Chang Y. K. et al., 2015; Weng et al., 2015). This approach threatens the test's construct validity. It cannot be ruled out that an observed beneficial effect of acute aerobic exercise is due to facilitation of basic information processing, rather than an improvement of interference control.

In this systematic review and meta-analysis, we give an overview on the research of the effects of acute aerobic exercise on subsequent interference control performance. Moreover, we provide a comprehensive moderator analysis that investigates the influence of parameters of exercise regimen, characteristics of examined participants, and methodological features of existing research on acute aerobic exercise induced interference control facilitation.

## Methods

The implementation of this systematic review and meta-analysis followed the methods described in the Cochrane Handbook of Systematic Reviews (Higgins and Green, 2011). Reporting is in accordance with the “Preferred Reporting Items for Systematic Reviews and Meta-Analyses” (PRISMA) guidelines (Moher et al., 2009). We provide the PRISMA checklist in the Supplementary Material to this article. The key features of the review protocol were prospectively registered on PROSPERO (registration number: CRD42019124346). However, the inclusion criteria was extended during the course of the literature search to this review. Initially, it was planned only to include studies in young healthy adults. Different to what was initially expected, however, a sufficient number of studies in preadolescent children, adolescents, and older adults was found (compare registration update).

### Trial Eligibility Criteria

Eligible studies for this review were published peer-reviewed in English language. No limits were applied concerning the year of publication. We defined the eligibility criteria for this review based on the PICOS approach (Liberati et al., 2009).

#### Population

We included studies that investigated healthy individuals. Studies were excluded if they examined animals or ill individuals.

#### Intervention

Aerobic exercise was understood as defined by the American college of Sports Medicine: aerobic exercise is ‘[…]any activity that uses large muscle groups, can be maintained continuously, and is rhythmic in nature (Arena, 2013). We included studies that applied a single aerobic exercise session of up to 60 min duration. Studies were excluded if they applied acute resistance/strength exercise or repeated exercise sessions.

#### Comparison

Trials were eligible for this review if they compared the effects of acute aerobic exercise with a control condition. However, studies were excluded if the treatment in the control condition exceeded the threshold for light intensity exercise as defined by Norton et al. (2010) (see detailed explanation below in section Moderator Analysis).

#### Outcome

We included studies that captured interference control performance and administered the tests immediately to 60 min after exercise cessation. Studies were excluded if they conducted cognitive testing only during exercise or later than 60 min after exercise cessation.

### Search Strategy

The electronic databases Medline, PsycINFO, and SPORTDiscus were searched via EBSCO host (last updated on 5th of September, 2019). Search terms were selected to capture a broad range of studies that could then be evaluated more extensively. The following search algorithm was used (with filter applied for English language and Population Type: Human):

(exercise^*^ [Title] OR sport^*^ [Title] OR “physical activity” [Title] OR “physical exertion” [Title] OR running [Title] OR jogging [Title] OR walking [Title] OR bicycling [Title]) AND (cogniti^*^ [Title] OR “executive function^*^”* [Title] OR inhibition [Title] OR “interference control” [Title] OR Flanker [Title] OR Stroop [Title] OR Simon [Title])*.

For additional eligible records, we searched recent reviews from the field of acute exercise induced cognitive benefits (Chang et al., 2012; Verburgh et al., 2014; Ludyga et al., 2016), as well as the references of all included articles. Two members of the review team (MO and SS) independently conducted the literature search. Any disagreements were resolved by discussion of the full text.

### Outcome Measures and Data Extraction

Information on sample characteristics, intervention details, details of control group, experimental procedure, cognitive testing, and outcome data at post-intervention were extracted. If adequate data for meta-analysis was not reported, the corresponding author was contacted and data was requested. If relevant data was graphed, data was derived from figures using the Web Plot Digitizer (Tsafnat et al., 2014).

We extracted time-dependent and accuracy measures of interference control from studies if available. Reported comparisons between participants' results in congruent and in incongruent test condition (also known as Flanker effect or Stroop effect) as variable representing interference control performance were preferably extracted for analyses. For studies reporting only participants' performance in incongruent test condition, data for this variable was extracted for meta-analysis.

In cases of multiple treatment arms, which were eligible for inclusion and did not differ in terms of the here investigated moderator variables (see section Moderator Analyses below), data was combined following suggestions by the Cochrane Handbook of Systematic Reviews (Higgins and Deeks, 2011). If participants were repeatedly tested, only the first measurement time point following exercise cessation was used for analysis, in order to avoid unit-of-analysis error (Deeks, 2017). Two members of the review team (MO and SS) independently conducted data extraction. Any inconsistencies between the two were resolved by discussion and checking the full text.

### Risk of Bias Assessment

The risk of bias within included studies was assessed using the Physiotherapy Evidence Database (PEDro) scale. Namely, the PEDro scale consists of 11 items. However, the items “blinding of subjects” and “blinding of therapists” cannot be fulfilled in studies that investigate the effects of exercise. This is due to the fact that subjects actively engage in exercise treatments, and therapists use their practical skills to supervise and adjust the treatment (De Morton, 2009). Therefore, these two items were disregarded and only the following nine items were rated: (1) eligibility criteria, (2) random allocation, (3) concealed allocation, (4) baseline comparability, (5) blinding of assessors, (6) completeness of follow-up, (7) intention-to-treat-analysis, (8) between group statistical comparisons, (9) point estimates and variability. An item was rated as “low risk of bias” if the full text clearly stated that the item requirements were fulfilled. If the full text did not clearly describe that the item requirements were met, it was rated as “high risk of bias.” Two members of the review team (MO and SS) rated the risk of bias within included studies using the PEDro scale. The initial level of agreement between raters was excellent [intraclass correlation coefficient (ICC) = 0.96]. Any inconsistencies between raters were resolved in consultation with a third author (FJ).

### Moderator Analysis

Potential moderation of (1) characteristics of the applied acute aerobic exercise session, (2) characteristics of the examined participants, and (3) methodological features of the existing research was investigated:

(1) Characteristics of the applied acute aerobic exercise session

*Intensity of acute aerobic exercise session:* Effect sizes were divided into subgroups depending on the intensity of the applied acute aerobic exercise session. Light, moderate, and vigorous intensity, as well as high-intensity interval training (HIIT) were distinguished. Light, moderate, and vigorous intensity were operationalized through the guidelines by Norton et al. (2010). An acute aerobic exercise session was defined as light, if the article reported a rating of perceived exertion between 8 and 10 on the Borg scale, a heart rate between 40 and 55% of maximum heart rate, a heart rate between 20 and 40% of heart rate reserve, or an oxygen uptake between 20 and 40% of maximum oxygen uptake. An acute aerobic exercise session was defined as moderate, if the article reported a rating of perceived exertion between 11 and 13 on the Borg Scale, a heart rate between 56 and 70% of maximum heart rate, a heart rate between 41 and 60% of heart rate reserve, or an oxygen uptake between 41 and 60% of maximum oxygen uptake. An acute aerobic exercise session was defined as vigorous, if the article reported a rating of perceived exertion between 14 and 16 on the Borg Scale, a heart rate between 71 and 90% of maximum heart rate, a heart rate between 61 and 85% of heart rate reserve, or an oxygen uptake between 60 and 85% of maximum oxygen uptake. High-intensity interval training was defined according to the definition by Hurst and colleagues as an acute aerobic exercise, which consists of repeated high-intensity bouts interspersed by short active recovery periods (Hurst et al., 2019). We considered intervals within HIIT as high intensity, if they fulfilled at least the above-described criterions for vigorous intensity operationalized through the guidelines by Norton et al. (2010). Only studies that controlled the intensity of applied acute aerobic exercise by an á priori stated protocol were included in this moderator analysis.

*Duration of acute aerobic exercise session:* A meta-regression was conducted to investigate the relationship between the duration of acute aerobic exercise and its effect on subsequent interference control. Moreover, we conducted a subgroups analysis, which compared the effect estimate that was pooled from effect sizes with an aerobic exercise session up to 20 min, between 21 and 40 min, and more than 40 min. We used the main exercise duration for analyses without warm-up and without cool-down period.

(2) Characteristics of examined participants

*Participants' age*. Effect sizes were pooled separately within studies that examined preadolescent children (6–12 years of age), adolescents (13–17), young adults (18–35 years of age), middle-aged adults (36–50 years of age), or older adults (aged older than 50 years of age). The allocation of effect sizes to subgroups was conducted based on the reported mean age of participants. A subgroup analysis was conducted to test for statistically significant differences between subgroups' effects.

*Participants' aerobic fitness*. A subgroups analysis was conducted depending on whether examined participants could be classified as lower-fit, average-fit, or higher-fit individuals. Effect sizes were allocated to these fitness levels based on reported peak or maximum oxygen uptake and according to normative data provided by Shvartz and Reibold (1990). Shvartz and Reibold distinguish between seven categories of aerobic fitness ranging from “very poor” to “excellent.” We summarized the categories “very poor” and “poor” to “lower fit.” We summarized the categories “fair” and “average” to “average-fit.” Moreover, we summarized the categories “good,” “very good,” and “excellent” to “higher fit.” Shvartz and Reibold report age dependent curves for the cut-offs for each of their categories separated for gender. If samples were comprised of women and men, we calculated a weighted mean of the cut-off values for men and women and for the mean age of the sample. Weights were assigned according to the proportion of women and men in the sample. Then, we compared the mean peak/maximum oxygen uptake in the sample with the resembled cut-off value and so determined the fitness-category of the sample. In this moderator analysis, only studies that reported peak or maximum oxygen uptake of their participants were included.

(3) Methodological features of existing research

*Comparability of psychosocial stimulation in experimental and control group*. Pooled effect estimates were compared between studies that used a control treatment, in which participants are physically active with a comparable extent of supervision; and studies that used a control treatment, in which participants are physically inactive and without supervision.

*Familiarization with cognitive testing procedure*. A comparison between the following two subgroups was conducted: studies, in which participants had to complete practice trials of the applied interference control test in order to become familiarized with the procedures prior to the actual experiment; studies, in which no familiarization with the applied testing procedures was reported.

*Type of variable that studies used to measure interference control performance*. Studies were classified based on whether they used a comparison between participants' performances at congruent and incongruent test condition, or only participants' performance in incongruent test condition as variable representing participants' interference control performance.

*Delay of Cognitive Testing after Exercise cessation:* A meta-regression was conducted to investigate the relationship between delay of cognitive testing after exercise cessation and the effect of acute aerobic exercise on subsequent interference control. Moreover, we conducted subgroups analysis depending on whether testing of interference control performance was conducted immediately to 15 min or more than 15 min after exercise cessation.

### Data Analysis

Data analysis was conducted with “R” using the “meta” package (Schwarzer et al., 2015). Effect sizes obtained from time-dependent measures and effect sizes obtained from accuracy measures of interference control performance were analyzed separately. Hedges' g corrected standardized mean difference values (SMDs) between exercise and control group at post-intervention were used for analyses. Interpretation of effect sizes followed Cohen's classification. Consequently, SMD values of 0.2, 0.5, and 0.8 were interpreted as small, moderated and large effect sizes, respectively (Cohen, 2013). If a study comprised of several treatment arms eligible for inclusion and if these treatment arms were each tested against the same control group, the sample size of that control group was divided by the number of comparisons to avoid double counting (Higgins and Deeks, 2011). This procedure was carried out to avoid sample size inflation

Heterogeneity in true effect sizes was assumed. Therefore, we used random-effects weights to pool effect sizes. The null hypothesis that the mean true effect is zero was tested using a *z*-test. The variance of true effects (Tau-square) was estimated by calculating *T*^2^ using the weighted method of moments. We used *T*^2^ to estimate the 95% prediction interval. Heterogeneity was further investigated calculating the Higgins' *I*^2^ statistic. The *I*^2^ provides the proportion of true variance in effect sizes over total observed variance. *I*^2^ values of 75, 50, and 25% were interpreted as large, moderate and low proportion of between-study heterogeneity, respectively (Higgins et al., 2003). The null hypothesis of homogeneity of true effects was tested using *Q*-test.

Moderator analyses were conducted using meta-regressions and subgroups analyses. For meta-regression, regression coefficients were tested for statistical significance (different to zero) using Q-tests. For subgroups analyses, a mixed effects model was applied (random-effects within subgroups, fixed-effects between subgroups). Estimates of *T*^2^ within subgroups were pooled, as we assumed that the true study-to-study dispersion was the same in all subgroups (Borenstein et al., 2009). The null-hypothesis that subgroups do not differ was tested with a *Q*-test based on analysis of variance. The proportion of true variance explained by each moderator (*R*^2^) was calculated to describe its influence on the effect of acute aerobic exercise on subsequent interference control performance. In case of a significant *Q*-test and more than two subgroups, *post-hoc* comparisons were conducted.

## Results

### Selected Studies

**Figure 1:**
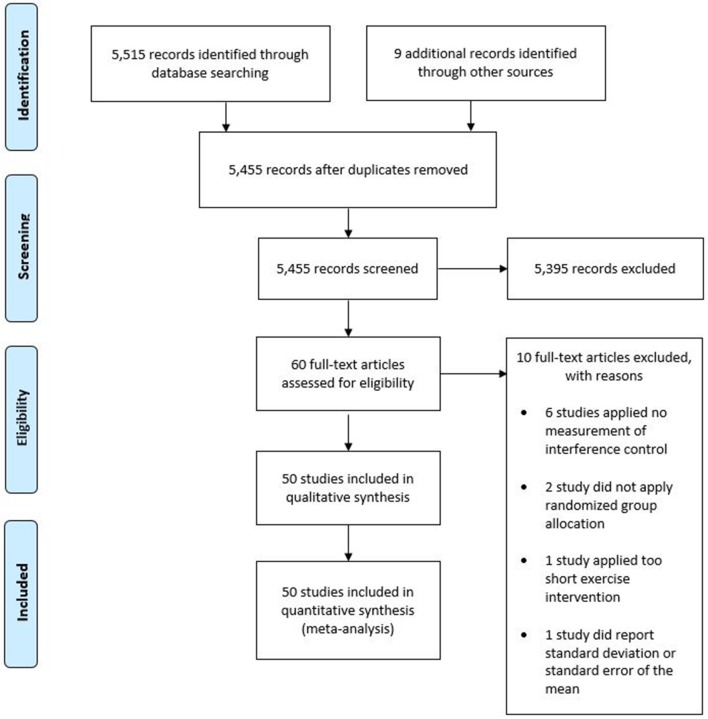
Study selection flow chart according to the PRISMA guidelines.

The final search result was 50 studies included in the qualitative and quantitative synthesis of this review. The included studies contained data from 2,366 participants. Forty of the 50 included studies applied a randomized crossover design, in which participants complete each experimental condition in a randomized order. Therefore, analyses contained 4,446 datasets. Figure 1 provides an overview of the selection process in the form of a PRISMA diagram.

### Characteristics of Included Studies

Of the 50 included studies, eight studies investigated the effects of acute aerobic exercise on subsequent interference control in preadolescent children (mean age: 9.51 years, female: 227, male: 237, 637 datasets), four studies in adolescents (mean age: 15.2 years, female: 56, male: 73, 286 datasets), 30 studies in young adults (mean age: 21.94 years, female: 653, male: 680, 2,874 datasets), and nine studies in older adults (mean age: 63.11 years, female: 308, male: 132, 649 datasets) (note that the number of studies sums up to 51 because Kamijo et al., 2009 examined subsamples of young healthy adults and older adults and reported the results separately). The sample sizes of included studies ranged from 20 to 172. Twelve studies had multiple exercise treatment arms eligible for inclusion in this meta-analysis. Time dependent measures of interference control performance were available for 49 studies. Time-dependent measures reported in included studies were: averaged or absolute reaction time to complete a defined number of trials, or the number of trials participants processed within a defined time-frame. Accuracy measures were only available for 26 studies. Reported accuracy measures were: relative or absolute number of correct answers or errors. None of the included studies applied multiple outcomes of interference control performance. Table 1 presents a comprehensive summary of included studies' characteristics.

**Table 1:** Overview of studies included into meta-analysis on the after-effect of acute aerobic exercise on interference control performance in healthy individuals.

**References**	**Participants' characteristics**	**Study Design**	**Exercise treatment**	**Control group treatment**	**Test procedure**	**Type of variable to measure interference control performance**	**Familiarization with test procedure**
**Preadolescent children**							
Best (2012)	13 (f)/20 (m) 8.1 ± 1.3 y	Cross-over	20 min Marathon Game on Nintendo Wii	20 min watching a video	Flanker task Approximately 5 min after treatment	Comparison between performance at congruent and at incongruent test condition	Familiarization
Chen et al. (2014)	42 (f)/41 (m) 10.32 ± 1.01 y	RCT	30 min of jogging on a playing field at 60–70% of HR_max_	30 min reading	Flanker task Approximately 25 min after treatment	Comparison between performance at congruent and at incongruent test condition	Familiarization
Cooper et al. (2016)	23 (f)/21 (m) 12.6 ± 0.6 y	Cross-over	HIIT with 10x10s maximal sprints interspersed by 50s walking	Resting	Stroop task Immediately after treatment	Performance in incongruent test condition	Familiarization
Egger et al. (2018)	46 (f)/58 (m) 7.93 ± 0.47 y	RCT	20 min running, jumping, spinning to three different songs (each 6 min) with short breaks between each song	20 min listening to audio book	Flanker task immediately after treatment	Comparison between performance at congruent and at incongruent test condition	Familiarization
Hillman et al. (2009)	8 (f)/12 (m) 9.6 ± 0.0.7 y Average-fit (VO_2max_ = 40.1 ± 8.9 ml/kg/min)	Cross-over	20 min walking on a treadmill at 60% of HR_max_	20 min resting	Flanker task After participants' heart rate returned to within 10% of baseline (mean 25.4 ± 6.7 min after treatment)	Comparison between performance at congruent and at incongruent test condition	Familiarization
Jäger et al. (2014)	57 (f)/47 (m) 7.91 ± 0.42 y	RCT	20 min of playful forms of exercise	20 min listening to audio book	Flanker task Immediately after treatment	Comparison between performance at congruent and at incongruent test condition	Familiarization
Pirrie and Lodewyk (2012)	18(f)/22 (m) 9.75 ± 0.36 y	Cross-over	Physical education lesson with different activity games	1 h class period	Stroop task Immediately after treatment Time-point unclear	Performance in incongruent test condition	No familiarization
Drollette et al. (2012)	20 (f)/16 (m) 9.9 ± 0.7 y Average-fit (VO_2max_ = 48.28 ± 6.78 ml/kg/min)	Cross-over	20 min walking on a treadmill at 60% of HR_max_	20 min seated rest	Flanker task 5 min after treatment	Performance in incongruent test condition	Familiarization
Σ: 8	56(f)/73 15.2 y						
**Adolescents**							
Hogan et al. (2013)	11 (f)/19 (m) 14.31 ± 0.56 y	Cross-over	20 min on cycle ergometer at 60% of HR_max_	20 min resting	Flanker task 20 min after treatment	Performance in incongruent test condition	Familiarization
Park and Etnier (2019)	11(f)/11 (m) 15.9 ± 0.29 y	Cross-over	20 min on cycle ergometer at 64–76% of HR_max_ plus 5 min warm-up	20 min reading	Stroop task Immediately after treatment	Performance in incongruent test condition	No familiarization
Peruyero et al. (2017)	21(f)/23 (m) 16.39 ± 0.68 y	Cross-over	20 min of Zumba plus 5 min warm-up and 5 min cool-down	20 min of reading	Stroop task Immediately after treatment	Performance in incongruent test condition	Familiarization
Stroth et al. (2009)	13 (f)/20 (m) 14.2 ± 0.5 y	Cross-over	20 min on cycle ergometer at 60% of HR_max_	20 min resting	Flanker task Immediately after treatment	Performance in incongruent test condition	Familiarization
Σ: 4	56(f)/73 15.2 y						
**Young adults**							
Basso et al. (2015)	51 (f)/34 (m) 22.21 ± 4.15 y	RCT	50 min on cycle ergometer at 85% of HR_max_ plus 5 min for warm-up and 5 min for cool down	60 min watching a video	Stroop task 30, 60, 90, and 120 min after treatment	Comparison between performance at congruent and at incongruent test condition	No familiarization
Brown and Bray (2018)	33 (f)/74 (m) 20 ± 1.87 y	RCT	20 min on cycle ergometer at 10 W, at 65–75% of HR_max_, at 90–90% of HR_max_ or HIIT with 10 x 1 min at 70% of W_peak_ interspersed by 1 min at 12.5% of W_peak_	25 min seated rest	Stroop task Immediately, and 10 min after treatment	Performance in incongruent test condition	Familiarization
Byun et al. (2014)	12 (f)/13 (m) 20.6 ± 1 y Average-fit (VO_2peak_ = 38.3 ± 7.2 ml/kg/min).	Cross-over	10 min on cycle ergometer at 30% of VO_2*peak*_	10 min resting	Stroop task 5 min after treatment	Comparison between performance at congruent and at incongruent test condition	Familiarization
Chang Y. K. et al. (2015)	26 (m) 20.77 ± 0.91 y Average-fit (VO_2peak_ = 42.45 ± 6.49 ml/kg/min)	Cross-over	10, 20, or 45 min on cycle ergometer at 65% of HRR	30 min reading	Stroop task 5 min after treatment	Performance in incongruent test condition	Familiarization
Chang et al. (2017)	13 (f)/17 (m) 22.67 ± 1.52 y Average-fit (VO_2peak_ = 44.52 ± 8.52 ml/kg/min)	Cross-over	20 min on cycle ergometer at 60–70% of HRR plus 5 min for warm-up and 5 min for cool down	30 min reading	Stroop task 15 min after treatment	Performance in incongruent test condition	Familiarization
Crush and Loprinzi (2017)	31 (f)/94 (m) 21.33 ± 2.47 y Average-fit (VO_2max_ = 38.3 – 43.7 ml/kg/min)	RCT	10, 20, 30, 45, or 60 min on treadmill at 40–59% of HRR	Resting	Stroop task	Performance in incongruent test condition	No familiarization
De Marco et al. (2014)	47 (f) 20.43 ± 3.01 y	Cross-over	10 min on treadmill at 70% of HR_max_	10 min of slow walk at 1 km/h	Stroop task Immediately after treatment	Comparison between performance at congruent and at incongruent test condition	No familiarization
Douris et al. (2018)	24 (f)/16 (m) 23.7 ± 1.8 y	RCT	30 min on cycle ergometer at 60–70% of HR_max_	30 min resting	Stroop task Immediately after treatment	Performance in incongruent test condition	No familiarization
Endo et al. (2013)	8 (f)/5 (m) 23 ± 1 y	Cross-over	15 min on cycle ergometer at 20, 40, or 60% of maximum voluntary exercise plus 1 min cool-down	15 min seated rest	Stroop task 5 min after treatment	Performance in incongruent test condition	No familiarization
Finkenzeller et al. (2018)	12 (m) 27.66 ± 7.39 y	Cross-over	Incremental exercise on cycle ergometer starting at 40 W with an increment of 20 W/min until exhaustion (mean duration 16.42 ± 3.34 min)	17 min reading	Flanker task Immediately after treatment	Performance in incongruent test condition	Familiarization
Gothe et al. (2013)	30 (f) 20.1 ± 2 y Lower-fit (VO_2max_ = 35.9 ± 4.8 ml/kg/min)	Cross-over	20 min on treadmill at 60–70% of HR_max_	Two control groups: Baseline 20 min of Yoga	Flanker task Approximately 4 min after treatment	Performance in incongruent test condition	No familiarization
Hillman et al. (2003)	47 (f) 20.5 ± 0.5 y Higher-fit (VO_2max_ = 48.1 ± 13.08 ml/kg/min)	Cross-over	30 min on treadmill at an intensity according to “somewhat hard” to “hard” on the RPE Borg scale	Baseline	Flanker task After participants' heart rate returned to within 10% of baseline (mean 48 min after treatment)	Performance in incongruent test condition	No familiarization
Hwang et al. (2016)	32 (f)/26 (m) 23.57 ± 3.13 y Average-fit (VO_2max_ = 38.12 ± 9.64 ml/kg/min)	RCT	10 min on treadmill at 85–90% of VO_2peak_ plus 2 min warm-up, 5 min increase of intensity and 3 min cool-down	20 min seated rest	Stroop task 10 min after treatment	Comparison between performance at congruent and at incongruent test condition	No familiarization
Kamijo et al. (2007)	12 (m) 25.7 ± 0.7 y	Cross-over	20 min on cycle ergometer at an intensity corresponding to 11, 13, or 15 on the RPE Borg scale with 60 rpm plus 2 min warm-up	Baseline	Flanker task 3 min after treatment	Performance in incongruent test condition	Familiarization
Kamijo et al. (2009)^*^	12 (m) 21.8 ± 0.6 y Higher-fit (VO_2max_ = 52.2 ± 7.27 ml/kg/min)	Cross-over	20 min on cycle ergometer at 30 or 50% of VO_2peak_ and 60 rpm plus 5 min warm-up	Baseline	Flanker task 2 min after treatment	Performance in incongruent test condition	Familiarization
Kao et al. (2017)	37 (f)/27 (m) 19.2 ± 0.8 y Higher-fit (VO_2max_ = 48.6 ± 10 ml/kg/min)	Cross-over	16 min on a treadmill at 60–70% of HR_max_ plus 2 min warm-up and 2 min cool down or HIIT with 1 min warm-up followed by 3 x 1.5 min running 90% of HF_max_ separated by 1 min of walking at 3 mph followed by 1.5 min of cool down	20 min seated rest	Flanker task 20 min after treatment	Comparison between performance at congruent and at incongruent test condition	Familiarization
Kao et al. (2018)	18 (f)/18 (m) 21.5 ± 3.1 y Higher-fit (VO_2max_ = 46.6 ± 10.2 ml/kg/min)	Cross-over	16 min on treadmill at 70% of HR_max_ plus 2 min warm-up and 2 min cool-down or HIIT with 2 min warm-up, followed by 8 x 1 min running at 90% of HR_max_ separated by 1 min of self-paced walking	20 min seated rest	Flanker task Approximately 5 min after treatment	Comparison between performance at congruent and at incongruent test condition	Familiarization
Ligeza et al. (2018)	18 (m) 24.9 ± 2.2 y Higher-fit (VO_2max_ = 50.5 ± 8 ml/kg/min)	Cross-over	24 min on cycle ergometer at 80% of ventilator threshold (mean RPE was 12 ± 1.45) plus 5 min for warm-up and 5 min for cool down or 24 min HIIT (mean RPE was 14.7 ± 1.32) plus 5 min for warm-up and 5 min for cool down	24 min reading	Flanker task Immediately after treatment	Comparison between performance at congruent and at incongruent test condition	Familiarization
Lowe et al. (2014)	23 (f)/11 (m) 20.24 ± 1.76 y	RCT	25 min at 30 or 50% of HRR and 60–70 rpm plus 5 min warm-up and 5 min cool-down	35 min on cycling at very low intensity	Stroop task Immediately after treatment	Comparison between performance at congruent and at incongruent test condition	No familiarization
Lowe et al. (2016)	51 (f) 19.08 ± 1.74 y	RCT	20 min on treadmill at 50% HRR	20 min on treadmill at very low intensity	Stroop task 10 min after treatment	Comparison between performance at congruent and at incongruent test condition	No familiarization
Ludyga et al. (2018)	30 (f)/21 (m) 21.8 ± 1.3 y	Cross-over	15 min running a predefined route in the city at 70% of HR_max_ plus 3 min warm-up and 2 min cool-down	20 min reading	Flanker task Unclear when cognitive testing was administered	Performance in incongruent test condition	Familiarization
Mehren et al. (2019)	Moderate intensity: 16 (f)/16 (m) 29.3 ± 8.5 y	Cross-over	30 min on cycle ergometer at 50–70% of HR_max_ or HIIT lasting 21 min plus 5 min warm-up and 4 min cool-down	30 min watching a movie	Flanker task Immediately after treatment	Comparison between performance at congruent and at incongruent test condition	Familiarization
	Average-fit (VO_2peak_ = 39.6 ± 7.1 ml/kg/min) Vigorous Intensity 16 (f)/15 (m) 28.6 ± 7.7 y Average-fit (VO_2max_ = 37 ± 8.3 ml/kg/min)						
Oberste et al. (2016)	37 (f)/84 (m) 23.81 ± 3.64 y	RCT	30 min on cycle ergometer at 45–50, 65–70, or 85–90% of HR_max_ and 70 rpm plus 5 min warm-up	35 min self-myo-fascial release training	Stroop task 10 min after treatment	Comparison between performance at congruent and at incongruent test condition	Familiarization
O'Leary et al. (2011)	18 (f)/18 (m) 21.2 ± 1.5 y Average-fit (VO_2max_ = 45.2 ± 5.9 ml/kg/min)	Cross-over	20 min on treadmill at 60% of HR_max_	20 min reading	Flanker task After participants' heart rate returned to within 10% of baseline (mean 22.2 ± 0.6 min)	Comparison between performance at congruent and incongruent test condition	Familiarization
Quintero et al. (2018)	*N* = 22 24.6 ± 3.55 y	RCT	HIIT with 4 bouts of 4-min intervals at 85–90% of HR_max_ and RPE 15–17, respectively, interspersed with 4 min of active recovery at 75–85% of HR_max_ and RPE 11–13, respectively, plus 5 min warm-up	Resting	Stroop task 1 min after treatment	Performance in incongruent test condition	Familiarization
Sibley et al. (2006)	37 (f)/42 (m) 22.5 ± 3.1 y	Cross-over	20 min running on treadmill at an intensity corresponding to 11–14 on RPE Borg scale	20 min reading	Flanker task Immediately after treatment	Performance in incongruent test condition	No familiarization
Van Rensburg and Taylor (2008)	8 (f)/15 (m) 23.1 ± 4.6 y	Cross-over	15 min walking on a treadmill plus 2 min warm-up and 1 min cool-down	15 min seated rest	Stroop task Immediately, 5, 10, and 15 min after treatment	Performance in incongruent test condition	No familiarization
Wang et al. (2019)	17 (f)/25 (m) 21.26 ± 1.4 y VO_2peak_ = 44.6 ± 10.81ml/kg/min	Cross-over	20 min on cycle ergometer at 60–70% of HRR plus 5 min war-up and 5 min cool-down	30 min reading	Stroop task 10 min after treatment	Performance in incongruent test condition	Familiarization
Weng et al. (2015)	14 (f)/12 (m) 25.23 ± 2.86 y	Cross-over	30 min on cycle ergometer at 65% of HRR plus 5 min warm-up and 5 min cool-down	30 min on ergo-meter (legs moved mechani-cally)	Flanker task Approximately 6 min after treatment	Performance in incongruent test condition	Familiarization
Yanagisawa et al. (2010)	3 (f)/17 (m) 21.5 ± 4.8 y Average-fit (VO_2peak_ = 46.3 ± 10.4 ml/kg/min)	Cross-over	10 min on cycle ergometer at an intensity corresponding to 50% of VO_2*peak*_	10 min seated rest	Stroop task 15 min after treatment	Comparison between performance at congruent and incongruent test condition	Familiarization
Σ: 30	653(f)/680 Gender of 22 was not reported 21.94 y						
**Older adults**							
Abe et al. (2018)	18 (f)/8 (m) 71.8 ± 4.7 y	Cross-over	10 min stepping while sitting on a chair	Two control groups: 10 min stretching while sitting on a chair 10 min finger movements while sitting on a chair	Stroop task 5 min after treatment	Comparison between performance at congruent and at incongruent test condition	Familiarization
Alves et al. (2012)	42 (f) 52 ± 4.15 y	Cross-over	30 min walking at 50–60% if HRR	10 min talk about benefits of regular exercise on overall health followed by a 15 min stretching session	Stroop task Immediately after treatment	Performance in incongruent test condition	Familiarization
Alves et al. (2014)	13 (f)/9 (m) 53.7 ± 4.15 y	Cross-over	HIIT with 3 min warm-up cycling at 60% of HRR, followed by 10 × 1 min cycling bouts at 80% of HRR separated by 1 min bouts of cycling at 60% of HRR, followed by 2 min cool down cycling at 60% of HRR	10 min talk about benefits of regular exercise on overall health followed by a 15 min stretching session	Stroop task Immediately after treatment	Performance in incongruent test condition	Familiarization
Barella et al. (2010)	32 (f)/8 (m) 69.48 ± 8.32 y	RCT	20 min on treadmill at 60% of HRR plus 5 min warm-up	25 min sitting and general conversation with experimenter	Stroop task Immediately after treatment	Performance in incongruent test condition	Familiarization
Chang Y. et al. (2015)	Higher-fit: 21 (m) 62.76 ± 2.41 y Higher-fit (VO_2max_ = 35.99 ± 2.90 ml/kg/min)	Cross-over	20 min on cycle ergometer at 50–60% of HRR plus 5 min for warm-up and 5 min for cool down	30 min reading	Stroop task 15 min after treatment	Performance in incongruent test condition	Familiarization
	Lower-fit: 21 (m) 63.43 ± 3.34 y Lower-fit (VO_2peak_ = 23.71 ± 1.88 ml/kg/min)						
Chang et al. (2019)	24 (f)/16 (m) 57.58 ± 4.9 y Higher-fit (VO_2peak_ = 37.1 ± 9.88 ml/kg/min)	Cross-over	10, 20, or 45 min on cycle ergometer at 60–70% of HRR plus 5 min for warm-up and 5 min for cool down	30 min reading	Stroop task Immediately after treatment	Performance in incongruent test condition	Familiarization
Chu et al. (2015)	Higher-fit: 12 (f)/10 (m) 63.8 ± 2.3 y Higher-fit (VO_2max_ = 36 ± 1.2 ml/kg/min) Average-fit: 10 (f)/14 (m) 64.9 ± 0.4 y Average-fit (VO_2peak_ = 23.5 ± 2.8 ml/kg/min)	Cross-over	20 min on cycle ergometer at 65% of HRR plus 5 min for warm-up and 5 min for cool down	30 min reading	Stroop task 5 min after treatment	Performance in incongruent test condition	Familiarization
Hyodo et al. (2012)	3 (f)/13 (m) 69.3 ± 3.5 y	Cross-over	10 min on a cycle ergometer at 40–59% of VO_2*max*_	10 min resting	Stroop task 15 min after treatment	Comparison between performance at congruent and at incongruent test condition	Familiarization
Kamijo et al. (2009)^*^	12 (m) 65.5 ± 5.2 y Higher-fit (VO_2max_ = 32.4 ± 4.5 ml/kg/min)	Cross-over	20 min on cycle ergometer at 30 or 50% of VO_2peak_ and 60 rpm plus 5 min warm-up	Baseline	Flanker task 2 min after treatment	Performance in incongruent test condition	Familiarization
Σ: 9	308(f)/132 63.11 y						

*f, female; m, male; RCT, randomized controlled trial; HR_max_, maximum heart rate; W_peak_, peak Watt performance; VO_2_ peak, peak oxygen uptake; HRR, heart rate reserve*.

* *This study investigated young and older adults and reported the results seperately*.

**Figure 2:**
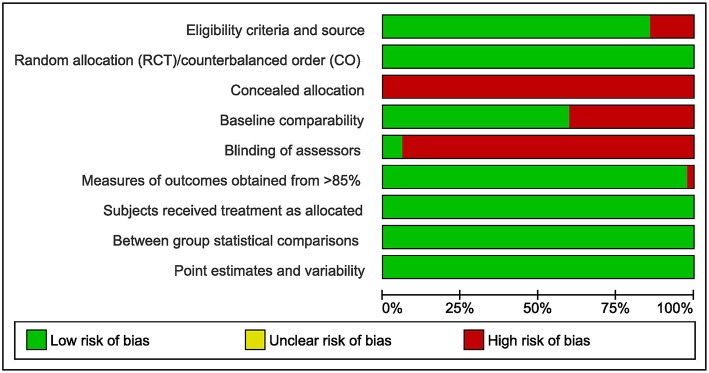
Overview of the PEDro rated study quality (green = low risk of bias, red = high risk of bias, RCT = randomized controlled trial, CO = Crossover trial).

On average, the studies showed a good methodological quality. However, almost no studies provided concealed allocation to treatment groups (RCT) or sequences (Crossover) and blinding of assessors. Figure 2 gives an overview of the PEDro rated study quality. The PEDro ratings for each item and each included study are provided in the Supplementary Material to this study.

### Results of Primary Meta-Analysis

#### Time-Dependent Measures

Concerning time-dependent measures of interference control performance, negative effect size values represent a beneficial effect of acute aerobic exercise compared to control treatment. Forty-nine studies and 87 effect sizes were included in this analysis (4,374 participants). Pooling of effect sizes revealed a small beneficial effect of acute aerobic exercise (Hedges' g = −0.26). The 95% confidence interval ranged from −0.34 to −0.18. As the 95% confidence interval did not cross zero, the average effect was significantly different from zero (*z* = −6.19, *p* < 0.0001). The Q-test revealed significant heterogeneity of true effects (*Q* = 140.59, df = 86, *p* = 0.0002). The calculated *T*^2^ value was 0.0557. The *T*^2^ of 0.0557 yielded a 95% prediction interval of −0.74 to 0.22. The *I*^2^ value was 38.8% with a 95% confidence interval of 20.6–52.9%. The forest plot to these results is provided in the Supplementary Material to this article.

#### Accuracy Measures

For accuracy measures of interference control, positive effect size values stand for beneficial effects of acute aerobic exercise compared to control treatment. Twenty-six studies and 44 effect sizes were included (2,102 participants) in this analysis. The analysis revealed a very small beneficial effect of acute aerobic exercise on subsequent accuracy measures of interference control performance (Hedges' g = 0.13). The 95% confidence interval ranged from almost no effect (0.04) to a small beneficial effect (0.22). The average effect was significantly different from zero (*z* = 2.91, *p* = 0.0037). The *Q*-test of heterogeneity was not statistically significant (*Q* = 36.26, df = 43, *p* = 0.7569). The *T*^2^ was zero. Therefore, the 95% prediction interval was identical to the 95% confidence interval of the mean true effect. The *I*^2^ value was 0% with a 95% confidence interval between 0 and 30.1%. The forest plot to this analysis is also provided in the Supplementary Material to this article.

### Results of Moderator Analyses

Table 2 presents a summary of the moderator analyses. The forest plots to subgroups analyses, which are not depicted in this article, are provided in the Supplementary Material to this article.

(1) Characteristics of the applied acute aerobic exercise session

**Table 2:** Subgroup analyses.

**Sensitivity analyses**	**k**	**Hedges' g**	**95% CI**	**Heterogeneity**	**Test for subgroup difference**	**Explained true variance**
**PRIMARY META-ANALYSIS**						
Time-dependent measures	87	−0.26	−0.34 to −0.18	*T*^2^ = 0.0557, *I*^2^ = 38.8%		
Accuracy measures	44	0.13	0.04 to 0.22	*T*^2^ = 0, *I*^2^ = 0%		
**EXERCISE CHARACTERISTICS**						
**Exercise intensity:**						
*Time-dependent measures:*						
Light	7	−0.29	−0.64 to 0.06	*T*^2^ = 0.064, *I*^2^ = 11.2%	Q_between_ = 9.55, df = 3, *p* = 0.0228	*R*^2^ = 23.89%
Moderate	45	−0.19	−0.29 to −0.08	*T*^2^ = 0.064, *I*^2^ = 4.3%		
Vigorous	18	−0.34	−0.53 to −0.16	*T*^2^ = 0.064, *I*^2^ = 14.6%		
HIIT	5	−0.61	−0.87 to −0.35	*T*^2^ = 0.064, *I*^2^ = 80.1%		
*Accuracy measures:*						
Light	5	−0.03	−0.38 to 0.31	*T*^2^ = 0, *I*^2^ = 0%	Q_between_ = 1.46, df = 3, *p* = 0.6912	*R*^2^ = 0%
Moderate	21	0.14	0.02 to 0.26	*T*^2^ = 0, *I*^2^ = 0%		
Vigorous	12	0.21	0.01 to 0.41	*T*^2^ = 0, *I*^2^ = 1.2%		
HIIT	5	0.14	−0.09 to 0.38	*T*^2^ = 0, *I*^2^ = 0%		
**Exercise duration:**						
*Time-dependent measures:*						
Up to 20 min	57	−0.32	−0.42 to −0.22	*T*^2^ = 0.0538, *I*^2^ = 43.7%	Q_between_ = 4.37, df = 2, *p* = 0.1127	*R*^2^ = 4.98%
21–40 min	19	−0.18	−0.36 to 0	*T*^2^ = 0.0536, *I*^2^ = 43%		
More than 40 min	10	−0.07	−0.32 to 0.18	*T*^2^ = 0.0536, *I*^2^ = 43%		
*Accuracy measures:*						
Up to 20 min	33	0.14	0.03 to 0.24	*T*^2^ = 0, *I*^2^ = 0%	Q_between_ = 0.05, df = 2, *p* = 0.9756	*R*^2^ = 0%
21– 40 min	9	0.12	−0.07 to 0.32	*T*^2^ = 0, *I*^2^ = 0%		
More than 40 min	2	0.09	−0.39 to 0.57	*T*^2^ = 0, *I*^2^ = 0%		
**PARTICIPANTS' CHARACTERISTICS**						
**Age groups:**						
*Time-dependent measures:*						
Preadolescent children	7	−0.48	−0.72 to −0.24	*T*^2^ = 0.0482, *I*^2^ = 84.8%	Q_between_ = 7.83, df = 3, *p* = 0.0498	*R*^2^ = 13.5%
Adolescents	4	−0.37	−0.70 to −0.03	*T*^2^ = 0.0482, *I*^2^ = 64.4%		
Young adults	61	−0.18	−0.28 to −0.08	*T*^2^ = 0.0482, *I*^2^ = 0%		
Older adults	15	−0.39	−0.60 to −0.19	*T*^2^ = 0.0482, *I*^2^ = 52.3%		
*Accuracy measures:*						
Preadolescent children	4	0.20	−0.04 to 0.43	*T*^2^ = 0, *I*^2^ = 0%	Q_between_ = 4.09, df = 3, *p* = 0.2520	*R*^2^ = 0%
Adolescents	1	−0.16	−0.67 to 0.35			
Young adults	30	0.09	−0.02 to 0.20	*T*^2^ = 0, *I*^2^ = 0%		
Older adults	9	0.28	0.07 to 0.49	*T*^2^ = 0, *I*^2^ = 0%		
**Aerobic fitness:**						
*Time-dependent measures:*						
Lower-fit individuals	3	0.02	−0.34 to 0.37	*T*^2^ = 0, *I*^2^ = 0%	Q_between_ = 3.79, df = 2, *p* = 0.1507	*R*^2^ = 0%
Average-fit individuals	28	−0.17	−0.28 to −0.06	*T*^2^ = 0, *I*^2^ = 0%		
Higher-fit individuals	16	−0.32	−0.47 to −0.16	*T*^2^ = 0, *I*^2^ = 0%		
*Accuracy measures:*						
Lower-fit individuals	3	−0.05	−0.42 to 0.33	*T*^2^ = 0.0069, *I*^2^ = 48.4%	Q_between_ = 2.29, df = 2, *p* = 0.3182	*R*^2^ = 10.13%
Average-fit individuals	8	0.26	0.04 to 0.48	*T*^2^ = 0.0069, *I*^2^ = 68%		
Higher-fit individuals	15	0.11	−0.06 to 0.27	*T*^2^ = 0.0069, *I*^2^ = 0%		
**METHODOLOGICAL FEATURES**						
**Type of control group**						
*Time-dependent measures:*						
Comparable	75	−0.27	−0.36 to −0.18	*T*^2^ = 0.0570, *I*^2^ = 69.7%	Q_between_ = 0.20, df = 1, *p* = 0.6583	*R*^2^ = 0%
Not comparable	12	−0.21	−0.44 to 0.01	*T*^2^ = 0.0570, *I*^2^ = 28.6%		
*Accuracy measures:*						
Comparable	7	0.09	−0.12 to 0.29	*T*^2^ = 0, *I*^2^ = 20.5%	Q_between_ = 0.25, df = 1, *p* = 0.6192	*R*^2^ = 0%
Not comparable	37	0.15	0.04 to 0.25	*T*^2^ = 0, *I*^2^ = 0%		
**Familiarization**						
*Time-dependent measures:*						
Familiarization	55	−0.36	−0.46 to −0.26	*T*^2^ = 0.0397, *I*^2^ = 46.2%	Q_between_ = 10.26, df = 1, *p* = 0.0014	*R*^2^ = 28.7%
No Familiarization	32	−0.10	−0.22 to 0.03	*T*^2^ = 0.0397, *I*^2^ = 0%		
*Accuracy measures:*						
Familiarization	40	0.16	0.06 to 0.25	*T*^2^ = 0, *I*^2^ = 0%	Q_between_ = 1.60, df = 1, *p* = 0.2061	*R*^2^ = 0%
No Familiarization	4	−0.03	−0.29 to 0.24	*T*^2^ = 0, *I*^2^ = 51.4%		
**Type of variable**						
*Time-dependent measures:*						
Interference control	28	−0.23	−0.37 to −0.09	*T*^2^ = 0.0570, *I*^2^ = 35.7%	Q_between_ = 0.25, df = 1, *p* = 0.6139	*R*^2^ = 28.7%
Incongruent condition	59	−0.28	−0.38 to −0.17	*T*^2^ = 0.0570, *I*^2^ = 40.8%		
*Accuracy measures:*						
Interference control	12	0.16	0.01 to 0.23	*T*^2^ = 0, *I*^2^ = 0%	Q_between_ = 0.16, df = 1, *p* = 0.6858	*R*^2^ = 0%
Incongruent condition	32	0.12	0.00 to 0.33	*T*^2^ = 0, *I*^2^ = 0%		
**Timing of cognitive testing**						
*Time-dependent measures:*						
Immediately to 15 min delay	72	−0.27	−0.37 to −0.18	*T*^2^ = 0.0611, *I*^2^ = 40.7%	Q_between_ = 0.21, df = 1, *p* = 0.6471	*R*^2^ = 0%
More than 15 min delay	13	−0.22	−0.43 to −0.01	*T*^2^ = 0.0611, *I*^2^ = 38.1%		
*Accuracy measures:*						
Immediately to 15 min delay	37	0.10	0.00 to 0.21	*T*^2^ = 0, *I*^2^ = 0%	Q_between_ = 0.68, df = 1, *p* = 0.4111	*R*^2^ = 0%
More than 15 min delay	6	0.20	0.00 to 0.39	*T*^2^ = 0, *I*^2^ = 0%		

*k, number of effect size estimates; Hedges' g, pooled effect size estimate; CI, confidence interval*.

**Figure 3:**
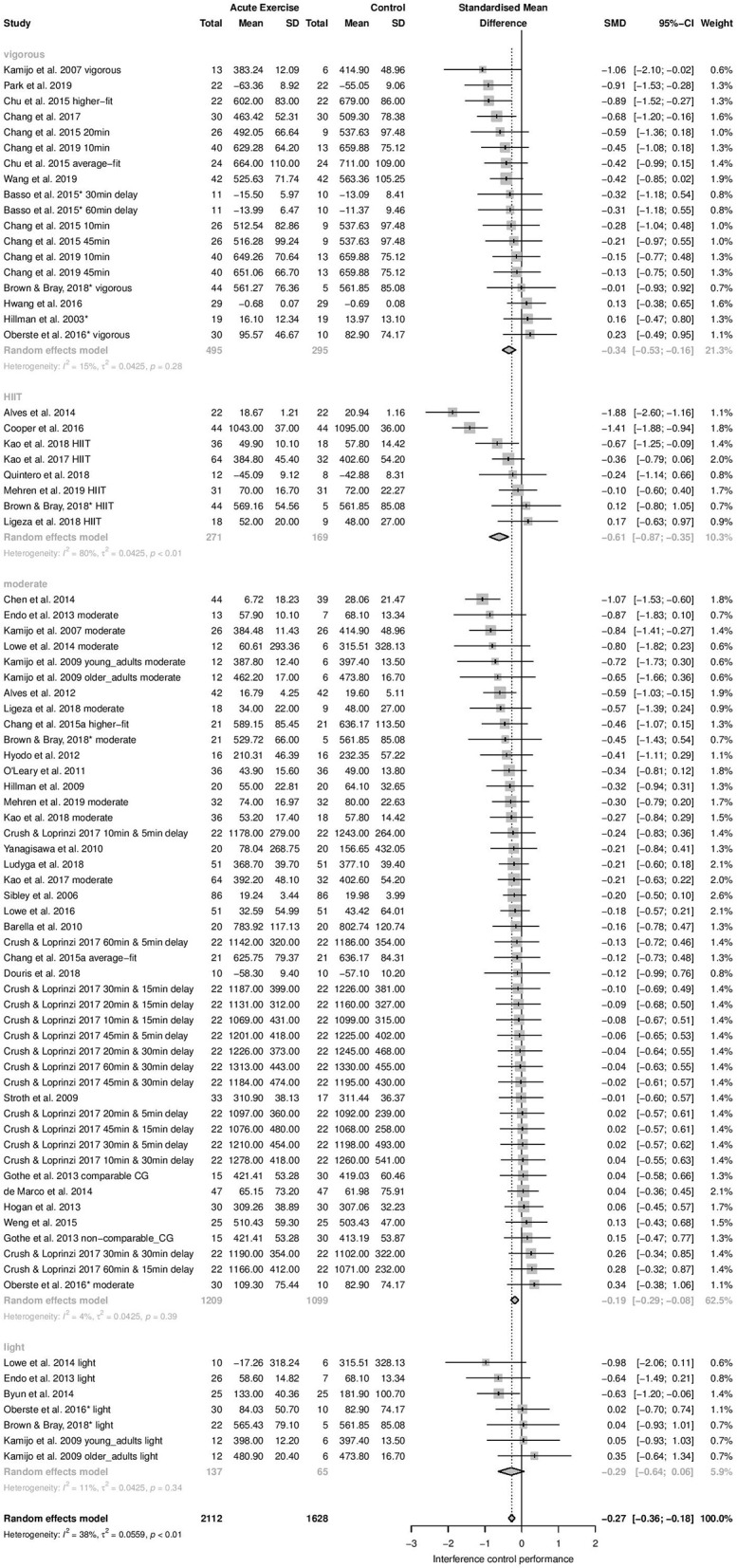
Subgroup Analysis for acute aerobic exercise intensity (time-dependent measures of interference control).

Moderator analysis for exercise intensity:

*Time-dependent measures:* Subgroup analysis revealed significant influence of acute aerobic exercise intensity on the magnitude of effects (Q_between_ = 9.55, df = 3, *p* = 0.0228). Exercise intensity accounted for 26.98% of the true variance. *Post-hoc* pairwise comparisons revealed no significant differences between the effects pooled within light (*k* = 7, Hedges' g = −0.29, 95% CI: −0.64 to 0.06, *T*^2^ = 0.0425, *I*^2^ = 11.2%), moderate (*k* = 45, Hedges' g = −0.19, 95% CI: −0.29 to −0.08, *T*^2^ = 0.0425, *I*^2^ = 4.3%), and vigorous intensity subgroups (*k* = 18, Hedges' g = −0.34, 95% CI: −0.53 to −0.16, *T*^2^ = 0.0425, *I*^2^ = 14.6%) (*p* = 0.1503–0.8056). High-intensity interval training (*k* = 8, Hedges' g = −0.61, 95% CI: −0.87 to −0.35, *T*^2^ = 0.0425, *I*^2^ = 80.1%) did not differ significantly from light (*p* = 0.1503), or vigorous intensity (*p* = 0.1003). However, a significant difference was found between HIIT and moderate intensity (*p* = 0.0031). The forest plot to these results is depicted in Figure 3.

*Accuracy measures:* Subgroups analysis revealed only small differences between pooled effect estimates within studies that used different exercise intensities. The effect pooled within light intensity subgroup was very small detrimental (*k* = 5, Hedges' g = −0.03, 95% CI: −0.38 to 0.31, *T*^2^ = 0, *I*^2^ = 0%). The effect pooled within moderate (*k* = 21, Hedges' g = 0.14, 95% CI: 0.02 to 0.26, *T*^2^ = 0, *I*^2^ = 0%) and the effect pooled within HIIT subgroup (*k* = 5, Hedges' g = 0.14, 95% CI: −0.09 to 0.38, *T*^2^ = 0, *I*^2^ = 0%) were almost identical and very small beneficial. The effect pooled within vigorous intensity subgroup was small beneficial (*k* = 12, Hedges' g = 0.21, 95% CI: 0.01–0.41, *T*^2^ = 0, *I*^2^ = 1.2%), The differences between effect sizes pooled within subgroups did not reach statistical significance (Q_between_ = 1.46, df = 3, *p* = 0.6912). The covariate exercise intensity did not explain any true variance for accuracy measures.

Moderator analysis for exercise duration:

*Time-dependent measures:* Meta-regression showed that longer exercise durations were associated with less beneficial effects of acute aerobic exercise on subsequent interference control performance. The regression coefficient for exercise duration was 0.0067. The regression coefficient slightly failed to reach the threshold for statistical significance (*b* = 0.0067, 95% CI: −0.0004 to 0.0137, *p* = 0.0646). Explained true variance by the covariate “exercise duration” in the meta-regression model was 6.02%. When the studies were clustered in three subgroups (duration up to 20 min vs. duration between 21 and 40 min, more than 40 min), a noticeable difference between effect sizes pooled within subgroups was found. The effect pooled within subgroup of studies, which applied an acute aerobic exercise session of up to 20 min, was −0.32 (*k* = 57, 95% CI: −0.42 to −0.22, *T*^2^ = 0.0538, *I*^2^ = 43.7%). The effect pooled within subgroup of studies, which applied an acute aerobic exercise session between 21 and 40 min, was −0.18 (*k* = 19, 95% CI: −0.36 to 0, *T*^2^ = 0.0538, *I*^2^ = 43%). The effect pooled within subgroup of studies, which applied an acute aerobic exercise session of more than 40 min, was −0.07 (*k* = 10, 95% CI: −0.32 to 0.18, *T*^2^ = 0.0538, *I*^2^ = 0%). However, the differences between subgroups' pooled effect sizes did not reach statistically significance (Q_between_ = 4.37, df = 2, *p* = 0.1127). The categorized covariate of exercise duration accounted for 4.98% of the variance of true effects concerning time-dependent measures.

*Accuracy measures*: Meta-regression showed only a very weak, non-significant association between exercise duration and magnitude of effects (smaller beneficial effects with longer exercise durations) (*b* = −0.0026, 95% CI: −0.0139 to 0.0086, *p* = 0.7290, *R*^2^ = 0%). When the studies were clustered in the three subgroups, the effects pooled within subgroups hardly differed from each other. The effect pooled within subgroup of studies, which applied an acute aerobic exercise session of up to 20 min, was 0.14 (*k* = 33, 95% CI: 0.03–0.24, *T*^2^ = 0, *I*^2^ = 0%). The effect pooled within subgroup of studies, which applied an acute aerobic exercise session between 21 and 40 min, was 0.12 (*k* = 9, 95% CI: −0.07 to 0.32, *T*^2^ = 0, *I*^2^ = 0%). The effect pooled within subgroup of studies, which applied an acute aerobic exercise session of more than 40 min, was 0.09 (*k* = 2, 95% CI: −0.39 to 0.57, *T*^2^ = 0, *I*^2^ = 0%). The differences between subgroups' pooled effect sizes did not reach statistically significance (Q_between_ = 0.05, df = 2, *p* = 0.9756). The covariate exercise duration did not explain any true variance for accuracy measures.

(2) Characteristics of examined participants

**Figure 4:**
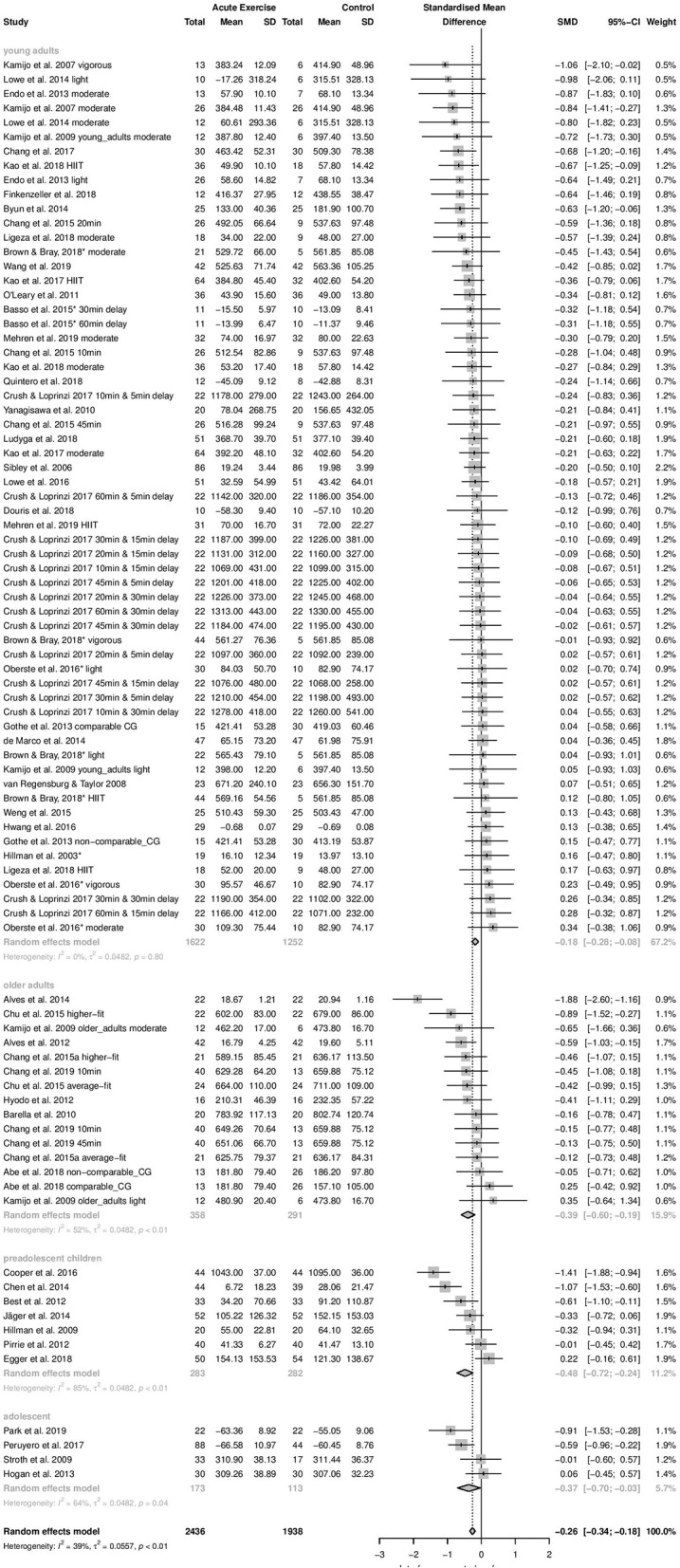
Subgroup Analysis for age group (time-dependent measures of interference control).

Moderator analysis for participants' age group:

*Time-dependent measures:* In this subgroups analysis, the smallest effect was pooled within subgroup of young adults (*k* = 61, Hedges' g = −0.18, 95% CI:−0.28 to −0.08, *T*^2^ = 0.0482, *I*^2^ = 0%). On the contrary, effects pooled within preadolescent children (*k* = 7, Hedges' g = −0.48, 95% CI:−0.72 to −0.24, *T*^2^ = 0.0482, *I*^2^ = 84.8%), within adolescents (*k* = 4, Hedges' g = −0.37, 95% CI:−0.70 to −0.03, *T*^2^ = 0.0482, *I*^2^ = 64.4%), and within older adults (*k* = 15, Hedges' g = −0.39, 95% CI: −0.60 to −0.19, *T*^2^ = 0.0482, *I*^2^ = 52.3%) were almost moderate. The difference between subgroups' effect sizes was statistically significant (Q_between_ = 7.83, df = 3, *p* = 0.0498). Age group explained 13.5% of the true variance. *Post-hoc* analyses revealed a significant difference between subgroups young adults and preadolescent children (*p* = 0.0237). The other subgroups did not differ significantly from each other (*p* = 0.0768–0.9202). The forest plot to these results is depicted in Figure 4.

*Accuracy measures:* Small beneficial effects were revealed within subgroup of preadolescent children (*k* = 4, Hedges' g = 0.20, 95% CI: −0.04 to 0.43, *T*^2^ = 0, *I*^2^ = 0%) and within subgroup of older adults subgroup (*k* = 9, Hedges' g = 0.28, 95% CI: 0.07–0.49, *T*^2^ = 0, *I*^2^ = 0%). Adolescents subgroup comprised of only one effect size (*k* = 1, Hedges' g = −0.16, 95% CI: −0.66 to 0.36). Effect estimate pooled within subgroup of young adults was very small beneficial (*k* = 30, Hedges' g = 0.09, 95% CI: −0.02 to 0.20, *T*^2^ = 0, *I*^2^ = 0%). The difference between the effect sizes that were pooled within subgroups did not reach statistical significance (Q_between_ = 4.09, df = 3, *p* = 0.2520). The covariate age group explained no true variance.

Moderator analysis for participants' aerobic fitness:

*Time-dependent measures:* Effect sizes within aerobic fitness subgroups increased with increasing aerobic fitness level. Effect sizes pooled within lower-fit, average-fit, and higher-fit subgroup were 0.02 (*k* = 3, 95% CI:−0.34 to 0.37, *T*^2^ = 0, *I*^2^ = 0%), −0.17 (*k* = 28, 95% CI:−0.28 to −0.06, *T*^2^ = 0, *I*^2^ = 0%), and −0.32 (*k* = 16, 95% CI:−0.47 to −0.16, *T*^2^ = 0, *I*^2^ = 0%), respectively. The differences between effect size estimates pooled within subgroups did not reach statistical significance (Q_between_ = 3.79, df = 2, *p* = 0.1507) and the covariate aerobic fitness did not explain true variance.

*Accuracy measures:* Analysis revealed a small detrimental effect pooled within subgroup of lower-fit individuals (*k* = 3, Hedges' g = −0.05, 95% CI: −0.42 to 0.33, *T*^2^ = 0.0069, *I*^2^ = 48.4%). A small beneficial effect was revealed within subgroup of average-fit individuals (*k* = 8, Hedges' g = 0.26, 95% CI: 0.04–0.48, *T*^2^ = 0.0069, *I*^2^ = 68%). A very small beneficial effect was revealed within subgroup of higher-fit individuals (*k* = 15, Hedges' g = 0.11, 95% CI: −0.06 to 0.27, *T*^2^ = 0.0069, *I*^2^ = 0%). The differences between subgroups' effect sizes did not reach statistical significance (Q_between_ = 2.29, df = 2, *p* = 0.3182). The covariate explained 1.13% of true variance.

(3) Methodological features of existing research

Moderator analysis for comparability of psychosocial stimulation in experimental and control group:

*Time-dependent measures:* Subgroups analysis revealed a slightly more beneficial effect pooled within subgroup of studies that used a physically inactive and non-supervised control treatment as comparison to acute aerobic exercise (*k* = 75, Hedges' g = −0.27, 95% CI: −0.36 to −0.18, *T*^2^ = 0.0570, *I*^2^ = 28.6%). The effect size pooled within subgroup of studies that used a physically active and supervised control treatment as comparison to acute aerobic exercise was −0.21 (*k* = 12, 95% CI: −0.44 to 0.01, *T*^2^ = 0.0570, *I*^2^ = 69.7%). The difference between subgroups did not reach statistical significance (Q-between = 0.20, df = 1, *p* = 0.6583). The covariate did not explain true variation.

*Accuracy Measures:* In both subgroups, pooled effects were very small beneficial (comparable CG: *k* = 7, Hedges' g = 0.09, 95% CI: −0.12 to 0.29, *T*^2^ = 0, *I*^2^ = 20.5%; not comparable CG (*k* = 37, Hedges' g = 0.15, 95% CI: 0.04–0.25, *T*^2^ = 0, *I*^2^ = 0%). The differences between subgroups' effect sizes did not reach statistical significance (Q_between_ = 0.25, df = 1, *p* = 0.6192) and no true variance was explained.

Moderator analysis for familiarization with cognitive testing procedure':

*Time-dependent measures:* Pooling of effect sizes derived from studies that applied practice trials of the testing procedure before beginning of the actual experiment revealed a small to moderate beneficial effect (*k* = 55, Hedges' g = −0.36, 95% CI: −0.46 to −0.26, *T*^2^ = 0.00397, *I*^2^ = 46.2%). At the same time, pooling of effect sizes derived from studies that did not familiarize their participants with the testing procedure before beginning of the experiment revealed only a very small beneficial effect (*k* = 32, Hedges' g = −0.10, 95% CI: −0.22 to 0.03). The difference between subgroups' pooled effect was statistical significant (Q_between_ = 10.26, df = 1, *p* = 0.0014). The covariate explained 28.7% of the true variance.

*Accuracy measures:* The effect sizes pooled from studies that applied practice trials of the testing procedure before beginning of the actual experiment revealed a small beneficial effect (*k* = 40, Hedges' g = 0.16, 95% CI: 0.06–0.25, *T*^2^ = 0, *I*^2^ = 0%). On the contrary, if no familiarization was applied, a very small detrimental effect was found (*k* = 4, Hedges' g = −0.03, 95% CI: −0.29 to 0.24, *T*^2^ = 0, *I*^2^ = 51.4%). However, the differences between subgroups' effect sizes did not reach statistical significance for accuracy measures (Q_between_ = 1.60, df = 1, *p* = 0.2061). No true variance was explained by this covariate.

Moderator analyses for the type of variable that studies used to measure interference control:

*Time-dependent measures:* Data aggregated from studies that used a comparison between participants' performance in congruent and in incongruent test condition as variable representing participants' interference control performance revealed a small beneficial effect (*k* = 28, Hedges' g = −0.23, 95% CI: −0.37 to −0.09, *T*^2^ = 0.0570, *I*^2^ = 35.7%). At the same time, the effect pooled from data derived from studies that used only participants' performance in incongruent test condition was slightly larger (*k* = 59, Hedges' g = −0.28, 95% CI: −0.38 to −0.17, *T*^2^ = 0.0570, *I*^2^ = 40.8%). Effect estimates pooled within subgroups' did not differ significantly from each other (Q-between = 0.25, df = 1, *p* = 0.6139). No true variance was explained by this covariate.

*Accuracy measures:* Both subgroups showed almost identical pooled effect estimates (interference control variable: *k* = 12, Hedges' g = 0.16, 95% CI: 0.00–0.33, *T*^2^ = 0, *I*^2^ = 0%; only incongruent condition as variable: *k* = 32, Hedges' g = 0.12, 95% CI: 0.01–0.23, *T*^2^ = 0, *I*^2^ = 0%) (Q_between_ = 0.16, df = 1, *p* = 0.6858). The covariate did not explain true variance.

Moderator Analysis for delay between exercise cessation and cognitive testing:

*Time-dependent measures:* Meta-regression showed that a shorter delay after exercise cessation until beginning of cognitive testing leads to larger beneficial effects of acute aerobic exercise on subsequent interference control performance. The regression coefficient of delay between exercise cessation and cognitive testing was 0.0064. However, this regression coefficient did not reach statistical significance (*b* = 0.0064, 95% CI: −0.0013 to 0.0140, *p* = 0.1046). Explained true variance by the covariate “delay between exercise cessation and cognitive testing” in the meta-regression model was only 2.92%. When the studies were clustered in two subgroups (immediately to 15 min vs. more than 15 min after exercise cessation) effect sizes pooled within subgroups hardly differed from each other. Effect size pooled within subgroup of studies that applied cognitive immediately to 15 min after exercise cessation was −0.27 (*k* = 72, 95% CI: −0.37 to −0.18, *T*^2^ = 0.0611, *I*^2^ = 40.7%). Effect size pooled within subgroup of studies that applied cognitive more than 15 min after exercise cessation was −0.22 (*k* = 13, 95% CI: −0.43 to −0.01, *T*^2^ = 0.0611, *I*^2^ = 38.1%). Effects pooled within subgroups did not differ significantly from each other (Q_between_ = 0.21, df = 1, *p* = 0.6471). The categorical covariate did not explain true variance.

*Accuracy measures:* Meta-regression revealed hardly any association between delay and effect size magnitude for accuracy measures of interference control performance (*b* = 0.0016, 95% CI: −0.0076 to 0.0108, *p* = 0.7378, *R*^2^ = 0%). When subgroups analysis was conducted, the difference was also very small (immediately to 15 min: *k* = 37, Hedges' g = 0.10, 95% CI: 0.00–0.21, *T*^2^ = 0.0, *I*^2^ = 0%, more than 15 min: *k* = 6, Hedges' g = 0.20, 95% CI: 0.00–0.39, *T*^2^ = 0.0, *I*^2^ = 0%). The difference between effect sizes pooled within subgroups did not reach statistical significance (Q_between_ = 0.68, df = 1, *p* = 0.4111). No true variation was explained by the categorical covariate.

## Discussion

This meta-analysis, among other things, showed that acute aerobic exercise facilitates subsequent interference control performance. Pooling of included studies' effect sizes revealed that performance in time-dependent and in accuracy measures, significantly improved after acute aerobic exercise compared to control treatment. However, the effect that was pooled within time-dependent measures was noticeably bigger than the effect that was pooled within accuracy measures (0.26 vs. 0.13). One possible explanation for this difference is the occurrence of ceiling effects, when accuracy measures were applied to measure interference control performance. It has been reported that healthy individuals tend to reach, or come close to, 100% accuracy in the Flanker/Stroop task (Wöstmann et al., 2013). Flanker and Stroop task performance accuracy close to 100%, significantly diminishes test score variance in participants, and consequently the discriminative power of these tests. In the studies that were included in this review, participants in the control groups already reached an average accuracy of 91.95% (SD = 3.95) in incongruent Flanker/Stroop condition. This excellent accuracy performance of control group participants did not leave much space for accuracy advantages following acute exercise. The fact that the current analysis did not detect heterogeneity of effect sizes for accuracy measures, further supports the assumption that ceiling effects were responsible for the smaller effects in accuracy measures compared to time-dependent measures.

One may argue that benefits of acute aerobic exercise on subsequent interference control performance of small size are with limited practical relevance. However, effect sizes from time-dependent measures broadly varied. Heterogeneity was significant. The prediction interval ranged from −0.74 to 0.22. This means that, depending on covariate values, acute aerobic exercise has the potential to improve subsequent interference control performances quite noticeably.

In the here conducted moderator analyses, the magnitude of exercise-induced interference control performance benefits was significantly influenced by exercise intensity, participants' age, and familiarization with cognitive testing procedure. Concerning exercise intensity, vigorous intensity (−0.34) and HIIT (−0.61) were shown most effective. On the contrary, acute aerobic exercise of moderate intensity yielded only a small beneficial effect (−0.19). These findings contradict assumptions that exercise intensity and subsequent higher cognitive performances show an inverted-U relationship (McMorris, 2008; Chang, 2009). Finding HIIT most effective is in line with recent results about the physiological adaptations to acute HIIT sessions within the central nervous system. Kao et al. (2017) showed a smaller and more efficient P3 component following acute HIIT compared to following acute and continuous exercise with moderate intensity. Acute sessions of HIIT are associated with higher brain-derived neurotrophic factor expression compared to acute, continuous exercise with moderate intensity (Jiménez-Maldonado et al., 2018). However, enthusiasm about HIIT as an option to improve interference control performance on a large scale is still premature. In the HIIT subgroup, large heterogeneity remained unexplained. Potentially, the differences in HIIT regimens are decisive for its effects on subsequent interference control performance. Therefore, future studies should further investigate the differential effects of different variants of HIIT on subsequent interference control performance.

Concerning participants' age, we found pronounced effects in preadolescent children (−0.48), adolescents (−0.37), and older adults (−0.39). On the contrary, in young adults, only a small beneficial effect was revealed (−0.18). This finding is in line with the results of Ludyga and colleagues. In their meta-analysis, they found an inverted-U shaped relationship between participants‘ age and the effect of acute moderate exercise on executive function performance. The pronounced effects in preadolescent children and adolescents should encourage usage of acute exercise sessions in school context. Interference control is decisive for learning activities (Diamond, 2013). First evidence shows that sequences of classical classroom lesson interspersed by short sessions of exercise can improve school learning (Budde et al., 2008). It should be noted, however, that within preadolescent children subgroup, large heterogeneity remained unexplained (*I*^2^ = 84.8%). Future studies should investigate the influence of potential covariates within this subgroup to clarify the variation of effect sizes.

Moderator analyses dismissed some doubts that the reported beneficial effects of acute aerobic exercise on subsequent interference control are due to methodological shortcomings. If familiarization was applied (−0.36), participants showed bigger beneficial effects compared to lack of familiarization (−0.10). It was criticized that beneficial effects of acute aerobic exercise on subsequent interference control are actually due to facilitation of learning, rather than improvement of interference control performance. The present result contradicts this assumption.

Moderator analyses of exercise duration and participants' aerobic fitness failed to reach statistical significance. However, this might be due to low statistical test power. Empirically, both covariates showed noticeable influence on the effect of acute aerobic exercise on subsequent time-dependent measures of interference control. When the studies were clustered in duration subgroups (duration up to 20 min vs. duration between 21 and 40 min vs. more than 40 min), subgroups with shorter exercise duration apparently yielded more beneficial effects (−0.32 vs. −0.18 vs. −0.07). If all available information (absolute value of exercise duration) was included in the analysis (meta-regression), the positive relationship between exercise duration and magnitude of effects became even clearer. The tendency for bigger beneficial effects with shorter exercise duration is in line with findings of recent primary studies. These showed advantages of exercise duration of up to 20 min compared to duration of more than 20 min on subsequent interference control performance (Chang Y. K. et al., 2015; Chang et al., 2019). One explanation for the advantageous effects of shorter duration is less exercise-induced fatigue and dehydration. Exercise-induced fatigue and dehydration were shown to have detrimental effects on subsequent cognitive performances (Cian et al., 2001). Future studies should further investigate the influence of exercise duration on the effects of acute aerobic exercise on subsequent interference control performance. One interesting research question certainly is the minimum duration for exercise-induced benefits.

Concerning participants' aerobic fitness level, it was hypothesized above, that lower fitness levels are associated with bigger effects of exercise on subsequent interference control performance. Results of subgroup analysis, however, revealed the opposite. Individuals with higher aerobic fitness (−0.32) benefitted more than individuals with average fitness (−0.17). For individuals with lower aerobic fitness, average effect size even showed no beneficial effect (0.02). Potentially, individuals with higher aerobic fitness experienced less detrimental effects of exercise-induced fatigue. Thus, the positive adaptations to acute exercise could result in interference control benefits.

The present meta-analysis dismissed remaining doubts that the beneficial effects of acute aerobic exercise on subsequent interference control performance are a result of expectation-driven placebo effects. Differences in applied control group treatment (physically inactive and without supervision vs. physically active and with supervision) did not explain dispersion in effects. This result is in line with a recent study of our group, which investigated the expectations of participants toward the effects of exercise and control group treatments on subsequent Stroop performance. In this study, no differences of expectations for cognitive benefits between exercise, passive, and active control group treatments were found (Oberste et al., 2017). Within the subgroup of studies that used a physically active and supervised control treatment as comparison to acute aerobic exercise, large heterogeneity remained unexplained (*I*^2^ = 69.7%). Future studies should further investigate the question of an optimal control group paradigm for studies that examine the effects of acute exercise on subsequent cognition.

Regarding the type of variable (only incongruent condition vs. interference control variable), only a weak influence on exercise-induced interference control benefits was revealed. This result dismisses above-explained doubts that beneficial effects of acute aerobic exercise on subsequent Stroop/Flanker performance are due to basic information processing improvements instead of interference control improvements. The fact that no noticeable difference was found in effect sizes between studies that used either type of variable, rather indicates no effects of acute exercise on subsequent basic information processing speed. If both, interference control and basic information processing speed, would benefit from preceding exercise, stronger benefits could be expected for studies reporting only participants' incongruent condition performance. This is due to the fact that performance in the incongruent test condition of the Flanker/Stroop task is determined by the capacity of interference control, but also by the efficiency of basic information processing (Stroop, 1935; Eriksen and Eriksen, 1974; Golden, 1978). Possibly, acute aerobic exercise improves only higher cognitive performances. Future studies and meta-analyses should investigate the question whether exercise induced benefits in healthy individuals are specific or rather general.

The delay between exercise cessation and the start of cognitive testing showed only a weak association with the magnitude of effect sizes. Slightly higher effects were found within subgroup of studies that applied cognitive testing immediately to 15 min after exercise cessation (−0.27). The effect in studies that applied cognitive testing more than 15 min after exercise cessation was −0.22. One should note, however, that only studies that applied cognitive testing up to 60 min after exercise cessation were included. Therefore, the results indicate that benefits of acute aerobic exercise on interference control sustain within the first hour after exercise.

The here presented results must be interpreted against the background of limitations. In general, the included studies were of low risk of bias. However, almost no studies provided concealed allocation and blinding of assessors (see Figure 2). Therefore, the impact of these two methodological shortcomings could not be investigated in the present meta-analysis. Concerning moderator analyses, one should note that meta-analytic subgroup analyses are generally observational. In a randomized controlled trial, participants are assigned at random to treatment groups. Randomized group allocation provides control of confounding variables. Therefore, differences in the dependent variable can be attributed to the experimental treatment. In subgroup-analyses, however, the allocation of studies into subgroups is *post-hoc* and not at random. Thus, systematic bias due to confounders cannot be ruled out. Conclusions drawn from moderator analyses should always be interpreted with caution. The present meta-analysis did not provide evidence on potential interaction effects between covariates on exercise-induced interference control benefits. It remains unclear, if, for e.g., HIIT is equally effective to improve subsequent interference control in young adults as it is in older adults.

## Conclusion

Acute aerobic exercise improves subsequent interference control performance. However, several covariates determine the magnitude of that effect. It was revealed that higher exercise intensities (vigorous intensity and HIIT), also participants at younger or older age, and participants who are familiar with the testing procedure benefit most from acute aerobic exercise. Potentially, shorter exercise durations and higher aerobic fitness might also lead to advantageous effects of acute aerobic exercise on subsequent interference control performance. At the same time, evidence provided in this review indicate that the beneficial effects of acute aerobic exercise on subsequent interference control performance are unlikely a result of bias due to methodological shortcomings. The fact that large heterogeneity remained unexplained in HIIT, preadolescent children, and active and supervised control group subgroups indicates the need for further research on covariates within these subgroups. It should be noted that small effect sizes were observed for all analyses.

## Author Contributions

MO, FJ, WB, and PZ planned the project. MO and SS conducted literature search, data extraction, and risk of bias assessment. Inconsistencies were resolved in consultation with FJ. MO, NJ, DW, and FJ conducted data analyses. WB, PZ, FJ, and DW communicated with study authors in case of missing data. MO wrote the manuscript. WB supervised MO concerning underlying neurobiological effects of acute aerobic exercise. All authors proofread the manuscript.

### Conflict of Interest

The authors declare that the research was conducted in the absence of any commercial or financial relationships that could be construed as a potential conflict of interest.
