# Supplementary material for: Effects and Moderators of Acute Aerobic Exercise on Subsequent Interference Control: A Systematic Review and Meta-Analysis
Source: Front Psychol. 2019 Nov 21;10:2616. doi: 10.3389/fpsyg.2019.02616 (PMC6881262; doi:10.3389/fpsyg.2019.02616)
Supplement: Supplementary file 1 [file Data_Sheet_1.pdf]

Supplementary Material

1 PRISMA Checklist

Section/topic	#	Checklist item	Reported on page #
**TITLE**			
Title	1	Identify the report as a systematic review, meta-analysis, or both.	p. 1
**ABSTRACT**			
Structured summary	2	Provide a structured summary including, as applicable: background; objectives; data sources; study eligibility criteria, participants, and interventions; study appraisal and synthesis methods; results; limitations; conclusions and implications of key findings; systematic review registration number.	p. 2
**INTRODUCTION**			
Rationale	3	Describe the rationale for the review in the context of what is already known.	pp. 3-4
Objectives	4	Provide an explicit statement of questions being addressed with reference to participants, interventions, comparisons, outcomes, and study design (PICOS).	pp. 7-6
**METHODS**			
Protocol and registration	5	Indicate if a review protocol exists, if and where it can be accessed (e.g., Web address), and, if available, provide registration information including registration number.	p. 7
Eligibility criteria	6	Specify study characteristics (e.g., PICOS, length of follow-up) and report characteristics (e.g., years considered, language, publication status) used as criteria for eligibility, giving rationale.	pp. 7-8
Information sources	7	Describe all information sources (e.g., databases with dates of coverage, contact with study authors to identify additional studies) in the search and date last searched.	p. 8
Search	8	Present full electronic search strategy for at least one database, including any limits used, such that it could be repeated.	p. 8-9
Study selection	9	State the process for selecting studies (i.e., screening, eligibility, included in systematic review, and, if applicable, included in the meta-analysis).	pp. 8-9

Data collection process	10	Describe method of data extraction from reports (e.g., piloted forms, independently, in duplicate) and any processes for obtaining and confirming data from investigators.	p. 9
Data items	11	List and define all variables for which data were sought (e.g., PICOS, funding sources) and any assumptions and simplifications made.	p. 9
Risk of bias in individual studies	12	Describe methods used for assessing risk of bias of individual studies (including specification of whether this was done at the study or outcome level), and how this information is to be used in any data synthesis.	pp- 9-10
Summary measures	13	State the principal summary measures (e.g., risk ratio, difference in means).	pp. 13-14
Synthesis of results	14	Describe the methods of handling data and combining results of studies, if done, including measures of consistency (e.g., I^2) for each meta-analysis.	pp. 13-14

Section/topic	#	Checklist item	Reported on page #
Risk of bias across studies	15	Specify any assessment of risk of bias that may affect the cumulative evidence (e.g., publication bias, selective reporting within studies).	p. 14
Additional analyses	16	Describe methods of additional analyses (e.g., sensitivity or subgroup analyses, meta-regression), if done, indicating which were pre-specified.	p. 14
**RESULTS**			
Study selection	17	Give numbers of studies screened, assessed for eligibility, and included in the review, with reasons for exclusions at each stage, ideally with a flow diagram.	p. 14, Figure 1
Study characteristics	18	For each study, present characteristics for which data were extracted (e.g., study size, PICOS, follow-up period) and provide the citations.	pp. 14-15 and Table 1
Risk of bias within studies	19	Present data on risk of bias of each study and, if available, any outcome level assessment (see item 12).	Figure 2, Supplementary material
Results of individual studies	20	For all outcomes considered (benefits or harms), present, for each study: (a) simple summary data for each intervention group (b) effect estimates and confidence intervals, ideally with a forest plot.	Table 1, Supplementary material
Synthesis of results	21	Present results of each meta-analysis done, including confidence intervals and measures of consistency.	pp. 15-22, Figures 3 & 4 Supplementary

			Material
Risk of bias across studies	22	Present results of any assessment of risk of bias across studies (see Item 15).	pp. 14-15 Figure 2 Supplementary material
Additional analysis	23	Give results of additional analyses, if done (e.g., sensitivity or subgroup analyses, meta-regression [see Item 16]).	pp. 16-22
**DISCUSSION**			
Summary of evidence	24	Summarize the main findings including the strength of evidence for each main outcome; consider their relevance to key groups (e.g., healthcare providers, users, and policy makers).	p. 22
Limitations	25	Discuss limitations at study and outcome level (e.g., risk of bias), and at review-level (e.g., incomplete retrieval of identified research, reporting bias).	pp. 27-28
Conclusions	26	Provide a general interpretation of the results in the context of other evidence, and implications for future research.	p. 28
**FUNDING**			
Funding	27	Describe sources of funding for the systematic review and other support (e.g., supply of data); role of funders for the systematic review.	p. 29

From: Moher D, Liberati A, Tetzlaff J, Altman DG, The PRISMA Group (2009). Preferred Reporting Items for Systematic Reviews and Meta-Analyses: The PRISMA Statement. PLoS Med 6(7): e1000097. doi:10.1371/journal.pmed1000097

For more information, visit: **www.prisma-statement.org**.

2 Supplementary Figures

	Eligibility criteria and source	Random allocation (RCT)/counterbalanced order (CO)	Concealed allocation	Baseline comparability	Blinding of assessors	Measures of outcomes obtained from >85%	Subjects received treatment as allocated	Between group statistical comparisons	Point estimates and variability
Abe et al. 2018	+	+	−	+	−	+	+	+	+
Alves et al. 2012	+	+	−	+	−	+	+	+	+
Alves et al. 2014	+	+	−	+	−	+	+	+	+
Barella et al. 2010	+	+	−	+	−	+	+	+	+
Basso et al. 2015	+	+	−	+	−	+	+	+	+
Best 2012	+	+	−	−	−	+	+	+	+
Brown & Bray 2018	+	+	−	+	−	+	+	+	+
Byun et al. 2014	+	+	−	+	−	+	+	+	+
Chang et al. 2015	+	+	−	−	−	+	+	+	+
Chang et al. 2015a	+	+	−	+	−	+	+	+	+
Chang et al. 2017	+	+	−	−	−	+	+	+	+
Chang et al. 2019	+	+	−	+	−	+	+	+	+
Chen et al. 2014	+	+	−	+	−	+	+	+	+
Chu et al. 2015	+	+	−	+	−	+	+	+	+
Cooper et al. 2016	−	+	−	+	−	+	+	+	+
Crush & Loprinzi 2017	+	+	−	+	−	+	+	+	+
de Marco et al. 2014	+	+	−	−	−	+	+	+	+
Douris et al. 2018	+	+	−	+	−	+	+	+	+
Drollette et al. 2012	+	+	−	+	−	+	+	+	+
Egger et al. 2018	−	+	−	+	+	+	+	+	+
Endo et al. 2013	+	+	−	+	−	+	+	+	+
Finkenzeller et al. 2018	+	+	−	+	−	+	+	+	+
Gothe et al. 2013	+	+	−	−	−	+	+	+	+
Hillman et al. 2003	+	+	−	−	−	+	+	+	+
Hillman et al. 2009	+	+	−	+	−	+	+	+	+
Hogan et al. 2013	+	+	−	−	−	+	+	+	+
Hwang et al. 2016	+	+	−	+	−	+	+	+	+
Hyodo et al. 2012	+	+	−	+	−	+	+	+	+
Jäger et al. 2014	−	+	−	+	+	+	+	+	+
Kamijo et al. 2007	+	+	−	−	−	+	+	+	+
Kamijo et al. 2009	+	+	−	−	−	−	+	+	+
Kao et al. 2017	+	+	−	−	−	+	+	+	+
Kao et al. 2018	+	+	−	−	−	+	+	+	+
Ligeza et al. 2018	+	+	−	−	−	+	+	+	+
Lowe et al. 2014	+	+	−	−	−	+	+	+	+
Lowe et al. 2016	+	+	−	+	−	+	+	+	+
Ludyga et al. 2018	+	+	−	−	−	+	+	+	+
Mehren et al. 2019	+	+	−	−	−	+	+	+	+
O'Leary et al. 2011	+	+	−	−	−	+	+	+	+
Oberste et al. 2016	+	+	−	+	+	+	+	+	+
Park & Etnier 2019	−	+	−	−	−	+	+	+	+
Peruyero et al. 2017	−	+	−	+	−	+	+	+	+
Pirrie and Lodewyk, 2012	−	+	−	−	−	+	+	+	+
Quintero et al., 2018	+	+	−	+	−	+	+	+	+
Sibley et al. 2006	+	+	−	−	−	+	+	+	+
Stroth et al. 2009	−	+	−	−	−	+	+	+	+
Van Regensburg et al. 2008	+	+	−	+	−	+	+	+	+
Wang et al. 2019	+	+	−	+	−	+	+	+	+
Weng et al. 2015	+	+	−	+	−	+	+	+	+
Yanagisawa et al. 2010	+	+	−	+	−	+	+	+	+

Figure 1 PEDro ratings for each item and each included study

Study	Acute Exercise Total	Acute Exercise Mean	Acute Exercise SD	Control Total	Control Mean	Control SD	SMD	95%-CI	Weight
Alves et al. 2014	22	18.67	1.21	22	20.94	1.16	−1.88	[−2.60; −1.16]	0.9%
Cooper et al. 2016	44	1043.00	37.00	44	1095.00	36.00	−1.41	[−1.88; −0.94]	1.6%
Chen et al. 2014	44	6.72	18.23	39	28.06	21.47	−1.07	[−1.53; −0.60]	1.6%
Kamijo et al. 2007 vigorous	13	383.24	12.09	6	414.90	48.96	−1.06	[−2.10; −0.02]	0.5%
Lowe et al. 2014 light	10	−17.26	318.24	6	315.51	328.13	−0.98	[−2.06; 0.11]	0.5%
Park et al. 2019	22	−63.36	8.92	22	−55.05	9.06	−0.91	[−1.53; −0.28]	1.1%
Chu et al. 2015 higher−fit	22	602.00	83.00	22	679.00	86.00	−0.89	[−1.52; −0.27]	1.1%
Endo et al. 2013 moderate	13	57.90	10.10	7	68.10	13.34	−0.87	[−1.83; 0.10]	0.6%
Kamijo et al. 2007 moderate	26	384.48	11.43	26	414.90	48.96	−0.84	[−1.41; −0.27]	1.3%
Lowe et al. 2014 moderate	12	60.61	293.36	6	315.51	328.13	−0.80	[−1.82; 0.23]	0.5%
Kamijo et al. 2009 young_adults moderate	12	387.80	12.40	6	397.40	13.50	−0.72	[−1.73; 0.30]	0.5%
Chang et al. 2017	30	463.42	52.31	30	509.30	78.38	−0.68	[−1.20; −0.16]	1.4%
Kao et al. 2018 HIIT	36	49.90	10.10	18	57.80	14.42	−0.67	[−1.25; −0.09]	1.2%
Kamijo et al. 2009 older_adults moderate	12	462.20	17.00	6	473.80	16.70	−0.65	[−1.66; 0.36]	0.6%
Endo et al. 2013 light	26	58.60	14.82	7	68.10	13.34	−0.64	[−1.49; 0.21]	0.7%
Finkenzeller et al. 2018	12	416.37	27.95	12	438.55	38.47	−0.64	[−1.46; 0.19]	0.8%
Byun et al. 2014	25	133.00	40.36	25	181.90	100.70	−0.63	[−1.20; −0.06]	1.3%
Best et al. 2012	33	34.20	70.66	33	91.20	110.87	−0.61	[−1.10; −0.11]	1.5%
Alves et al. 2012	42	16.79	4.25	42	19.60	5.11	−0.59	[−1.03; −0.15]	1.7%
Peruyero et al. 2017	88	−66.58	10.97	44	−60.45	8.76	−0.59	[−0.96; −0.22]	1.9%
Chang et al. 2015 20min	26	492.05	66.64	9	537.63	97.48	−0.59	[−1.36; 0.18]	0.8%
Ligeza et al. 2018 moderate	18	34.00	22.00	9	48.00	27.00	−0.57	[−1.39; 0.24]	0.8%
Chang et al. 2015a higher−fit	21	589.15	85.45	21	636.17	113.50	−0.46	[−1.07; 0.15]	1.1%
Chang et al. 2019 10min	40	629.28	64.20	13	659.88	75.12	−0.45	[−1.08; 0.18]	1.1%
Brown & Bray, 2018* moderate	21	529.72	66.00	5	561.85	85.08	−0.45	[−1.43; 0.54]	0.6%
Chu et al. 2015 average−fit	24	664.00	110.00	24	711.00	109.00	−0.42	[−0.99; 0.15]	1.3%
Wang et al. 2019	42	525.63	71.74	42	563.36	105.25	−0.42	[−0.85; 0.02]	1.7%
Hyodo et al. 2012	16	210.31	46.39	16	232.35	57.22	−0.41	[−1.11; 0.29]	1.0%
Kao et al. 2017 HIIT	64	384.80	45.40	32	402.60	54.20	−0.36	[−0.79; 0.06]	1.7%
O'Leary et al. 2011	36	43.90	15.60	36	49.00	13.80	−0.34	[−0.81; 0.12]	1.6%
Jäger et al. 2014	52	105.22	126.32	52	152.15	153.03	−0.33	[−0.72; 0.06]	1.9%
Basso et al. 2015* 30min delay	11	−15.50	5.97	10	−13.09	8.41	−0.32	[−1.18; 0.54]	0.7%
Hillman et al. 2009	20	55.00	22.81	20	64.10	32.65	−0.32	[−0.94; 0.31]	1.1%
Basso et al. 2015* 60min delay	11	−13.99	6.47	10	−11.37	9.46	−0.31	[−1.18; 0.55]	0.7%
Mehren et al. 2019 moderate	32	74.00	16.97	32	80.00	22.63	−0.30	[−0.79; 0.20]	1.5%
Chang et al. 2015 10min	26	512.54	82.86	9	537.63	97.48	−0.28	[−1.04; 0.48]	0.9%
Kao et al. 2018 moderate	36	53.20	17.40	18	57.80	14.42	−0.27	[−0.84; 0.29]	1.3%
Quintero et al. 2018	12	−45.09	9.12	8	−42.88	8.31	−0.24	[−1.14; 0.66]	0.7%
Crush & Loprinzi 2017 10min & 5min delay	22	1178.00	279.00	22	1243.00	264.00	−0.24	[−0.83; 0.36]	1.2%
Yanagisawa et al. 2010	20	78.04	268.75	20	156.65	432.05	−0.21	[−0.84; 0.41]	1.1%
Chang et al. 2015 45min	26	516.28	99.24	9	537.63	97.48	−0.21	[−0.97; 0.55]	0.9%
Ludyga et al. 2018	51	368.70	39.70	51	377.10	39.40	−0.21	[−0.60; 0.18]	1.9%
Kao et al. 2017 moderate	64	392.20	48.10	32	402.60	54.20	−0.21	[−0.63; 0.22]	1.7%
Sibley et al. 2006	86	19.24	3.44	86	19.98	3.99	−0.20	[−0.50; 0.10]	2.2%
Lowe et al. 2016	51	32.59	54.99	51	43.42	64.01	−0.18	[−0.57; 0.21]	1.9%
Barella et al. 2010	20	783.92	117.13	20	802.74	120.74	−0.16	[−0.78; 0.47]	1.1%
Chang et al. 2019 10min	40	649.26	70.64	13	659.88	75.12	−0.15	[−0.77; 0.48]	1.1%
Crush & Loprinzi 2017 60min & 5min delay	22	1142.00	320.00	22	1186.00	354.00	−0.13	[−0.72; 0.46]	1.2%
Chang et al. 2019 45min	40	651.06	66.70	13	659.88	75.12	−0.13	[−0.75; 0.50]	1.1%
Chang et al. 2015a average−fit	21	625.75	79.37	21	636.17	84.31	−0.12	[−0.73; 0.48]	1.2%
Douris et al. 2018	10	−58.30	9.40	10	−57.10	10.20	−0.12	[−0.99; 0.76]	0.7%
Mehren et al. 2019 HIIT	31	70.00	16.70	31	72.00	22.27	−0.10	[−0.60; 0.40]	1.5%
Crush & Loprinzi 2017 30min & 15min delay	22	1187.00	399.00	22	1226.00	381.00	−0.10	[−0.69; 0.49]	1.2%
Crush & Loprinzi 2017 20min & 15min delay	22	1131.00	312.00	22	1160.00	327.00	−0.09	[−0.68; 0.50]	1.2%
Crush & Loprinzi 2017 10min & 15min delay	22	1069.00	431.00	22	1099.00	315.00	−0.08	[−0.67; 0.51]	1.2%
Crush & Loprinzi 2017 45min & 5min delay	22	1201.00	418.00	22	1225.00	402.00	−0.06	[−0.65; 0.53]	1.2%
Abe et al. 2018 non−comparable_CG	13	181.80	79.40	26	186.20	97.80	−0.05	[−0.71; 0.62]	1.0%
Crush & Loprinzi 2017 20min & 30min delay	22	1226.00	373.00	22	1245.00	468.00	−0.04	[−0.64; 0.55]	1.2%
Crush & Loprinzi 2017 60min & 30min delay	22	1313.00	443.00	22	1330.00	455.00	−0.04	[−0.63; 0.55]	1.2%
Crush & Loprinzi 2017 45min & 30min delay	22	1184.00	474.00	22	1195.00	430.00	−0.02	[−0.61; 0.57]	1.2%
Stroth et al. 2009	33	310.90	38.13	17	311.44	36.37	−0.01	[−0.60; 0.57]	1.2%
Pirrie et al. 2012	40	41.33	6.27	40	41.47	13.10	−0.01	[−0.45; 0.42]	1.7%
Brown & Bray, 2018* vigorous	44	561.27	76.36	5	561.85	85.08	−0.01	[−0.93; 0.92]	0.6%
Crush & Loprinzi 2017 20min & 5min delay	22	1097.00	360.00	22	1092.00	239.00	0.02	[−0.57; 0.61]	1.2%
Oberste et al. 2016* light	30	84.03	50.70	10	82.90	74.17	0.02	[−0.70; 0.74]	0.9%
Crush & Loprinzi 2017 45min & 15min delay	22	1076.00	480.00	22	1068.00	258.00	0.02	[−0.57; 0.61]	1.2%
Crush & Loprinzi 2017 30min & 5min delay	22	1210.00	454.00	22	1198.00	493.00	0.02	[−0.57; 0.62]	1.2%
Crush & Loprinzi 2017 10min & 30min delay	22	1278.00	418.00	22	1260.00	541.00	0.04	[−0.55; 0.63]	1.2%
Gothe et al. 2013 comparable CG	15	421.41	53.28	30	419.03	60.46	0.04	[−0.58; 0.66]	1.1%
de Marco et al. 2014	47	65.15	73.20	47	61.98	75.91	0.04	[−0.36; 0.45]	1.8%
Brown & Bray, 2018* light	22	565.43	79.10	5	561.85	85.08	0.04	[−0.93; 1.01]	0.6%
Kamijo et al. 2009 young_adults light	12	398.00	12.20	6	397.40	13.50	0.05	[−0.93; 1.03]	0.6%
Hogan et al. 2013	30	309.26	38.89	30	307.06	32.23	0.06	[−0.45; 0.57]	1.4%
van Regensburg & Taylor 2008	23	671.20	240.10	23	656.30	151.70	0.07	[−0.51; 0.65]	1.2%
Brown & Bray, 2018* HIIT	44	569.16	54.56	5	561.85	85.08	0.12	[−0.80; 1.05]	0.6%
Weng et al. 2015	25	510.43	59.30	25	503.43	47.00	0.13	[−0.43; 0.68]	1.3%
Hwang et al. 2016	29	−0.68	0.07	29	−0.69	0.08	0.13	[−0.38; 0.65]	1.4%
Gothe et al. 2013 non−comparable_CG	15	421.41	53.28	30	413.19	53.87	0.15	[−0.47; 0.77]	1.1%
Hillman et al. 2003*	19	16.10	12.34	19	13.97	13.10	0.16	[−0.47; 0.80]	1.1%
Ligeza et al. 2018 HIIT	18	52.00	20.00	9	48.00	27.00	0.17	[−0.63; 0.97]	0.8%
Egger et al. 2018	50	154.13	153.53	54	121.30	138.67	0.22	[−0.16; 0.61]	1.9%
Oberste et al. 2016* vigorous	30	95.57	46.67	10	82.90	74.17	0.23	[−0.49; 0.95]	0.9%
Abe et al. 2018 comparable_CG	13	181.80	79.40	26	157.10	105.00	0.25	[−0.42; 0.92]	1.0%
Crush & Loprinzi 2017 30min & 30min delay	22	1190.00	354.00	22	1102.00	322.00	0.26	[−0.34; 0.85]	1.2%
Crush & Loprinzi 2017 60min & 15min delay	22	1166.00	412.00	22	1071.00	232.00	0.28	[−0.32; 0.87]	1.2%
Oberste et al. 2016* moderate	30	109.30	75.44	10	82.90	74.17	0.34	[−0.38; 1.06]	0.9%
Kamijo et al. 2009 older_adults light	12	480.90	20.40	6	473.80	16.70	0.35	[−0.64; 1.34]	0.6%
**Random effects model**	**2436**			**1938**			**−0.26**	**[−0.34; −0.18]**	**100.0%**
**Prediction interval**								**[−0.74; 0.22]**	

Heterogeneity: $I^2 = 39\%$, $\tau^2 = 0.0557$, $p < 0.01$

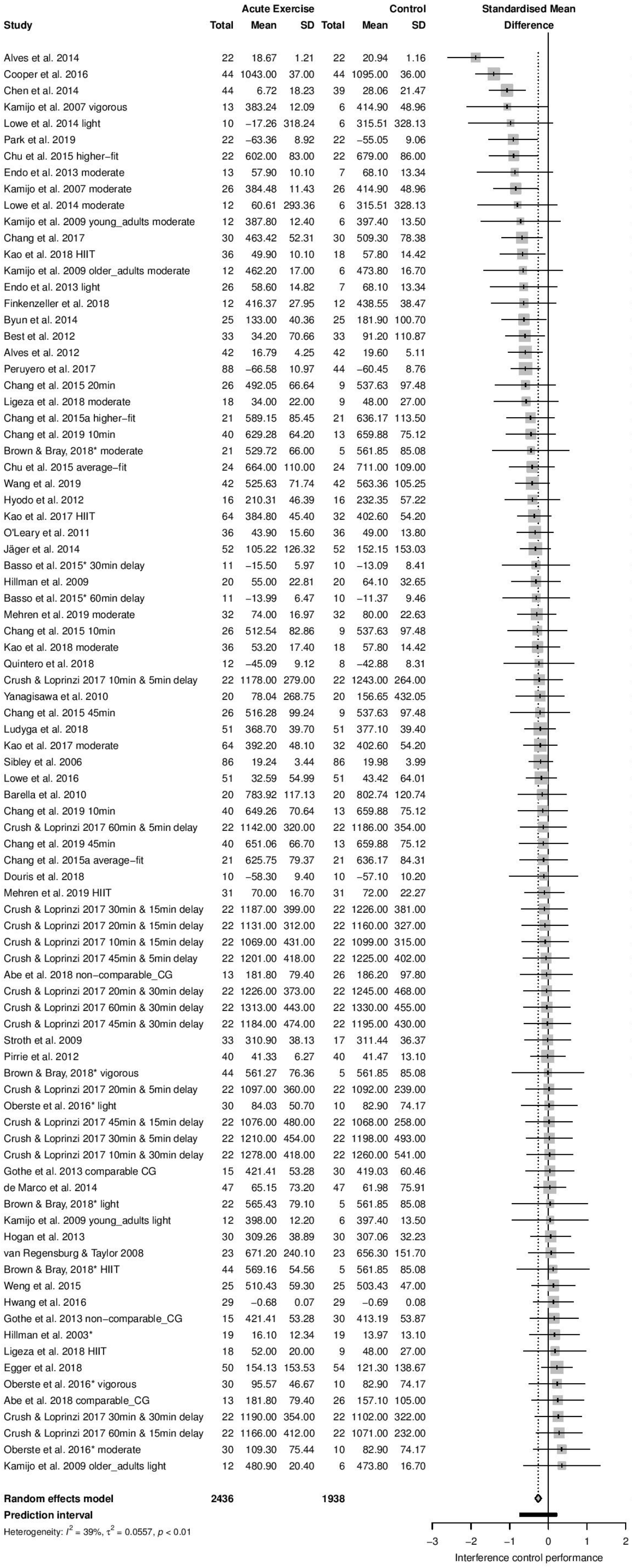

Figure 2 **Forest plot to primary analysis of time-dependent measures of interference control**

Study	Acute Exercise Total	Acute Exercise Mean	Acute Exercise SD	Control Total	Control Mean	Control SD	Standardised Mean Difference	SMD	95%-CI	Weight
Finkenzeller et al., 2018	12	−4.83	2.89	12	−3.08	2.19		−0.66	[−1.48; 0.17]	1.2%
Gothe et al. 2013 comparable CG	15	83.37	12.27	30	90.27	10.63		−0.61	[−1.24; 0.03]	2.0%
Oberste et al., 2016* moderate	30	−1.22	2.77	10	−0.45	1.89		−0.29	[−1.01; 0.43]	1.6%
Byun et al. 2014	25	93.60	6.50	25	95.60	8.00		−0.27	[−0.83; 0.29]	2.6%
Hillman et al., 2003*	19	91.55	6.97	19	92.97	4.68		−0.23	[−0.87; 0.40]	2.0%
Kamijo et al., 2009 young_adults moderate	12	94.90	7.97	6	96.30	2.77		−0.20	[−1.18; 0.79]	0.8%
Kamijo et al., 2009 young_adults light	12	95.20	6.24	6	96.30	2.77		−0.19	[−1.18; 0.79]	0.8%
Hogan et al., 2013	30	−22.39	6.39	30	−21.45	5.16		−0.16	[−0.67; 0.35]	3.2%
Brown & Bray, 2018* vigorous	22	91.79	7.35	5	92.92	3.87		−0.16	[−1.13; 0.81]	0.9%
Kamijo et al., 2007 moderate	24	−17.45	7.00	6	−16.42	7.23		−0.14	[−1.04; 0.75]	1.0%
Chang et al. 2015 45min	26	85.61	10.32	9	86.79	9.12		−0.11	[−0.87; 0.64]	1.4%
Ligeza et al., 2018 HIIT	18	−10.00	11.50	18	−9.00	9.00		−0.09	[−0.75; 0.56]	1.9%
Gothe et al. 2013 non−comparable_CG	15	83.37	12.27	30	84.37	12.61		−0.08	[−0.70; 0.54]	2.1%
Kamijo et al., 2009 older_adults moderate	12	94.70	4.50	6	94.80	4.16		−0.02	[−1.00; 0.96]	0.9%
Oberste et al., 2016* vigorous	30	−0.49	1.99	10	−0.45	1.89		−0.02	[−0.74; 0.70]	1.6%
Drolette et al., 2012	36	94.40	0.80	36	94.40	1.20		0.00	[−0.46; 0.46]	3.8%
Kamijo et al., 2007 vigorous	12	−16.34	6.28	6	−16.42	7.23		0.01	[−0.97; 0.99]	0.9%
Kao et al., 2018 HIIT	36	−5.91	5.34	18	−5.99	6.66		0.01	[−0.55; 0.58]	2.6%
Kao et al., 2017 moderate	64	91.30	6.90	32	91.10	7.20		0.03	[−0.40; 0.45]	4.5%
Kao et al., 2018 moderate	36	−5.47	6.23	18	−5.99	6.66		0.08	[−0.49; 0.65]	2.6%
Brown & Bray, 2018* moderate	21	93.52	6.17	5	92.92	3.87		0.10	[−0.88; 1.08]	0.9%
Oberste et al., 2016* light	30	−0.26	1.80	10	−0.45	1.89		0.10	[−0.61; 0.82]	1.6%
Chang et al., 2019 10/20 min	40	94.05	7.37	13	93.10	7.70		0.13	[−0.50; 0.75]	2.1%
Cooper et al., 2016	44	94.10	10.30	44	93.00	6.40		0.13	[−0.29; 0.55]	4.7%
Chang et al., 2019 10/20 min	40	94.29	8.63	13	93.10	7.70		0.14	[−0.49; 0.77]	2.1%
Brown & Bray, 2018* HIIT	22	93.94	5.60	5	92.92	3.87		0.18	[−0.79; 1.16]	0.9%
O'Leary et al., 2011	36	89.60	7.60	36	88.10	8.40		0.19	[−0.28; 0.65]	3.8%
Hyodo et al., 2012	16	−4.81	6.22	16	−6.10	5.92		0.21	[−0.49; 0.90]	1.7%
Chu et al., 2015 higher−fit	22	84.00	21.00	22	80.00	15.00		0.22	[−0.38; 0.81]	2.3%
Weng et al., 2015	25	93.00	5.11	25	91.60	6.70		0.23	[−0.33; 0.79]	2.6%
Chang et al., 2019 45 min	40	94.71	6.53	13	93.10	7.70		0.23	[−0.39; 0.86]	2.1%
Alves et al., 2012	42	−0.61	1.03	42	−1.01	2.07		0.24	[−0.19; 0.67]	4.4%
Brown & Bray, 2018* light	22	94.32	5.78	5	92.92	3.87		0.25	[−0.73; 1.22]	0.9%
Ludyga et al., 2018	51	93.00	7.00	51	91.00	7.00		0.28	[−0.11; 0.67]	5.4%
Lowe et al., 2016	51	98.60	1.30	51	96.60	9.60		0.29	[−0.10; 0.68]	5.4%
Chen et al., 2014	44	93.86	0.25	39	93.77	0.36		0.29	[−0.14; 0.72]	4.4%
Kamijo et al., 2009 older_adults light	12	96.10	3.81	6	94.80	4.16		0.32	[−0.67; 1.30]	0.8%
Chang et al. 2015 10min	26	89.13	6.31	9	86.79	9.12		0.32	[−0.44; 1.08]	1.4%
Kao et al., 2017 HIIT	64	93.20	5.80	32	91.10	7.20		0.33	[−0.10; 0.76]	4.5%
Ligeza et al., 2018 moderate	18	−6.00	5.00	18	−9.00	9.00		0.40	[−0.26; 1.06]	1.9%
Hillman et al., 2009	20	−6.60	4.47	20	−10.30	8.50		0.53	[−0.10; 1.17]	2.1%
Yanagisawa et al., 2010	20	−7.00	1.50	20	−8.00	1.40		0.68	[0.04; 1.31]	2.0%
Chang et al. 2015 20min	26	92.60	6.46	9	86.79	9.12		0.79	[0.01; 1.57]	1.3%
Chu et al., 2015 lower−fit	24	93.00	6.00	24	86.00	9.00		0.90	[0.30; 1.50]	2.3%
**Random effects model**	**1242**			**860**				**0.13**	**[0.04; 0.22]**	**100.0%**
**Prediction interval**									**[0.04; 0.23]**	

Heterogeneity: $I^2 = 0\%$, $\tau^2 = 0$, $p = 0.76$

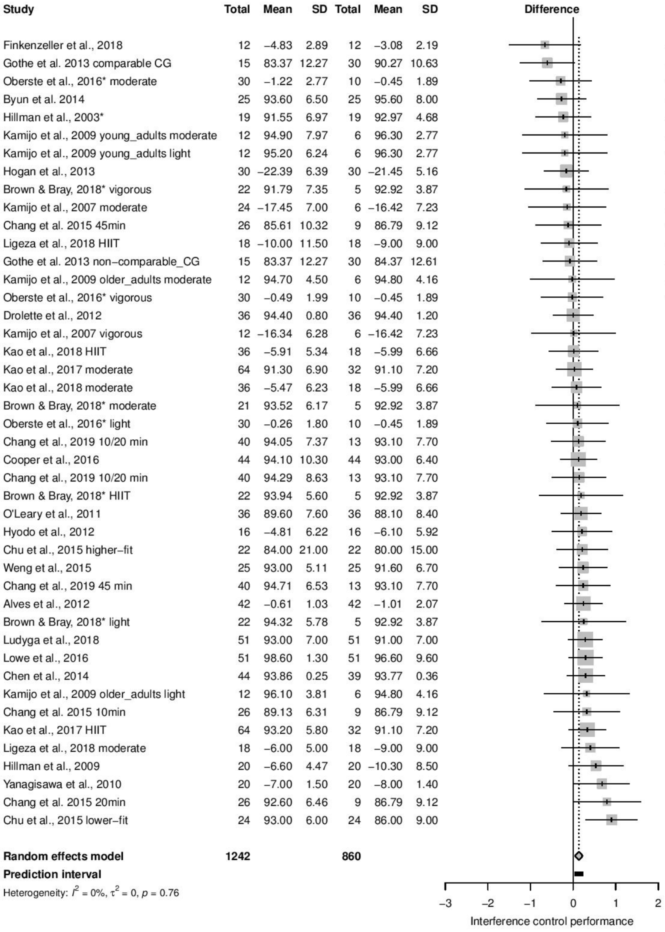

Figure 3 **Forest plot to primary analysis of accuracy measures of interference control**

Study	Acute Exercise Total	Acute Exercise Mean	Acute Exercise SD	Control Total	Control Mean	Control SD	SMD	95%-CI	Weight
**HIIT**									
Ligeza et al., 2018 HIIT	18	−10.00	11.50	18	−9.00	9.00	−0.09	[−0.75; 0.56]	1.9%
Kao et al., 2018 HIIT	36	−5.91	5.34	18	−5.99	6.66	0.01	[−0.55; 0.58]	2.6%
Cooper et al., 2016	44	94.10	10.30	44	93.00	6.40	0.13	[−0.29; 0.55]	4.7%
Brown & Bray, 2018* HIIT	22	93.94	5.60	5	92.92	3.87	0.18	[−0.79; 1.16]	0.9%
Kao et al., 2017 HIIT	64	93.20	5.80	32	91.10	7.20	0.33	[−0.10; 0.76]	4.5%
Random effects model	184			117			0.14	[−0.09; 0.38]	14.7%
Heterogeneity: $I^2 = 0\%$, $\tau^2 = 0$, $p = 0.83$									
**vigorous**									
Hillman et al., 2003*	19	91.55	6.97	19	92.97	4.68	−0.23	[−0.87; 0.40]	2.0%
Brown & Bray, 2018* vigorous	22	91.79	7.35	5	92.92	3.87	−0.16	[−1.13; 0.81]	0.9%
Chang et al. 2015 45min	26	85.61	10.32	9	86.79	9.12	−0.11	[−0.87; 0.64]	1.4%
Oberste et al., 2016* vigorous	30	−0.49	1.99	10	−0.45	1.89	−0.02	[−0.74; 0.70]	1.6%
Kamijo et al., 2007 vigorous	12	−16.34	6.28	6	−16.42	7.23	0.01	[−0.97; 0.99]	0.9%
Chang et al., 2019 10/20 min	40	94.05	7.37	13	93.10	7.70	0.13	[−0.50; 0.75]	2.1%
Chang et al., 2019 10/20 min	40	94.29	8.63	13	93.10	7.70	0.14	[−0.49; 0.77]	2.1%
Chu et al., 2015 higher−fit	22	84.00	21.00	22	80.00	15.00	0.22	[−0.38; 0.81]	2.4%
Chang et al., 2019 45 min	40	94.71	6.53	13	93.10	7.70	0.23	[−0.39; 0.86]	2.1%
Chang et al. 2015 10min	26	89.13	6.31	9	86.79	9.12	0.32	[−0.44; 1.08]	1.4%
Chang et al. 2015 20min	26	92.60	6.46	9	86.79	9.12	0.79	[0.01; 1.57]	1.4%
Chu et al., 2015 lower−fit	24	93.00	6.00	24	86.00	9.00	0.90	[0.30; 1.50]	2.3%
Random effects model	327			152			0.21	[0.01; 0.41]	20.6%
Heterogeneity: $I^2 = 1\%$, $\tau^2 = 0$, $p = 0.43$									
**moderate**									
Gothe et al. 2013 comparable CG	15	83.37	12.27	30	90.27	10.63	−0.61	[−1.24; 0.03]	2.1%
Oberste et al., 2016* moderate	30	−1.22	2.77	10	−0.45	1.89	−0.29	[−1.01; 0.43]	1.6%
Kamijo et al., 2009 young_adults moderate	12	94.90	7.97	6	96.30	2.77	−0.20	[−1.18; 0.79]	0.9%
Hogan et al., 2013	30	−22.39	6.39	30	−21.45	5.16	−0.16	[−0.67; 0.35]	3.2%
Kamijo et al., 2007 moderate	24	−17.45	7.00	6	−16.42	7.23	−0.14	[−1.04; 0.75]	1.0%
Gothe et al. 2013 non−comparable_CG	15	83.37	12.27	30	84.37	12.61	−0.08	[−0.70; 0.54]	2.2%
Kamijo et al., 2009 older_adults moderate	12	94.70	4.50	6	94.80	4.16	−0.02	[−1.00; 0.96]	0.9%
Drolette et al., 2012	36	94.40	0.80	36	94.40	1.20	0.00	[−0.46; 0.46]	3.9%
Kao et al., 2017 moderate	64	91.30	6.90	32	91.10	7.20	0.03	[−0.40; 0.45]	4.6%
Kao et al., 2018 moderate	36	−5.47	6.23	18	−5.99	6.66	0.08	[−0.49; 0.65]	2.6%
Brown & Bray, 2018* moderate	21	93.52	6.17	5	92.92	3.87	0.10	[−0.88; 1.08]	0.9%
O'Leary et al., 2011	36	89.60	7.60	36	88.10	8.40	0.19	[−0.28; 0.65]	3.9%
Hyodo et al., 2012	16	−4.81	6.22	16	−6.10	5.92	0.21	[−0.49; 0.90]	1.7%
Weng et al., 2015	25	93.00	5.11	25	91.60	6.70	0.23	[−0.33; 0.79]	2.7%
Alves et al., 2012	42	−0.61	1.03	42	−1.01	2.07	0.24	[−0.19; 0.67]	4.5%
Ludyga et al., 2018	51	93.00	7.00	51	91.00	7.00	0.28	[−0.11; 0.67]	5.4%
Lowe et al., 2016	51	98.60	1.30	51	96.60	9.60	0.29	[−0.10; 0.68]	5.4%
Chen et al., 2014	44	93.86	0.25	39	93.77	0.36	0.29	[−0.14; 0.72]	4.4%
Ligeza et al., 2018 moderate	18	−6.00	5.00	18	−9.00	9.00	0.40	[−0.26; 1.06]	1.9%
Hillman et al., 2009	20	−6.60	4.47	20	−10.30	8.50	0.53	[−0.10; 1.17]	2.1%
Yanagisawa et al., 2010	20	−7.00	1.50	20	−8.00	1.40	0.68	[0.04; 1.31]	2.0%
Random effects model	618			527			0.14	[0.02; 0.26]	57.8%
Heterogeneity: $I^2 = 0\%$, $\tau^2 = 0$, $p = 0.66$									
**light**									
Byun et al. 2014	25	93.60	6.50	25	95.60	8.00	−0.27	[−0.83; 0.29]	2.7%
Kamijo et al., 2009 young_adults light	12	95.20	6.24	6	96.30	2.77	−0.19	[−1.18; 0.79]	0.9%
Oberste et al., 2016* light	30	−0.26	1.80	10	−0.45	1.89	0.10	[−0.61; 0.82]	1.6%
Brown & Bray, 2018* light	22	94.32	5.78	5	92.92	3.87	0.25	[−0.73; 1.22]	0.9%
Kamijo et al., 2009 older_adults light	12	96.10	3.81	6	94.80	4.16	0.32	[−0.67; 1.30]	0.9%
Random effects model	101			52			−0.03	[−0.38; 0.31]	6.9%
Heterogeneity: $I^2 = 0\%$, $\tau^2 = 0$, $p = 0.78$									
**Random effects model**	**1230**			**848**			**0.14**	**[0.05; 0.23]**	**100.0%**
Heterogeneity: $I^2 = 0\%$, $\tau^2 = 0$, $p = 0.85$									

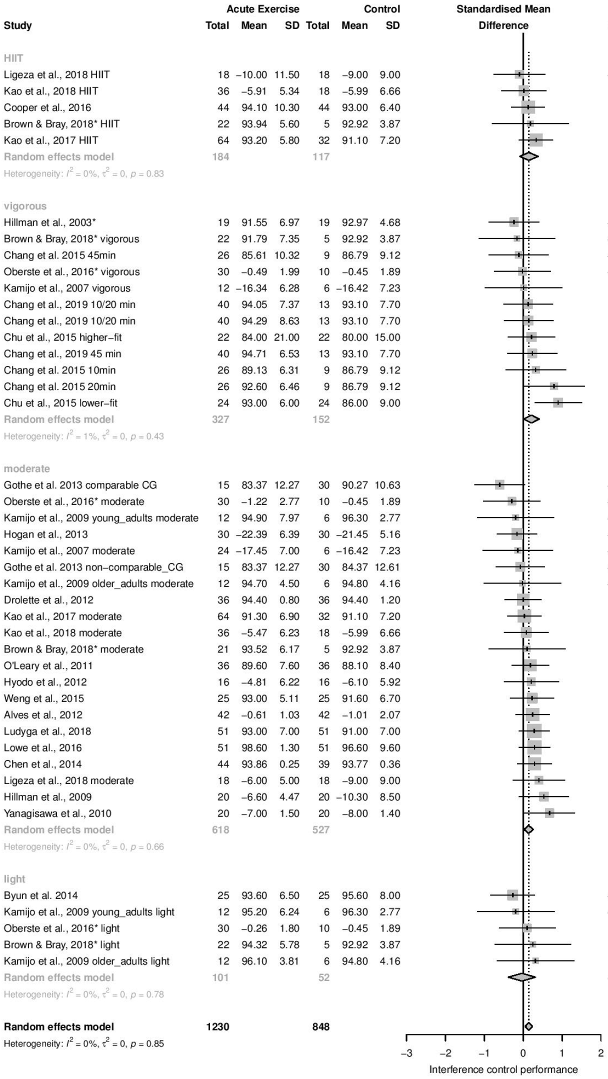

Figure 4 Forest plot to moderator analysis of exercise intensity (accuracy measures of interference control)

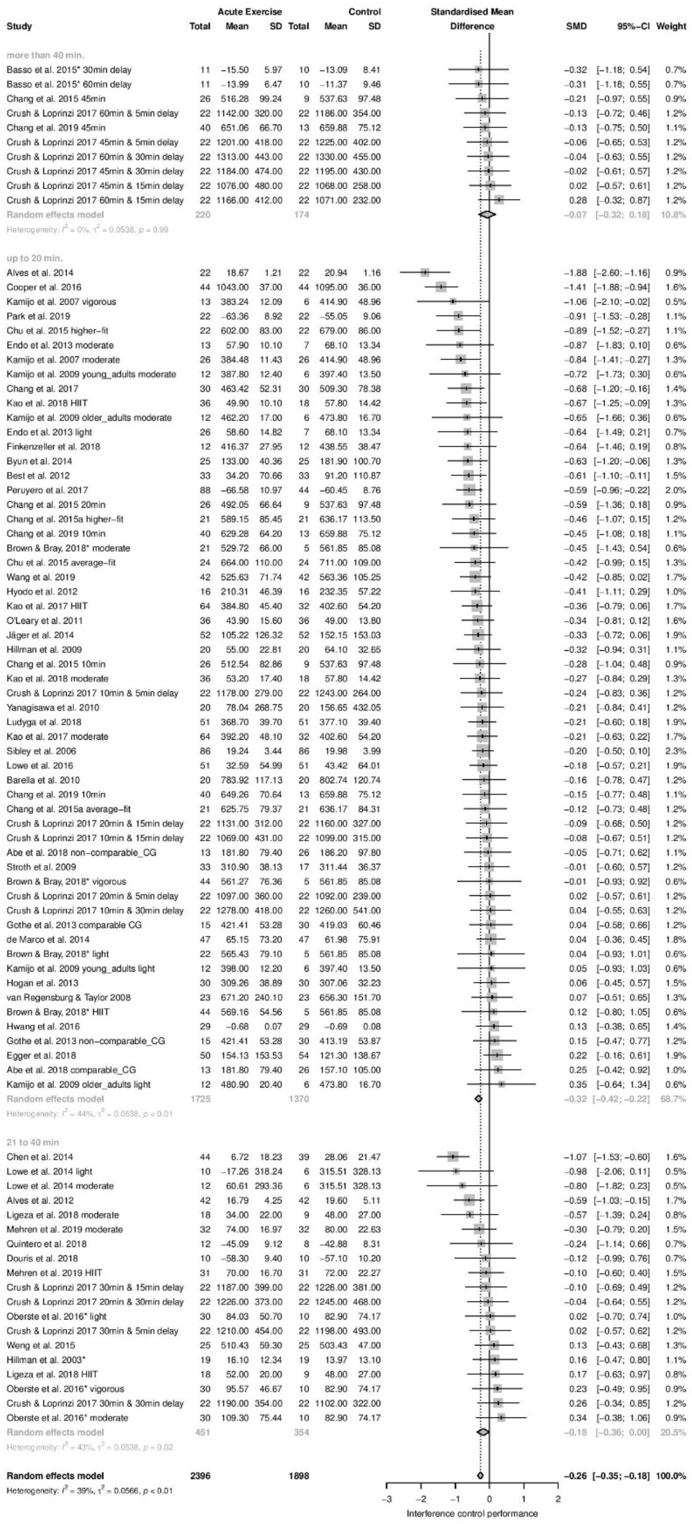

Figure 5 **Subgroup analysis for exercise duration (time-dependent measures of interference control)**

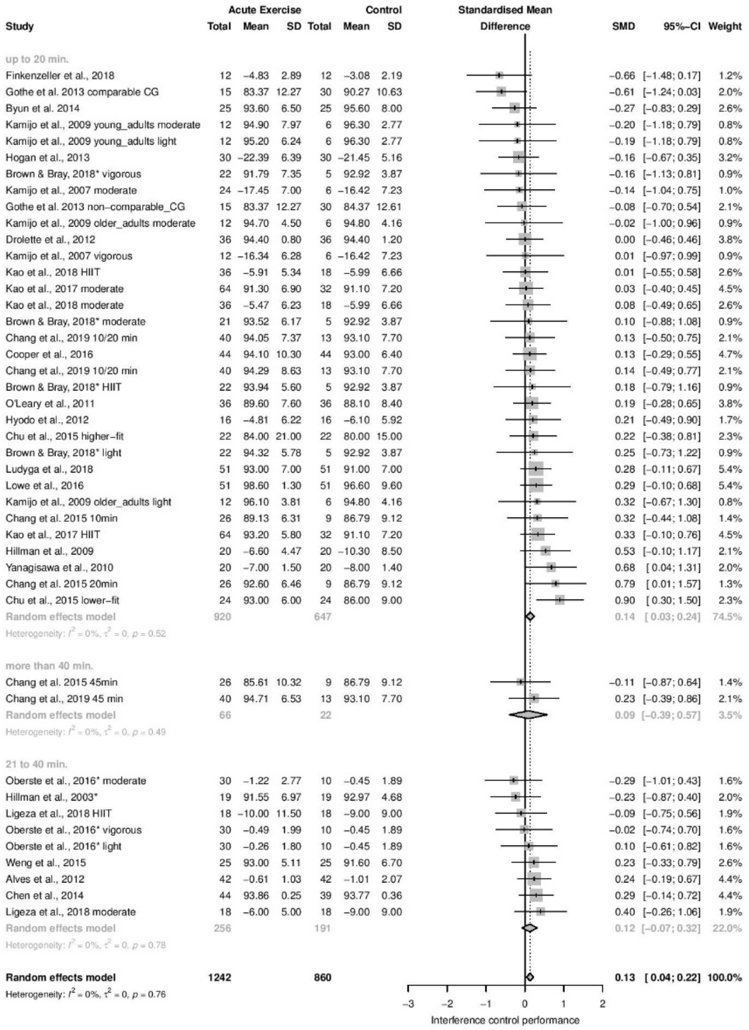

Figure 6 **Subgroup analysis for exercise duration (accuracy measures of interference control)**

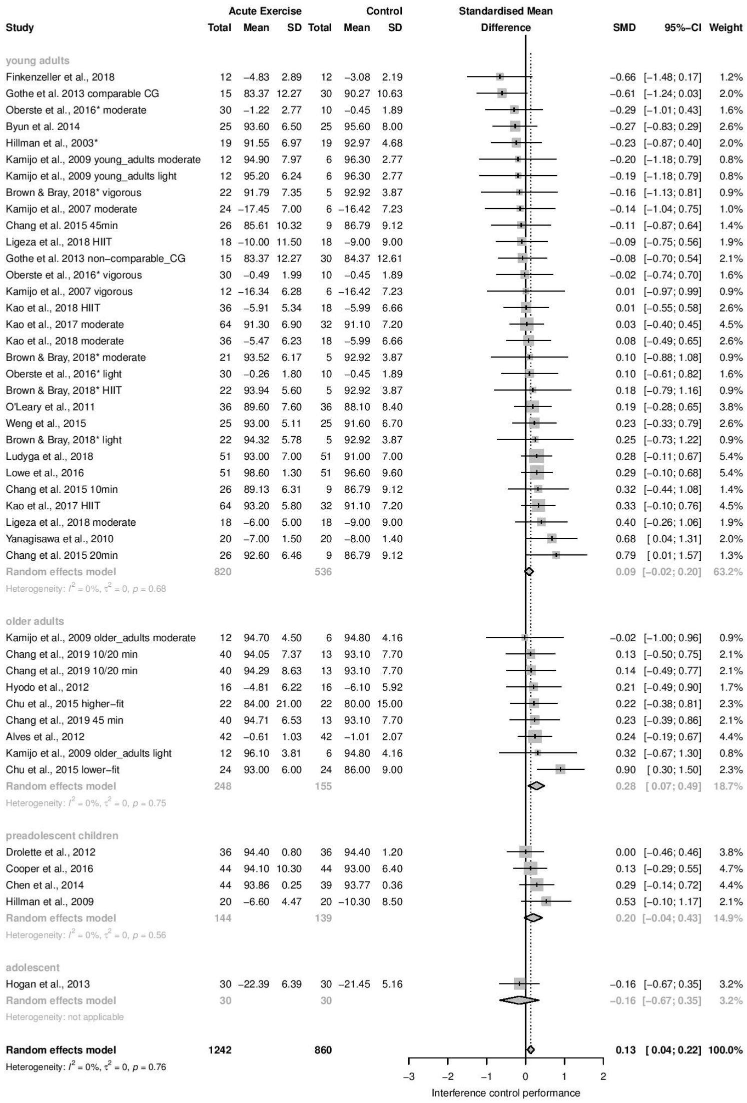

Figure 7 **Subgroup analysis for age groups (accuracy measures of interference control)**

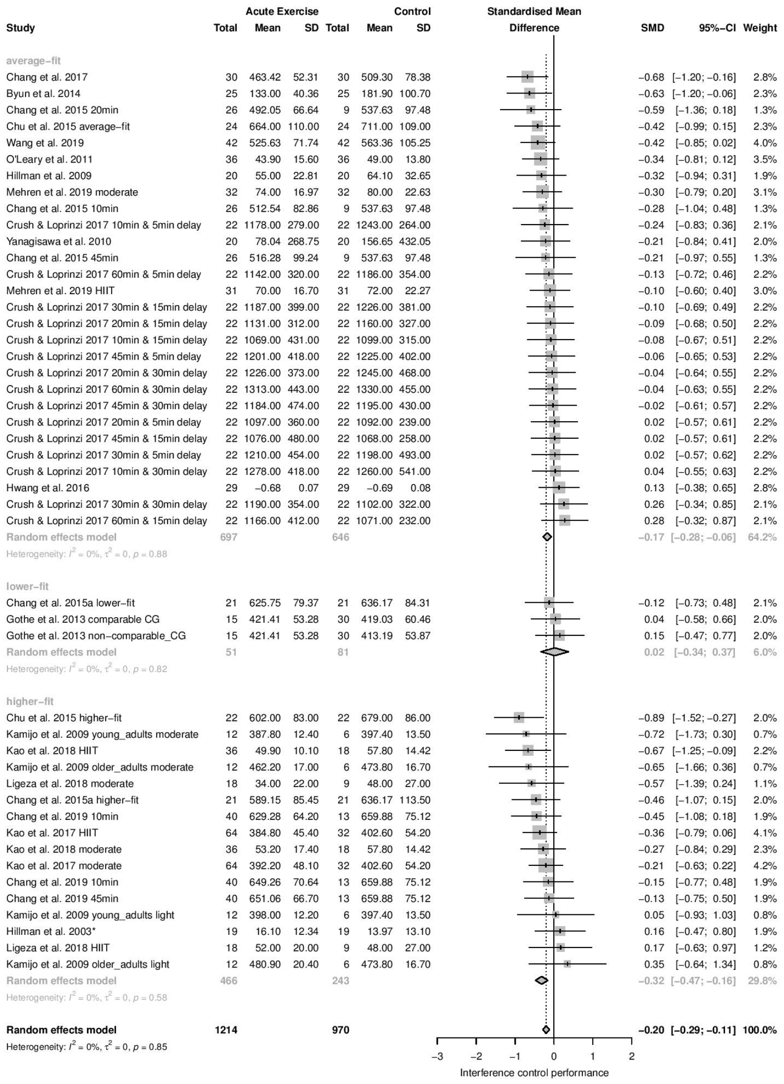

Figure 8 **Subgroup analysis of aerobic fitness level (time-dependent measures)**

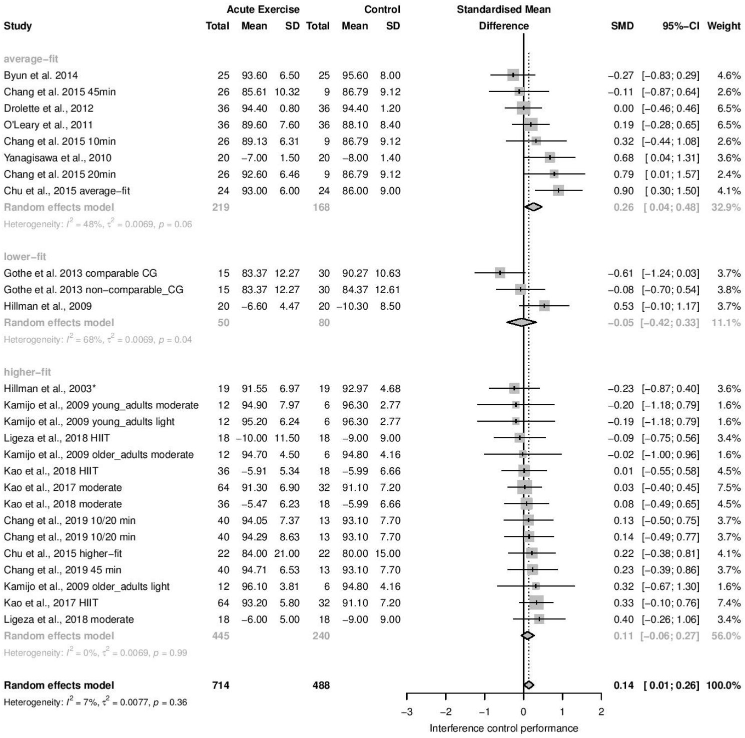

Figure 9 **Subgroup analysis for aerobic fitness level (accuracy measures of interference control)**

Study	Acute Exercise Total	Acute Exercise Mean	Acute Exercise SD	Control Total	Control Mean	Control SD	SMD	95%−CI	Weight
**not_comparable**									
Cooper et al. 2016	44	1043.00	37.00	44	1095.00	36.00	−1.41	[−1.88; −0.94]	1.6%
Chen et al. 2014	44	6.72	18.23	39	28.06	21.47	−1.07	[−1.53; −0.60]	1.6%
Kamijo et al. 2007 vigorous	13	383.24	12.09	6	414.90	48.96	−1.06	[−2.10; −0.02]	0.5%
Park et al. 2019	22	−63.36	8.92	22	−55.05	9.06	−0.91	[−1.53; −0.28]	1.1%
Chu et al. 2015 higher−fit	22	602.00	83.00	22	679.00	86.00	−0.89	[−1.52; −0.27]	1.1%
Endo et al. 2013 moderate	13	57.90	10.10	7	68.10	13.34	−0.87	[−1.83; 0.10]	0.6%
Kamijo et al. 2007 moderate	26	384.48	11.43	26	414.90	48.96	−0.84	[−1.41; −0.27]	1.3%
Kamijo et al. 2009 young_adults moderate	12	387.80	12.40	6	397.40	13.50	−0.72	[−1.73; 0.30]	0.5%
Chang et al. 2017	30	463.42	52.31	30	509.30	78.38	−0.68	[−1.20; −0.16]	1.4%
Kao et al. 2018 HIIT	36	49.90	10.10	18	57.80	14.42	−0.67	[−1.25; −0.09]	1.2%
Kamijo et al. 2009 older_adults moderate	12	462.20	17.00	6	473.80	16.70	−0.65	[−1.66; 0.36]	0.6%
Endo et al. 2013 light	26	58.60	14.82	7	68.10	13.34	−0.64	[−1.49; 0.21]	0.7%
Finkenzeller et al. 2018	12	416.37	27.95	12	438.55	38.47	−0.64	[−1.46; 0.19]	0.8%
Byun et al. 2014	25	133.00	40.36	25	181.90	100.70	−0.63	[−1.20; −0.06]	1.3%
Best et al. 2012	33	34.20	70.66	33	91.20	110.87	−0.61	[−1.10; −0.11]	1.5%
Peruyero et al. 2017	88	−66.58	10.97	44	−60.45	8.76	−0.59	[−0.96; −0.22]	1.9%
Chang et al. 2015 20min	26	492.05	66.64	9	537.63	97.48	−0.59	[−1.36; 0.18]	0.8%
Ligeza et al. 2018 moderate	18	34.00	22.00	9	48.00	27.00	−0.57	[−1.39; 0.24]	0.8%
Chang et al. 2015a higher−fit	21	589.15	85.45	21	636.17	113.50	−0.46	[−1.07; 0.15]	1.1%
Chang et al. 2019 10min	40	629.28	64.20	13	659.88	75.12	−0.45	[−1.08; 0.18]	1.1%
Brown & Bray, 2018* moderate	21	529.72	66.00	5	561.85	85.08	−0.45	[−1.43; 0.54]	0.6%
Chu et al. 2015 average−fit	24	664.00	110.00	24	711.00	109.00	−0.42	[−0.99; 0.15]	1.3%
Wang et al. 2019	42	525.63	71.74	42	563.36	105.25	−0.42	[−0.85; 0.02]	1.7%
Hyodo et al. 2012	16	210.31	46.39	16	232.35	57.22	−0.41	[−1.11; 0.29]	1.0%
Kao et al. 2017 HIIT	64	384.80	45.40	32	402.60	54.20	−0.36	[−0.79; 0.06]	1.7%
O'Leary et al. 2011	36	43.90	15.60	36	49.00	13.80	−0.34	[−0.81; 0.12]	1.6%
Jäger et al. 2014	52	105.22	126.32	52	152.15	153.03	−0.33	[−0.72; 0.06]	1.9%
Basso et al. 2015* 30min delay	11	−15.50	5.97	10	−13.09	8.41	−0.32	[−1.18; 0.54]	0.7%
Hillman et al. 2009	20	55.00	22.81	20	64.10	32.65	−0.32	[−0.94; 0.31]	1.1%
Basso et al. 2015* 60min delay	11	−13.99	6.47	10	−11.37	9.46	−0.31	[−1.18; 0.55]	0.7%
Mehren et al. 2019 moderate	32	74.00	16.97	32	80.00	22.63	−0.30	[−0.79; 0.20]	1.5%
Chang et al. 2015 10min	26	512.54	82.86	9	537.63	97.48	−0.28	[−1.04; 0.48]	0.9%
Kao et al. 2018 moderate	36	53.20	17.40	18	57.80	14.42	−0.27	[−0.84; 0.29]	1.3%
Quintero et al. 2018	12	−45.09	9.12	8	−42.88	8.31	−0.24	[−1.14; 0.66]	0.7%
Crush & Loprinzi 2017 10min & 5min delay	22	1178.00	279.00	22	1243.00	264.00	−0.24	[−0.83; 0.36]	1.2%
Yanagisawa et al. 2010	20	78.04	268.75	20	156.65	432.05	−0.21	[−0.84; 0.41]	1.1%
Chang et al. 2015 45min	26	516.28	99.24	9	537.63	97.48	−0.21	[−0.97; 0.55]	0.9%
Ludyga et al. 2018	51	368.70	39.70	51	377.10	39.40	−0.21	[−0.60; 0.18]	1.9%
Kao et al. 2017 moderate	64	392.20	48.10	32	402.60	54.20	−0.21	[−0.63; 0.22]	1.7%
Sibley et al. 2006	86	19.24	3.44	86	19.98	3.99	−0.20	[−0.50; 0.10]	2.2%
Barella et al. 2010	20	783.92	117.13	20	802.74	120.74	−0.16	[−0.78; 0.47]	1.1%
Chang et al. 2019 10min	40	649.26	70.64	13	659.88	75.12	−0.15	[−0.77; 0.48]	1.1%
Crush & Loprinzi 2017 60min & 5min delay	22	1142.00	320.00	22	1186.00	354.00	−0.13	[−0.72; 0.46]	1.2%
Chang et al. 2019 45min	40	651.06	66.70	13	659.88	75.12	−0.13	[−0.75; 0.50]	1.1%
Chang et al. 2015a average−fit	21	625.75	79.37	21	636.17	84.31	−0.12	[−0.73; 0.48]	1.2%
Douris et al. 2018	10	−58.30	9.40	10	−57.10	10.20	−0.12	[−0.99; 0.76]	0.7%
Mehren et al. 2019 HIIT	31	70.00	16.70	31	72.00	22.27	−0.10	[−0.60; 0.40]	1.5%
Crush & Loprinzi 2017 30min & 15min delay	22	1187.00	399.00	22	1226.00	381.00	−0.10	[−0.69; 0.49]	1.2%
Crush & Loprinzi 2017 20min & 15min delay	22	1131.00	312.00	22	1160.00	327.00	−0.09	[−0.68; 0.50]	1.2%
Crush & Loprinzi 2017 10min & 15min delay	22	1069.00	431.00	22	1099.00	315.00	−0.08	[−0.67; 0.51]	1.2%
Crush & Loprinzi 2017 45min & 5min delay	22	1201.00	418.00	22	1225.00	402.00	−0.06	[−0.65; 0.53]	1.2%
Abe et al. 2018 non−comparable_CG	13	181.80	79.40	26	186.20	97.80	−0.05	[−0.71; 0.62]	1.0%
Crush & Loprinzi 2017 20min & 30min delay	22	1226.00	373.00	22	1245.00	468.00	−0.04	[−0.64; 0.55]	1.2%
Crush & Loprinzi 2017 60min & 30min delay	22	1313.00	443.00	22	1330.00	455.00	−0.04	[−0.63; 0.55]	1.2%
Crush & Loprinzi 2017 45min & 30min delay	22	1184.00	474.00	22	1195.00	430.00	−0.02	[−0.61; 0.57]	1.2%
Stroth et al. 2009	33	310.90	38.13	17	311.44	36.37	−0.01	[−0.60; 0.57]	1.2%
Pirrie et al. 2012	40	41.33	6.27	40	41.47	13.10	−0.01	[−0.45; 0.42]	1.7%
Brown & Bray, 2018* vigorous	44	561.27	76.36	5	561.85	85.08	−0.01	[−0.93; 0.92]	0.6%
Crush & Loprinzi 2017 20min & 5min delay	22	1097.00	360.00	22	1092.00	239.00	0.02	[−0.57; 0.61]	1.2%
Crush & Loprinzi 2017 45min & 15min delay	22	1076.00	480.00	22	1068.00	258.00	0.02	[−0.57; 0.61]	1.2%
Crush & Loprinzi 2017 30min & 5min delay	22	1210.00	454.00	22	1198.00	493.00	0.02	[−0.57; 0.62]	1.2%
Crush & Loprinzi 2017 10min & 30min delay	22	1278.00	418.00	22	1260.00	541.00	0.04	[−0.55; 0.63]	1.2%
Brown & Bray, 2018* light	22	565.43	79.10	5	561.85	85.08	0.04	[−0.93; 1.01]	0.6%
Kamijo et al. 2009 young_adults light	12	398.00	12.20	6	397.40	13.50	0.05	[−0.93; 1.03]	0.6%
Hogan et al. 2013	30	309.26	38.89	30	307.06	32.23	0.06	[−0.45; 0.57]	1.4%
van Regensburg & Taylor 2008	23	671.20	240.10	23	656.30	151.70	0.07	[−0.51; 0.65]	1.2%
Brown & Bray, 2018* HIIT	44	569.16	54.56	5	561.85	85.08	0.12	[−0.80; 1.05]	0.6%
Hwang et al. 2016	29	−0.68	0.07	29	−0.69	0.08	0.13	[−0.38; 0.65]	1.4%
Gothe et al. 2013 non−comparable_CG	15	421.41	53.28	30	413.19	53.87	0.15	[−0.47; 0.77]	1.1%
Hillman et al. 2003*	19	16.10	12.34	19	13.97	13.10	0.16	[−0.47; 0.80]	1.1%
Ligeza et al. 2018 HIIT	18	52.00	20.00	9	48.00	27.00	0.17	[−0.63; 0.97]	0.8%
Egger et al. 2018	50	154.13	153.53	54	121.30	138.67	0.22	[−0.16; 0.61]	1.9%
Crush & Loprinzi 2017 30min & 30min delay	22	1190.00	354.00	22	1102.00	322.00	0.26	[−0.34; 0.85]	1.2%
Crush & Loprinzi 2017 60min & 15min delay	22	1166.00	412.00	22	1071.00	232.00	0.28	[−0.32; 0.87]	1.2%
Kamijo et al. 2009 older_adults light	12	480.90	20.40	6	473.80	16.70	0.35	[−0.64; 1.34]	0.6%
Random effects model	2109			1653			−0.27	[−0.36; −0.18]	86.5%
Heterogeneity: $I^2 = 29\%$, $\tau^2 = 0.0570$, $p = 0.01$									
**comparable**									
Alves et al. 2014	22	18.67	1.21	22	20.94	1.16	−1.88	[−2.60; −1.16]	0.9%
Lowe et al. 2014 light	10	−17.26	318.24	6	315.51	328.13	−0.98	[−2.06; 0.11]	0.5%
Lowe et al. 2014 moderate	12	60.61	293.36	6	315.51	328.13	−0.80	[−1.82; 0.23]	0.5%
Alves et al. 2012	42	16.79	4.25	42	19.60	5.11	−0.59	[−1.03; −0.15]	1.7%
Lowe et al. 2016	51	32.59	54.99	51	43.42	64.01	−0.18	[−0.57; 0.21]	1.9%
Oberste et al. 2016* light	30	84.03	50.70	10	82.90	74.17	0.02	[−0.70; 0.74]	0.9%
Gothe et al. 2013 comparable CG	15	421.41	53.28	30	419.03	60.46	0.04	[−0.58; 0.66]	1.1%
de Marco et al. 2014	47	65.15	73.20	47	61.98	75.91	0.04	[−0.36; 0.45]	1.8%
Weng et al. 2015	25	510.43	59.30	25	503.43	47.00	0.13	[−0.43; 0.68]	1.3%
Oberste et al. 2016* vigorous	30	95.57	46.67	10	82.90	74.17	0.23	[−0.49; 0.95]	0.9%
Abe et al. 2018 comparable_CG	13	181.80	79.40	26	157.10	105.00	0.25	[−0.42; 0.92]	1.0%
Oberste et al. 2016* moderate	30	109.30	75.44	10	82.90	74.17	0.34	[−0.38; 1.06]	0.9%
Random effects model	327			285			−0.21	[−0.44; 0.01]	13.5%
Heterogeneity: $I^2 = 70\%$, $\tau^2 = 0.0570$, $p < 0.01$									
**Random effects model**	**2436**			**1938**			**−0.26**	**[−0.34; −0.18]**	**100.0%**
Heterogeneity: $I^2 = 39\%$, $\tau^2 = 0.0557$, $p < 0.01$									

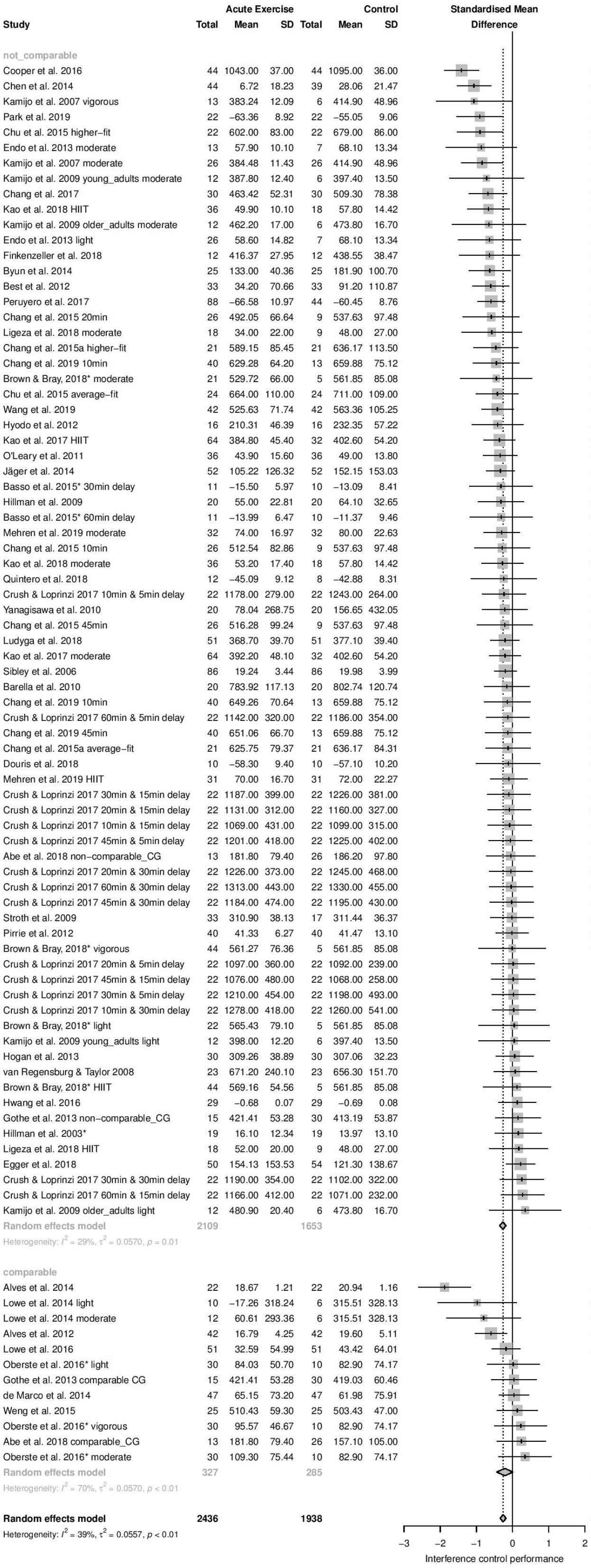

Figure 10 **Subgroup analysis for type of control group (time-dependent measures of interference control)**

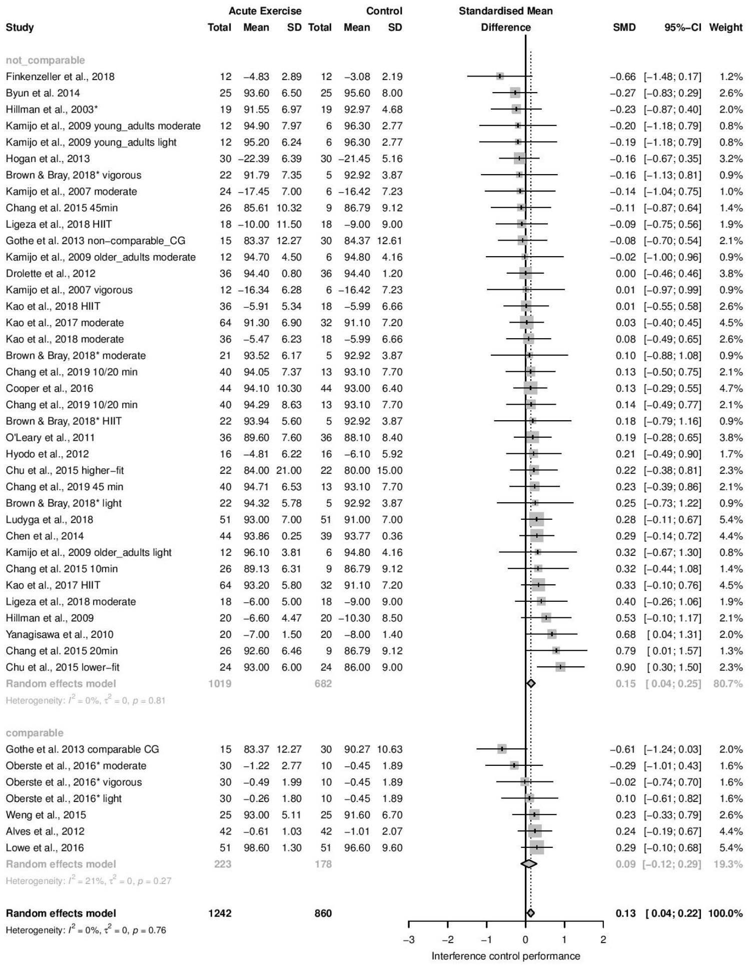

Figure 11 **Subgroup analysis for type of control group (accuracy measures of interference control)**

Study	Acute Exercise Total	Acute Exercise Mean	Acute Exercise SD	Control Total	Control Mean	Control SD	SMD	95%-CI	Weight
**no_familiarization**									
Lowe et al. 2014 light	10	−17.26	318.24	6	315.51	328.13	−0.98	[−2.06; 0.11]	0.5%
Park et al. 2019	22	−63.36	8.92	22	−55.05	9.06	−0.91	[−1.53; −0.28]	1.1%
Endo et al. 2013 moderate	13	57.90	10.10	7	68.10	13.34	−0.87	[−1.83; 0.10]	0.6%
Lowe et al. 2014 moderate	12	60.61	293.36	6	315.51	328.13	−0.80	[−1.82; 0.23]	0.5%
Endo et al. 2013 light	26	58.60	14.82	7	68.10	13.34	−0.64	[−1.49; 0.21]	0.7%
Basso et al. 2015* 30min delay	11	−15.50	5.97	10	−13.09	8.41	−0.32	[−1.18; 0.54]	0.7%
Basso et al. 2015* 60min delay	11	−13.99	6.47	10	−11.37	9.46	−0.31	[−1.18; 0.55]	0.7%
Crush & Loprinzi 2017 10min & 5min delay	22	1178.00	279.00	22	1243.00	264.00	−0.24	[−0.83; 0.36]	1.2%
Sibley et al. 2006	86	19.24	3.44	86	19.98	3.99	−0.20	[−0.50; 0.10]	2.2%
Lowe et al. 2016	51	32.59	54.99	51	43.42	64.01	−0.18	[−0.57; 0.21]	1.9%
Crush & Loprinzi 2017 60min & 5min delay	22	1142.00	320.00	22	1186.00	354.00	−0.13	[−0.72; 0.46]	1.2%
Douris et al. 2018	10	−58.30	9.40	10	−57.10	10.20	−0.12	[−0.99; 0.76]	0.7%
Crush & Loprinzi 2017 30min & 15min delay	22	1187.00	399.00	22	1226.00	381.00	−0.10	[−0.69; 0.49]	1.2%
Crush & Loprinzi 2017 20min & 15min delay	22	1131.00	312.00	22	1160.00	327.00	−0.09	[−0.68; 0.50]	1.2%
Crush & Loprinzi 2017 10min & 15min delay	22	1069.00	431.00	22	1099.00	315.00	−0.08	[−0.67; 0.51]	1.2%
Crush & Loprinzi 2017 45min & 5min delay	22	1201.00	418.00	22	1225.00	402.00	−0.06	[−0.65; 0.53]	1.2%
Crush & Loprinzi 2017 20min & 30min delay	22	1226.00	373.00	22	1245.00	468.00	−0.04	[−0.64; 0.55]	1.2%
Crush & Loprinzi 2017 60min & 30min delay	22	1313.00	443.00	22	1330.00	455.00	−0.04	[−0.63; 0.55]	1.2%
Crush & Loprinzi 2017 45min & 30min delay	22	1184.00	474.00	22	1195.00	430.00	−0.02	[−0.61; 0.57]	1.2%
Pirrie et al. 2012	40	41.33	6.27	40	41.47	13.10	−0.01	[−0.45; 0.42]	1.7%
Crush & Loprinzi 2017 20min & 5min delay	22	1097.00	360.00	22	1092.00	239.00	0.02	[−0.57; 0.61]	1.2%
Crush & Loprinzi 2017 45min & 15min delay	22	1076.00	480.00	22	1068.00	258.00	0.02	[−0.57; 0.61]	1.2%
Crush & Loprinzi 2017 30min & 5min delay	22	1210.00	454.00	22	1198.00	493.00	0.02	[−0.57; 0.62]	1.2%
Crush & Loprinzi 2017 10min & 30min delay	22	1278.00	418.00	22	1260.00	541.00	0.04	[−0.55; 0.63]	1.2%
Gothe et al. 2013 comparable CG	15	421.41	53.28	30	419.03	60.46	0.04	[−0.58; 0.66]	1.1%
de Marco et al. 2014	47	65.15	73.20	47	61.98	75.91	0.04	[−0.36; 0.45]	1.8%
van Regensburg & Taylor 2008	23	671.20	240.10	23	656.30	151.70	0.07	[−0.51; 0.65]	1.2%
Hwang et al. 2016	29	−0.68	0.07	29	−0.69	0.08	0.13	[−0.38; 0.65]	1.4%
Gothe et al. 2013 non−comparable_CG	15	421.41	53.28	30	413.19	53.87	0.15	[−0.47; 0.77]	1.1%
Hillman et al. 2003*	19	16.10	12.34	19	13.97	13.10	0.16	[−0.47; 0.80]	1.1%
Crush & Loprinzi 2017 30min & 30min delay	22	1190.00	354.00	22	1102.00	322.00	0.26	[−0.34; 0.85]	1.2%
Crush & Loprinzi 2017 60min & 15min delay	22	1166.00	412.00	22	1071.00	232.00	0.28	[−0.32; 0.87]	1.2%
Random effects model	770			763			−0.10	[−0.22; 0.03]	37.2%
Heterogeneity: $I^2 = 0\%$, $\tau^2 = 0.0397$, $p = 0.85$									
**familiarization**									
Alves et al. 2014	22	18.67	1.21	22	20.94	1.16	−1.88	[−2.60; −1.16]	0.9%
Cooper et al. 2016	44	1043.00	37.00	44	1095.00	36.00	−1.41	[−1.88; −0.94]	1.6%
Chen et al. 2014	44	6.72	18.23	39	28.06	21.47	−1.07	[−1.53; −0.60]	1.6%
Kamijo et al. 2007 vigorous	13	383.24	12.09	6	414.90	48.96	−1.06	[−2.10; −0.02]	0.5%
Chu et al. 2015 higher−fit	22	602.00	83.00	22	679.00	86.00	−0.89	[−1.52; −0.27]	1.1%
Kamijo et al. 2007 moderate	26	384.48	11.43	26	414.90	48.96	−0.84	[−1.41; −0.27]	1.3%
Kamijo et al. 2009 young_adults moderate	12	387.80	12.40	6	397.40	13.50	−0.72	[−1.73; 0.30]	0.5%
Chang et al. 2017	30	463.42	52.31	30	509.30	78.38	−0.68	[−1.20; −0.16]	1.4%
Kao et al. 2018 HIIT	36	49.90	10.10	18	57.80	14.42	−0.67	[−1.25; −0.09]	1.2%
Kamijo et al. 2009 older_adults moderate	12	462.20	17.00	6	473.80	16.70	−0.65	[−1.66; 0.36]	0.6%
Finkenzeller et al. 2018	12	416.37	27.95	12	438.55	38.47	−0.64	[−1.46; 0.19]	0.8%
Byun et al. 2014	25	133.00	40.36	25	181.90	100.70	−0.63	[−1.20; −0.06]	1.3%
Best et al. 2012	33	34.20	70.66	33	91.20	110.87	−0.61	[−1.10; −0.11]	1.5%
Alves et al. 2012	42	16.79	4.25	42	19.60	5.11	−0.59	[−1.03; −0.15]	1.7%
Peruyero et al. 2017	88	−66.58	10.97	44	−60.45	8.76	−0.59	[−0.96; −0.22]	1.9%
Chang et al. 2015 20min	26	492.05	66.64	9	537.63	97.48	−0.59	[−1.36; 0.18]	0.8%
Ligeza et al. 2018 moderate	18	34.00	22.00	9	48.00	27.00	−0.57	[−1.39; 0.24]	0.8%
Chang et al. 2015a higher−fit	21	589.15	85.45	21	636.17	113.50	−0.46	[−1.07; 0.15]	1.1%
Chang et al. 2019 10min	40	629.28	64.20	13	659.88	75.12	−0.45	[−1.08; 0.18]	1.1%
Brown & Bray, 2018* moderate	21	529.72	66.00	5	561.85	85.08	−0.45	[−1.43; 0.54]	0.6%
Chu et al. 2015 average−fit	24	664.00	110.00	24	711.00	109.00	−0.42	[−0.99; 0.15]	1.3%
Wang et al. 2019	42	525.63	71.74	42	563.36	105.25	−0.42	[−0.85; 0.02]	1.7%
Hyodo et al. 2012	16	210.31	46.39	16	232.35	57.22	−0.41	[−1.11; 0.29]	1.0%
Kao et al. 2017 HIIT	64	384.80	45.40	32	402.60	54.20	−0.36	[−0.79; 0.06]	1.7%
O'Leary et al. 2011	36	43.90	15.60	36	49.00	13.80	−0.34	[−0.81; 0.12]	1.6%
Jäger et al. 2014	52	105.22	126.32	52	152.15	153.03	−0.33	[−0.72; 0.06]	1.9%
Hillman et al. 2009	20	55.00	22.81	20	64.10	32.65	−0.32	[−0.94; 0.31]	1.1%
Mehren et al. 2019 moderate	32	74.00	16.97	32	80.00	22.63	−0.30	[−0.79; 0.20]	1.5%
Chang et al. 2015 10min	26	512.54	82.86	9	537.63	97.48	−0.28	[−1.04; 0.48]	0.9%
Kao et al. 2018 moderate	36	53.20	17.40	18	57.80	14.42	−0.27	[−0.84; 0.29]	1.3%
Quintero et al. 2018	12	−45.09	9.12	8	−42.88	8.31	−0.24	[−1.14; 0.66]	0.7%
Yanagisawa et al. 2010	20	78.04	268.75	20	156.65	432.05	−0.21	[−0.84; 0.41]	1.1%
Chang et al. 2015 45min	26	516.28	99.24	9	537.63	97.48	−0.21	[−0.97; 0.55]	0.9%
Ludyga et al. 2018	51	368.70	39.70	51	377.10	39.40	−0.21	[−0.60; 0.18]	1.9%
Kao et al. 2017 moderate	64	392.20	48.10	32	402.60	54.20	−0.21	[−0.63; 0.22]	1.7%
Barella et al. 2010	20	783.92	117.13	20	802.74	120.74	−0.16	[−0.78; 0.47]	1.1%
Chang et al. 2019 10min	40	649.26	70.64	13	659.88	75.12	−0.15	[−0.77; 0.48]	1.1%
Chang et al. 2019 45min	40	651.06	66.70	13	659.88	75.12	−0.13	[−0.75; 0.50]	1.1%
Chang et al. 2015a average−fit	21	625.75	79.37	21	636.17	84.31	−0.12	[−0.73; 0.48]	1.2%
Mehren et al. 2019 HIIT	31	70.00	16.70	31	72.00	22.27	−0.10	[−0.60; 0.40]	1.5%
Abe et al. 2018 non−comparable_CG	13	181.80	79.40	26	186.20	97.80	−0.05	[−0.71; 0.62]	1.0%
Stroth et al. 2009	33	310.90	38.13	17	311.44	36.37	−0.01	[−0.60; 0.57]	1.2%
Brown & Bray, 2018* vigorous	44	561.27	76.36	5	561.85	85.08	−0.01	[−0.93; 0.92]	0.6%
Oberste et al. 2016* light	30	84.03	50.70	10	82.90	74.17	0.02	[−0.70; 0.74]	0.9%
Brown & Bray, 2018* light	22	565.43	79.10	5	561.85	85.08	0.04	[−0.93; 1.01]	0.6%
Kamijo et al. 2009 young_adults light	12	398.00	12.20	6	397.40	13.50	0.05	[−0.93; 1.03]	0.6%
Hogan et al. 2013	30	309.26	38.89	30	307.06	32.23	0.06	[−0.45; 0.57]	1.4%
Brown & Bray, 2018* HIIT	44	569.16	54.56	5	561.85	85.08	0.12	[−0.80; 1.05]	0.6%
Weng et al. 2015	25	510.43	59.30	25	503.43	47.00	0.13	[−0.43; 0.68]	1.3%
Ligeza et al. 2018 HIIT	18	52.00	20.00	9	48.00	27.00	0.17	[−0.63; 0.97]	0.8%
Egger et al. 2018	50	154.13	153.53	54	121.30	138.67	0.22	[−0.16; 0.61]	1.9%
Oberste et al. 2016* vigorous	30	95.57	46.67	10	82.90	74.17	0.23	[−0.49; 0.95]	0.9%
Abe et al. 2018 comparable_CG	13	181.80	79.40	26	157.10	105.00	0.25	[−0.42; 0.92]	1.0%
Oberste et al. 2016* moderate	30	109.30	75.44	10	82.90	74.17	0.34	[−0.38; 1.06]	0.9%
Kamijo et al. 2009 older_adults light	12	480.90	20.40	6	473.80	16.70	0.35	[−0.64; 1.34]	0.6%
Random effects model	1666			1175			−0.36	[−0.46; −0.26]	62.8%
Heterogeneity: $I^2 = 46\%$, $\tau^2 = 0.0397$, $p < 0.01$									
**Random effects model**	**2436**			**1938**			**−0.26**	**[−0.34; −0.18]**	**100.0%**
Heterogeneity: $I^2 = 39\%$, $\tau^2 = 0.0557$, $p < 0.01$									

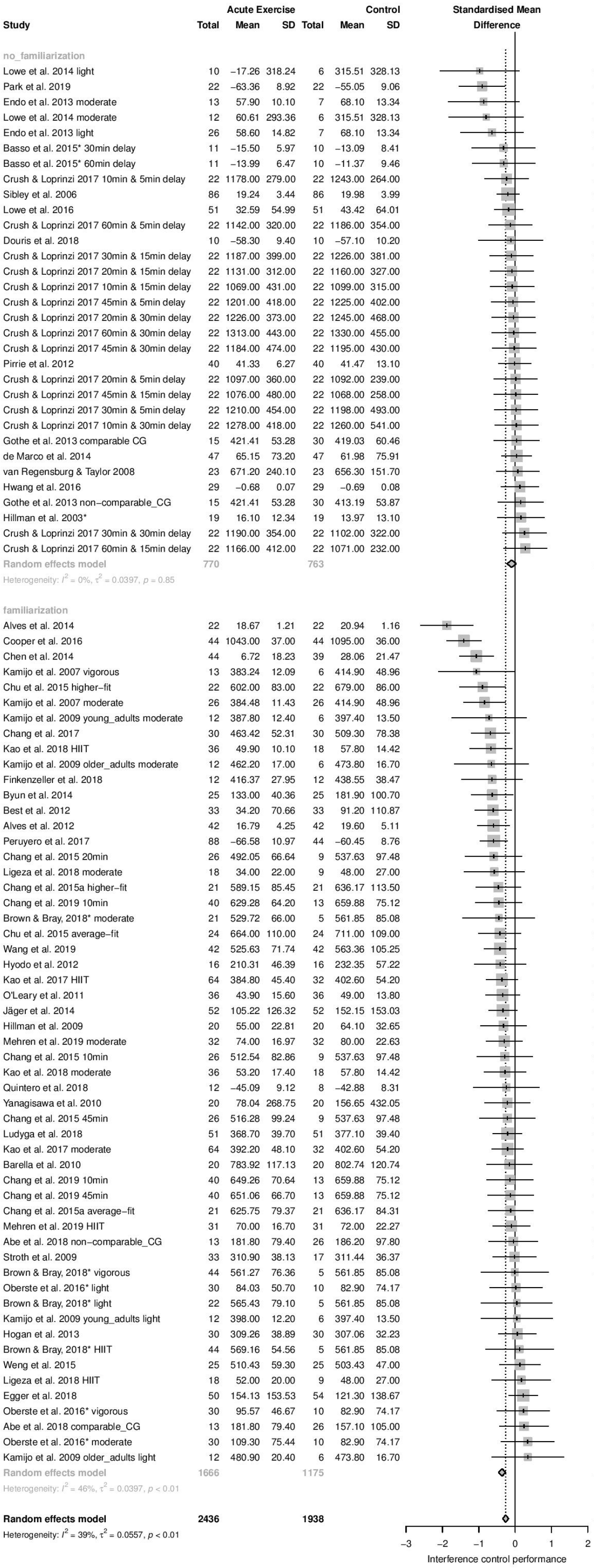

Figure 12 Subgroup analysis for familiarization with cognitive testing procedure prior to start of experiment (time-dependent measures of interference control)

Study	Acute Exercise Total	Acute Exercise Mean	Acute Exercise SD	Control Total	Control Mean	Control SD	SMD	95%-CI	Weight
familiarization									
Finkenzeller et al., 2018	12	−4.83	2.89	12	−3.08	2.19	−0.66	[−1.48; 0.17]	1.2%
Oberste et al., 2016* moderate	30	−1.22	2.77	10	−0.45	1.89	−0.29	[−1.01; 0.43]	1.6%
Byun et al. 2014	25	93.60	6.50	25	95.60	8.00	−0.27	[−0.83; 0.29]	2.6%
Kamijo et al., 2009 young_adults moderate	12	94.90	7.97	6	96.30	2.77	−0.20	[−1.18; 0.79]	0.8%
Kamijo et al., 2009 young_adults light	12	95.20	6.24	6	96.30	2.77	−0.19	[−1.18; 0.79]	0.8%
Hogan et al., 2013	30	−22.39	6.39	30	−21.45	5.16	−0.16	[−0.67; 0.35]	3.2%
Brown & Bray, 2018* vigorous	22	91.79	7.35	5	92.92	3.87	−0.16	[−1.13; 0.81]	0.9%
Kamijo et al., 2007 moderate	24	−17.45	7.00	6	−16.42	7.23	−0.14	[−1.04; 0.75]	1.0%
Chang et al. 2015 45min	26	85.61	10.32	9	86.79	9.12	−0.11	[−0.87; 0.64]	1.4%
Ligeza et al., 2018 HIIT	18	−10.00	11.50	18	−9.00	9.00	−0.09	[−0.75; 0.56]	1.9%
Kamijo et al., 2009 older_adults moderate	12	94.70	4.50	6	94.80	4.16	−0.02	[−1.00; 0.96]	0.9%
Oberste et al., 2016* vigorous	30	−0.49	1.99	10	−0.45	1.89	−0.02	[−0.74; 0.70]	1.6%
Drolette et al., 2012	36	94.40	0.80	36	94.40	1.20	0.00	[−0.46; 0.46]	3.8%
Kamijo et al., 2007 vigorous	12	−16.34	6.28	6	−16.42	7.23	0.01	[−0.97; 0.99]	0.9%
Kao et al., 2018 HIIT	36	−5.91	5.34	18	−5.99	6.66	0.01	[−0.55; 0.58]	2.6%
Kao et al., 2017 moderate	64	91.30	6.90	32	91.10	7.20	0.03	[−0.40; 0.45]	4.5%
Kao et al., 2018 moderate	36	−5.47	6.23	18	−5.99	6.66	0.08	[−0.49; 0.65]	2.6%
Brown & Bray, 2018* moderate	21	93.52	6.17	5	92.92	3.87	0.10	[−0.88; 1.08]	0.9%
Oberste et al., 2016* light	30	−0.26	1.80	10	−0.45	1.89	0.10	[−0.61; 0.82]	1.6%
Chang et al., 2019 10/20 min	40	94.05	7.37	13	93.10	7.70	0.13	[−0.50; 0.75]	2.1%
Cooper et al., 2016	44	94.10	10.30	44	93.00	6.40	0.13	[−0.29; 0.55]	4.7%
Chang et al., 2019 10/20 min	40	94.29	8.63	13	93.10	7.70	0.14	[−0.49; 0.77]	2.1%
Brown & Bray, 2018* HIIT	22	93.94	5.60	5	92.92	3.87	0.18	[−0.79; 1.16]	0.9%
O'Leary et al., 2011	36	89.60	7.60	36	88.10	8.40	0.19	[−0.28; 0.65]	3.8%
Hyodo et al., 2012	16	−4.81	6.22	16	−6.10	5.92	0.21	[−0.49; 0.90]	1.7%
Chu et al., 2015 higher−fit	22	84.00	21.00	22	80.00	15.00	0.22	[−0.38; 0.81]	2.3%
Weng et al., 2015	25	93.00	5.11	25	91.60	6.70	0.23	[−0.33; 0.79]	2.6%
Chang et al., 2019 45 min	40	94.71	6.53	13	93.10	7.70	0.23	[−0.39; 0.86]	2.1%
Alves et al., 2012	42	−0.61	1.03	42	−1.01	2.07	0.24	[−0.19; 0.67]	4.4%
Brown & Bray, 2018* light	22	94.32	5.78	5	92.92	3.87	0.25	[−0.73; 1.22]	0.9%
Ludyga et al., 2018	51	93.00	7.00	51	91.00	7.00	0.28	[−0.11; 0.67]	5.4%
Chen et al., 2014	44	93.86	0.25	39	93.77	0.36	0.29	[−0.14; 0.72]	4.4%
Kamijo et al., 2009 older_adults light	12	96.10	3.81	6	94.80	4.16	0.32	[−0.67; 1.30]	0.8%
Chang et al. 2015 10min	26	89.13	6.31	9	86.79	9.12	0.32	[−0.44; 1.08]	1.4%
Kao et al., 2017 HIIT	64	93.20	5.80	32	91.10	7.20	0.33	[−0.10; 0.76]	4.5%
Ligeza et al., 2018 moderate	18	−6.00	5.00	18	−9.00	9.00	0.40	[−0.26; 1.06]	1.9%
Hillman et al., 2009	20	−6.60	4.47	20	−10.30	8.50	0.53	[−0.10; 1.17]	2.1%
Yanagisawa et al., 2010	20	−7.00	1.50	20	−8.00	1.40	0.68	[0.04; 1.31]	2.0%
Chang et al. 2015 20min	26	92.60	6.46	9	86.79	9.12	0.79	[0.01; 1.57]	1.3%
Chu et al., 2015 lower−fit	24	93.00	6.00	24	86.00	9.00	0.90	[0.30; 1.50]	2.3%
Random effects model	1142			730			0.16	[0.06; 0.25]	88.4%
Heterogeneity: $I^2 = 0\%$, $\tau^2 = 0$, $p = 0.89$									
no_familiarization									
Gothe et al. 2013 comparable CG	15	83.37	12.27	30	90.27	10.63	−0.61	[−1.24; 0.03]	2.0%
Hillman et al., 2003*	19	91.55	6.97	19	92.97	4.68	−0.23	[−0.87; 0.40]	2.0%
Gothe et al. 2013 non−comparable_CG	15	83.37	12.27	30	84.37	12.61	−0.08	[−0.70; 0.54]	2.1%
Lowe et al., 2016	51	98.60	1.30	51	96.60	9.60	0.29	[−0.10; 0.68]	5.4%
Random effects model	100			130			−0.03	[−0.29; 0.24]	11.6%
Heterogeneity: $I^2 = 51\%$, $\tau^2 = 0$, $p = 0.10$									
**Random effects model**	**1242**			**860**			**0.13**	**[0.04; 0.22]**	**100.0%**
Heterogeneity: $I^2 = 0\%$, $\tau^2 = 0$, $p = 0.76$									

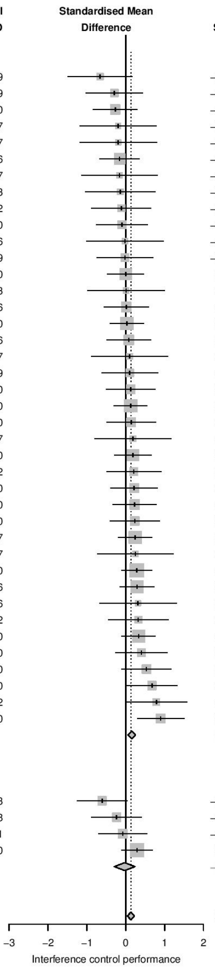

Figure 13 **Subgroup analysis for familiarization with cognitive testing procedure prior to start of experiment (accuracy measures of interference control)**

Study	Acute Exercise Total	Acute Exercise Mean	Acute Exercise SD	Control Total	Control Mean	Control SD	SMD	95%-CI	Weight
**interference_variable_available**									
Chen et al. 2014	44	6.72	18.23	39	28.06	21.47	−1.07	[−1.53; −0.60]	1.6%
Lowe et al. 2014 light	10	−17.26	318.24	6	315.51	328.13	−0.98	[−2.06; 0.11]	0.5%
Lowe et al. 2014 moderate	12	60.61	293.36	6	315.51	328.13	−0.80	[−1.82; 0.23]	0.5%
Kao et al. 2018 HIIT	36	49.90	10.10	18	57.80	14.42	−0.67	[−1.25; −0.09]	1.2%
Byun et al. 2014	25	133.00	40.36	25	181.90	100.70	−0.63	[−1.20; −0.06]	1.3%
Best et al. 2012	33	34.20	70.66	33	91.20	110.87	−0.61	[−1.10; −0.11]	1.5%
Ligeza et al. 2018 moderate	18	34.00	22.00	9	48.00	27.00	−0.57	[−1.39; 0.24]	0.8%
Hyodo et al. 2012	16	210.31	46.39	16	232.35	57.22	−0.41	[−1.11; 0.29]	1.0%
O'Leary et al. 2011	36	43.90	15.60	36	49.00	13.80	−0.34	[−0.81; 0.12]	1.6%
Jäger et al. 2014	52	105.22	126.32	52	152.15	153.03	−0.33	[−0.72; 0.06]	1.9%
Basso et al. 2015* 30min delay	11	−15.50	5.97	10	−13.09	8.41	−0.32	[−1.18; 0.54]	0.7%
Hillman et al. 2009	20	55.00	22.81	20	64.10	32.65	−0.32	[−0.94; 0.31]	1.1%
Basso et al. 2015* 60min delay	11	−13.99	6.47	10	−11.37	9.46	−0.31	[−1.18; 0.55]	0.7%
Mehren et al. 2019 moderate	32	74.00	16.97	32	80.00	22.63	−0.30	[−0.79; 0.20]	1.5%
Kao et al. 2018 moderate	36	53.20	17.40	18	57.80	14.42	−0.27	[−0.84; 0.29]	1.3%
Yanagisawa et al. 2010	20	78.04	268.75	20	156.65	432.05	−0.21	[−0.84; 0.41]	1.1%
Lowe et al. 2016	51	32.59	54.99	51	43.42	64.01	−0.18	[−0.57; 0.21]	1.9%
Mehren et al. 2019 HIIT	31	70.00	16.70	31	72.00	22.27	−0.10	[−0.60; 0.40]	1.5%
Abe et al. 2018 non−comparable_CG	13	181.80	79.40	26	186.20	97.80	−0.05	[−0.71; 0.62]	1.0%
Oberste et al. 2016* light	30	84.03	50.70	10	82.90	74.17	0.02	[−0.70; 0.74]	0.9%
de Marco et al. 2014	47	65.15	73.20	47	61.98	75.91	0.04	[−0.36; 0.45]	1.8%
Hwang et al. 2016	29	−0.68	0.07	29	−0.69	0.08	0.13	[−0.38; 0.65]	1.4%
Hillman et al. 2003*	19	16.10	12.34	19	13.97	13.10	0.16	[−0.47; 0.80]	1.1%
Ligeza et al. 2018 HIIT	18	52.00	20.00	9	48.00	27.00	0.17	[−0.63; 0.97]	0.8%
Egger et al. 2018	50	154.13	153.53	54	121.30	138.67	0.22	[−0.16; 0.61]	1.9%
Oberste et al. 2016* vigorous	30	95.57	46.67	10	82.90	74.17	0.23	[−0.49; 0.95]	0.9%
Abe et al. 2018 comparable_CG	13	181.80	79.40	26	157.10	105.00	0.25	[−0.42; 0.92]	1.0%
Oberste et al. 2016* moderate	30	109.30	75.44	10	82.90	74.17	0.34	[−0.38; 1.06]	0.9%
Random effects model	773			672			−0.23	[−0.37; −0.09]	33.3%
Heterogeneity: $I^2 = 36\%$, $\tau^2 = 0.0570$, $p = 0.03$									
**interference_variable_not_available**									
Alves et al. 2014	22	18.67	1.21	22	20.94	1.16	−1.88	[−2.60; −1.16]	0.9%
Cooper et al. 2016	44	1043.00	37.00	44	1095.00	36.00	−1.41	[−1.88; −0.94]	1.6%
Kamijo et al. 2007 vigorous	13	383.24	12.09	6	414.90	48.96	−1.06	[−2.10; −0.02]	0.5%
Park et al. 2019	22	−63.36	8.92	22	−55.05	9.06	−0.91	[−1.53; −0.28]	1.1%
Chu et al. 2015 higher−fit	22	602.00	83.00	22	679.00	86.00	−0.89	[−1.52; −0.27]	1.1%
Endo et al. 2013 moderate	13	57.90	10.10	7	68.10	13.34	−0.87	[−1.83; 0.10]	0.6%
Kamijo et al. 2007 moderate	26	384.48	11.43	26	414.90	48.96	−0.84	[−1.41; −0.27]	1.3%
Kamijo et al. 2009 young_adults moderate	12	387.80	12.40	6	397.40	13.50	−0.72	[−1.73; 0.30]	0.5%
Chang et al. 2017	30	463.42	52.31	30	509.30	78.38	−0.68	[−1.20; −0.16]	1.4%
Kamijo et al. 2009 older_adults moderate	12	462.20	17.00	6	473.80	16.70	−0.65	[−1.66; 0.36]	0.6%
Endo et al. 2013 light	26	58.60	14.82	7	68.10	13.34	−0.64	[−1.49; 0.21]	0.7%
Finkenzeller et al. 2018	12	416.37	27.95	12	438.55	38.47	−0.64	[−1.46; 0.19]	0.8%
Alves et al. 2012	42	16.79	4.25	42	19.60	5.11	−0.59	[−1.03; −0.15]	1.7%
Peruyero et al. 2017	88	−66.58	10.97	44	−60.45	8.76	−0.59	[−0.96; −0.22]	1.9%
Chang et al. 2015 20min	26	492.05	66.64	9	537.63	97.48	−0.59	[−1.36; 0.18]	0.8%
Chang et al. 2015a higher−fit	21	589.15	85.45	21	636.17	113.50	−0.46	[−1.07; 0.15]	1.1%
Chang et al. 2019 10min	40	629.28	64.20	13	659.88	75.12	−0.45	[−1.08; 0.18]	1.1%
Brown & Bray, 2018* moderate	21	529.72	66.00	5	561.85	85.08	−0.45	[−1.43; 0.54]	0.6%
Chu et al. 2015 average−fit	24	664.00	110.00	24	711.00	109.00	−0.42	[−0.99; 0.15]	1.3%
Wang et al. 2019	42	525.63	71.74	42	563.36	105.25	−0.42	[−0.85; 0.02]	1.7%
Kao et al. 2017 HIIT	64	384.80	45.40	32	402.60	54.20	−0.36	[−0.79; 0.06]	1.7%
Chang et al. 2015 10min	26	512.54	82.86	9	537.63	97.48	−0.28	[−1.04; 0.48]	0.9%
Quintero et al. 2018	12	−45.09	9.12	8	−42.88	8.31	−0.24	[−1.14; 0.66]	0.7%
Crush & Loprinzi 2017 10min & 5min delay	22	1178.00	279.00	22	1243.00	264.00	−0.24	[−0.83; 0.36]	1.2%
Chang et al. 2015 45min	26	516.28	99.24	9	537.63	97.48	−0.21	[−0.97; 0.55]	0.9%
Ludyga et al. 2018	51	368.70	39.70	51	377.10	39.40	−0.21	[−0.60; 0.18]	1.9%
Kao et al. 2017 moderate	64	392.20	48.10	32	402.60	54.20	−0.21	[−0.63; 0.22]	1.7%
Sibley et al. 2006	86	19.24	3.44	86	19.98	3.99	−0.20	[−0.50; 0.10]	2.2%
Barella et al. 2010	20	783.92	117.13	20	802.74	120.74	−0.16	[−0.78; 0.47]	1.1%
Chang et al. 2019 10min	40	649.26	70.64	13	659.88	75.12	−0.15	[−0.77; 0.48]	1.1%
Crush & Loprinzi 2017 60min & 5min delay	22	1142.00	320.00	22	1186.00	354.00	−0.13	[−0.72; 0.46]	1.2%
Chang et al. 2019 45min	40	651.06	66.70	13	659.88	75.12	−0.13	[−0.75; 0.50]	1.1%
Chang et al. 2015a average−fit	21	625.75	79.37	21	636.17	84.31	−0.12	[−0.73; 0.48]	1.2%
Douris et al. 2018	10	−58.30	9.40	10	−57.10	10.20	−0.12	[−0.99; 0.76]	0.7%
Crush & Loprinzi 2017 30min & 15min delay	22	1187.00	399.00	22	1226.00	381.00	−0.10	[−0.69; 0.49]	1.2%
Crush & Loprinzi 2017 20min & 15min delay	22	1131.00	312.00	22	1160.00	327.00	−0.09	[−0.68; 0.50]	1.2%
Crush & Loprinzi 2017 10min & 15min delay	22	1069.00	431.00	22	1099.00	315.00	−0.08	[−0.67; 0.51]	1.2%
Crush & Loprinzi 2017 45min & 5min delay	22	1201.00	418.00	22	1225.00	402.00	−0.06	[−0.65; 0.53]	1.2%
Crush & Loprinzi 2017 20min & 30min delay	22	1226.00	373.00	22	1245.00	468.00	−0.04	[−0.64; 0.55]	1.2%
Crush & Loprinzi 2017 60min & 30min delay	22	1313.00	443.00	22	1330.00	455.00	−0.04	[−0.63; 0.55]	1.2%
Crush & Loprinzi 2017 45min & 30min delay	22	1184.00	474.00	22	1195.00	430.00	−0.02	[−0.61; 0.57]	1.2%
Stroth et al. 2009	33	310.90	38.13	17	311.44	36.37	−0.01	[−0.60; 0.57]	1.2%
Pirrie et al. 2012	40	41.33	6.27	40	41.47	13.10	−0.01	[−0.45; 0.42]	1.7%
Brown & Bray, 2018* vigorous	44	561.27	76.36	5	561.85	85.08	−0.01	[−0.93; 0.92]	0.6%
Crush & Loprinzi 2017 20min & 5min delay	22	1097.00	360.00	22	1092.00	239.00	0.02	[−0.57; 0.61]	1.2%
Crush & Loprinzi 2017 45min & 15min delay	22	1076.00	480.00	22	1068.00	258.00	0.02	[−0.57; 0.61]	1.2%
Crush & Loprinzi 2017 30min & 5min delay	22	1210.00	454.00	22	1198.00	493.00	0.02	[−0.57; 0.62]	1.2%
Crush & Loprinzi 2017 10min & 30min delay	22	1278.00	418.00	22	1260.00	541.00	0.04	[−0.55; 0.63]	1.2%
Gothe et al. 2013 comparable CG	15	421.41	53.28	30	419.03	60.46	0.04	[−0.58; 0.66]	1.1%
Brown & Bray, 2018* light	22	565.43	79.10	5	561.85	85.08	0.04	[−0.93; 1.01]	0.6%
Kamijo et al. 2009 young_adults light	12	398.00	12.20	6	397.40	13.50	0.05	[−0.93; 1.03]	0.6%
Hogan et al. 2013	30	309.26	38.89	30	307.06	32.23	0.06	[−0.45; 0.57]	1.4%
van Regensburg & Taylor 2008	23	671.20	240.10	23	656.30	151.70	0.07	[−0.51; 0.65]	1.2%
Brown & Bray, 2018* HIIT	44	569.16	54.56	5	561.85	85.08	0.12	[−0.80; 1.05]	0.6%
Weng et al. 2015	25	510.43	59.30	25	503.43	47.00	0.13	[−0.43; 0.68]	1.3%
Gothe et al. 2013 non−comparable_CG	15	421.41	53.28	30	413.19	53.87	0.15	[−0.47; 0.77]	1.1%
Crush & Loprinzi 2017 30min & 30min delay	22	1190.00	354.00	22	1102.00	322.00	0.26	[−0.34; 0.85]	1.2%
Crush & Loprinzi 2017 60min & 15min delay	22	1166.00	412.00	22	1071.00	232.00	0.28	[−0.32; 0.87]	1.2%
Kamijo et al. 2009 older_adults light	12	480.90	20.40	6	473.80	16.70	0.35	[−0.64; 1.34]	0.6%
Random effects model	1663			1266			−0.28	[−0.38; −0.17]	66.7%
Heterogeneity: $I^2 = 41\%$, $\tau^2 = 0.0570$, $p < 0.01$									
**Random effects model**	**2436**			**1938**			**−0.26**	**[−0.34; −0.18]**	**100.0%**
Heterogeneity: $I^2 = 39\%$, $\tau^2 = 0.0557$, $p < 0.01$									

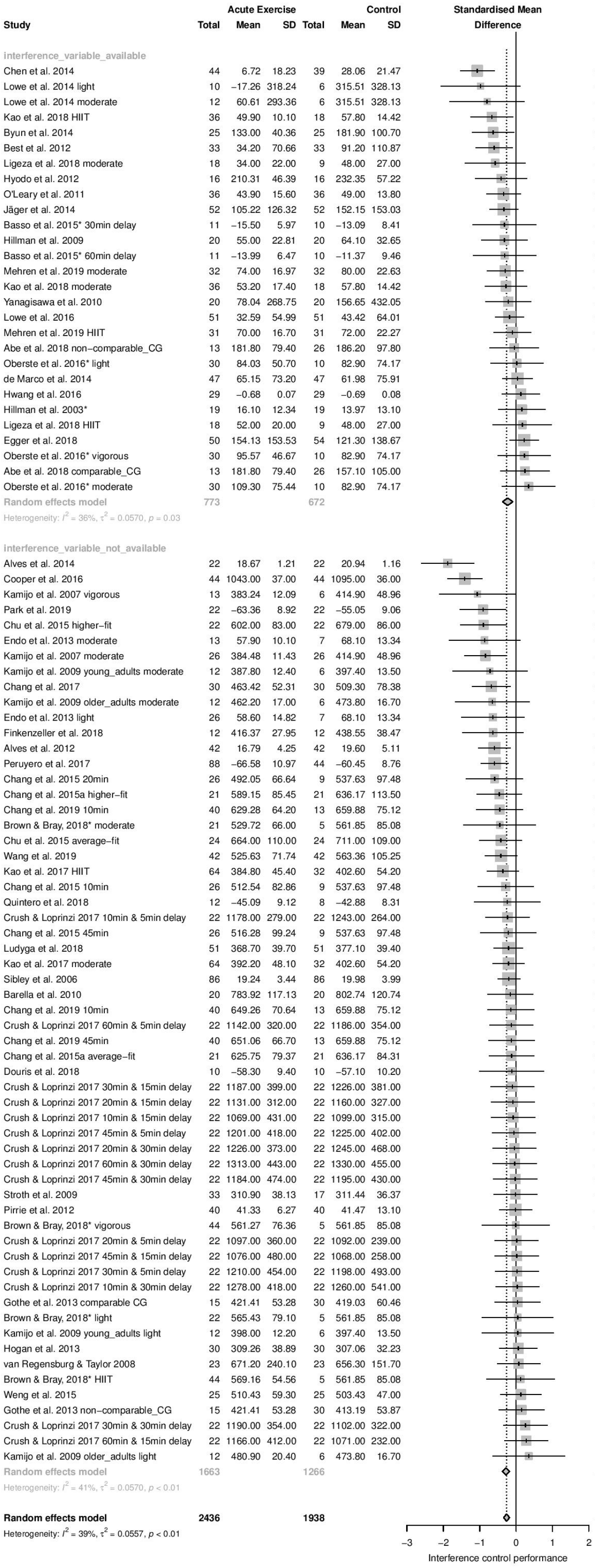

Figure 14 Subgroup analysis for type of variable used to measure interference control (time-dependent measures of interference control)

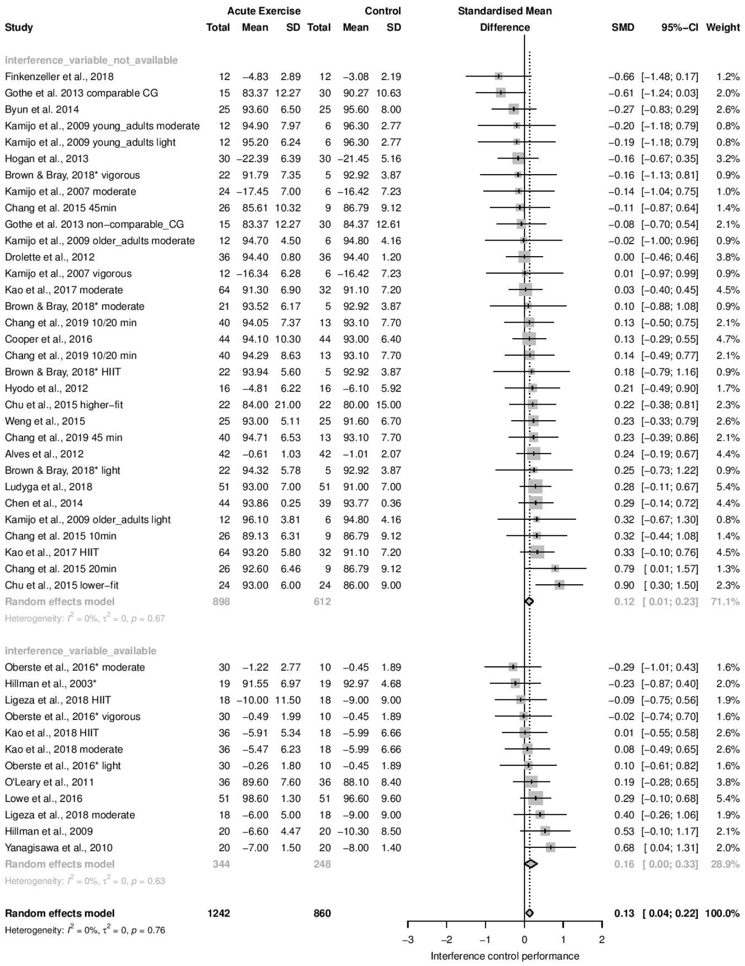

Figure 15 Subgroup analysis for type of variable used to measure interference control (accuracy measures of interference control)

Study	Acute Exercise Total	Acute Exercise Mean	Acute Exercise SD	Control Total	Control Mean	Control SD	SMD	95%-CI	Weight
**more_than_15_min.**									
Chen et al. 2014	44	6.72	18.23	39	28.06	21.47	−1.07	[−1.53; −0.60]	1.6%
Kao et al. 2017 HIIT	64	384.80	45.40	32	402.60	54.20	−0.36	[−0.79; 0.06]	1.8%
O'Leary et al. 2011	36	43.90	15.60	36	49.00	13.80	−0.34	[−0.81; 0.12]	1.6%
Basso et al. 2015* 30min delay	11	−15.50	5.97	10	−13.09	8.41	−0.32	[−1.18; 0.54]	0.7%
Hillman et al. 2009	20	55.00	22.81	20	64.10	32.65	−0.32	[−0.94; 0.31]	1.2%
Basso et al. 2015* 60min delay	11	−13.99	6.47	10	−11.37	9.46	−0.31	[−1.18; 0.55]	0.7%
Kao et al. 2017 moderate	64	392.20	48.10	32	402.60	54.20	−0.21	[−0.63; 0.22]	1.8%
Crush & Loprinzi 2017 20min & 30min delay	22	1226.00	373.00	22	1245.00	468.00	−0.04	[−0.64; 0.55]	1.3%
Crush & Loprinzi 2017 60min & 30min delay	22	1313.00	443.00	22	1330.00	455.00	−0.04	[−0.63; 0.55]	1.3%
Crush & Loprinzi 2017 45min & 30min delay	22	1184.00	474.00	22	1195.00	430.00	−0.02	[−0.61; 0.57]	1.3%
Crush & Loprinzi 2017 10min & 30min delay	22	1278.00	418.00	22	1260.00	541.00	0.04	[−0.55; 0.63]	1.3%
Hillman et al. 2003*	19	16.10	12.34	19	13.97	13.10	0.16	[−0.47; 0.80]	1.1%
Crush & Loprinzi 2017 30min & 30min delay	22	1190.00	354.00	22	1102.00	322.00	0.26	[−0.34; 0.85]	1.2%
Random effects model	379			308			−0.22	[−0.43; −0.01]	16.8%
Heterogeneity: $I^2 = 38\%$, $\tau^2 = 0.0611$, $p = 0.08$									
**immediately_to_15_min.**									
Alves et al. 2014	22	18.67	1.21	22	20.94	1.16	−1.88	[−2.60; −1.16]	1.0%
Cooper et al. 2016	44	1043.00	37.00	44	1095.00	36.00	−1.41	[−1.88; −0.94]	1.6%
Kamijo et al. 2007 vigorous	13	383.24	12.09	6	414.90	48.96	−1.06	[−2.10; −0.02]	0.6%
Lowe et al. 2014 light	10	−17.26	318.24	6	315.51	328.13	−0.98	[−2.06; 0.11]	0.5%
Park et al. 2019	22	−63.36	8.92	22	−55.05	9.06	−0.91	[−1.53; −0.28]	1.2%
Chu et al. 2015 higher−fit	22	602.00	83.00	22	679.00	86.00	−0.89	[−1.52; −0.27]	1.2%
Endo et al. 2013 moderate	13	57.90	10.10	7	68.10	13.34	−0.87	[−1.83; 0.10]	0.6%
Kamijo et al. 2007 moderate	26	384.48	11.43	26	414.90	48.96	−0.84	[−1.41; −0.27]	1.3%
Lowe et al. 2014 moderate	12	60.61	293.36	6	315.51	328.13	−0.80	[−1.82; 0.23]	0.6%
Kamijo et al. 2009 young_adults moderate	12	387.80	12.40	6	397.40	13.50	−0.72	[−1.73; 0.30]	0.6%
Chang et al. 2017	30	463.42	52.31	30	509.30	78.38	−0.68	[−1.20; −0.16]	1.4%
Kao et al. 2018 HIIT	36	49.90	10.10	18	57.80	14.42	−0.67	[−1.25; −0.09]	1.3%
Kamijo et al. 2009 older_adults moderate	12	462.20	17.00	6	473.80	16.70	−0.65	[−1.66; 0.36]	0.6%
Endo et al. 2013 light	26	58.60	14.82	7	68.10	13.34	−0.64	[−1.49; 0.21]	0.8%
Finkenzeller et al. 2018	12	416.37	27.95	12	438.55	38.47	−0.64	[−1.46; 0.19]	0.8%
Byun et al. 2014	25	133.00	40.36	25	181.90	100.70	−0.63	[−1.20; −0.06]	1.3%
Best et al. 2012	33	34.20	70.66	33	91.20	110.87	−0.61	[−1.10; −0.11]	1.5%
Alves et al. 2012	42	16.79	4.25	42	19.60	5.11	−0.59	[−1.03; −0.15]	1.7%
Peruyero et al. 2017	88	−66.58	10.97	44	−60.45	8.76	−0.59	[−0.96; −0.22]	2.0%
Chang et al. 2015 20min	26	492.05	66.64	9	537.63	97.48	−0.59	[−1.36; 0.18]	0.9%
Ligeza et al. 2018 moderate	18	34.00	22.00	9	48.00	27.00	−0.57	[−1.39; 0.24]	0.8%
Chang et al. 2015a higher−fit	21	589.15	85.45	21	636.17	113.50	−0.46	[−1.07; 0.15]	1.2%
Chang et al. 2019 10min	40	629.28	64.20	13	659.88	75.12	−0.45	[−1.08; 0.18]	1.2%
Brown & Bray, 2018* moderate	21	529.72	66.00	5	561.85	85.08	−0.45	[−1.43; 0.54]	0.6%
Chu et al. 2015 average−fit	24	664.00	110.00	24	711.00	109.00	−0.42	[−0.99; 0.15]	1.3%
Wang et al. 2019	42	525.63	71.74	42	563.36	105.25	−0.42	[−0.85; 0.02]	1.7%
Hyodo et al. 2012	16	210.31	46.39	16	232.35	57.22	−0.41	[−1.11; 0.29]	1.0%
Jäger et al. 2014	52	105.22	126.32	52	152.15	153.03	−0.33	[−0.72; 0.06]	1.9%
Mehren et al. 2019 moderate	32	74.00	16.97	32	80.00	22.63	−0.30	[−0.79; 0.20]	1.5%
Chang et al. 2015 10min	26	512.54	82.86	9	537.63	97.48	−0.28	[−1.04; 0.48]	0.9%
Kao et al. 2018 moderate	36	53.20	17.40	18	57.80	14.42	−0.27	[−0.84; 0.29]	1.3%
Quintero et al. 2018	12	−45.09	9.12	8	−42.88	8.31	−0.24	[−1.14; 0.66]	0.7%
Crush & Loprinzi 2017 10min & 5min delay	22	1178.00	279.00	22	1243.00	264.00	−0.24	[−0.83; 0.36]	1.2%
Yanagisawa et al. 2010	20	78.04	268.75	20	156.65	432.05	−0.21	[−0.84; 0.41]	1.2%
Chang et al. 2015 45min	26	516.28	99.24	9	537.63	97.48	−0.21	[−0.97; 0.55]	0.9%
Sibley et al. 2006	86	19.24	3.44	86	19.98	3.99	−0.20	[−0.50; 0.10]	2.3%
Lowe et al. 2016	51	32.59	54.99	51	43.42	64.01	−0.18	[−0.57; 0.21]	1.9%
Barella et al. 2010	20	783.92	117.13	20	802.74	120.74	−0.16	[−0.78; 0.47]	1.2%
Chang et al. 2019 10min	40	649.26	70.64	13	659.88	75.12	−0.15	[−0.77; 0.48]	1.2%
Crush & Loprinzi 2017 60min & 5min delay	22	1142.00	320.00	22	1186.00	354.00	−0.13	[−0.72; 0.46]	1.2%
Chang et al. 2019 45min	40	651.06	66.70	13	659.88	75.12	−0.13	[−0.75; 0.50]	1.2%
Chang et al. 2015a average−fit	21	625.75	79.37	21	636.17	84.31	−0.12	[−0.73; 0.48]	1.2%
Douris et al. 2018	10	−58.30	9.40	10	−57.10	10.20	−0.12	[−0.99; 0.76]	0.7%
Mehren et al. 2019 HIIT	31	70.00	16.70	31	72.00	22.27	−0.10	[−0.60; 0.40]	1.5%
Crush & Loprinzi 2017 30min & 15min delay	22	1187.00	399.00	22	1226.00	381.00	−0.10	[−0.69; 0.49]	1.2%
Crush & Loprinzi 2017 20min & 15min delay	22	1131.00	312.00	22	1160.00	327.00	−0.09	[−0.68; 0.50]	1.2%
Crush & Loprinzi 2017 10min & 15min delay	22	1069.00	431.00	22	1099.00	315.00	−0.08	[−0.67; 0.51]	1.3%
Crush & Loprinzi 2017 45min & 5min delay	22	1201.00	418.00	22	1225.00	402.00	−0.06	[−0.65; 0.53]	1.3%
Abe et al. 2018 non−comparable_CG	13	181.80	79.40	26	186.20	97.80	−0.05	[−0.71; 0.62]	1.1%
Stroth et al. 2009	33	310.90	38.13	17	311.44	36.37	−0.01	[−0.60; 0.57]	1.3%
Brown & Bray, 2018* vigorous	44	561.27	76.36	5	561.85	85.08	−0.01	[−0.93; 0.92]	0.7%
Crush & Loprinzi 2017 20min & 5min delay	22	1097.00	360.00	22	1092.00	239.00	0.02	[−0.57; 0.61]	1.3%
Oberste et al. 2016* light	30	84.03	50.70	10	82.90	74.17	0.02	[−0.70; 0.74]	1.0%
Crush & Loprinzi 2017 45min & 15min delay	22	1076.00	480.00	22	1068.00	258.00	0.02	[−0.57; 0.61]	1.3%
Crush & Loprinzi 2017 30min & 5min delay	22	1210.00	454.00	22	1198.00	493.00	0.02	[−0.57; 0.62]	1.3%
Gothe et al. 2013 comparable CG	15	421.41	53.28	30	419.03	60.46	0.04	[−0.58; 0.66]	1.2%
de Marco et al. 2014	47	65.15	73.20	47	61.98	75.91	0.04	[−0.36; 0.45]	1.8%
Brown & Bray, 2018* light	22	565.43	79.10	5	561.85	85.08	0.04	[−0.93; 1.01]	0.6%
Kamijo et al. 2009 young_adults light	12	398.00	12.20	6	397.40	13.50	0.05	[−0.93; 1.03]	0.6%
Hogan et al. 2013	30	309.26	38.89	30	307.06	32.23	0.06	[−0.45; 0.57]	1.5%
van Regensburg & Taylor 2008	23	671.20	240.10	23	656.30	151.70	0.07	[−0.51; 0.65]	1.3%
Brown & Bray, 2018* HIIT	44	569.16	54.56	5	561.85	85.08	0.12	[−0.80; 1.05]	0.7%
Weng et al. 2015	25	510.43	59.30	25	503.43	47.00	0.13	[−0.43; 0.68]	1.3%
Hwang et al. 2016	29	−0.68	0.07	29	−0.69	0.08	0.13	[−0.38; 0.65]	1.5%
Gothe et al. 2013 non−comparable_CG	15	421.41	53.28	30	413.19	53.87	0.15	[−0.47; 0.77]	1.2%
Ligeza et al. 2018 HIIT	18	52.00	20.00	9	48.00	27.00	0.17	[−0.63; 0.97]	0.8%
Egger et al. 2018	50	154.13	153.53	54	121.30	138.67	0.22	[−0.16; 0.61]	1.9%
Oberste et al. 2016* vigorous	30	95.57	46.67	10	82.90	74.17	0.23	[−0.49; 0.95]	1.0%
Abe et al. 2018 comparable_CG	13	181.80	79.40	26	157.10	105.00	0.25	[−0.42; 0.92]	1.1%
Crush & Loprinzi 2017 60min & 15min delay	22	1166.00	412.00	22	1071.00	232.00	0.28	[−0.32; 0.87]	1.2%
Oberste et al. 2016* moderate	30	109.30	75.44	10	82.90	74.17	0.34	[−0.38; 1.06]	1.0%
Kamijo et al. 2009 older_adults light	12	480.90	20.40	6	473.80	16.70	0.35	[−0.64; 1.34]	0.6%
Random effects model	1966			1539			−0.27	[−0.37; −0.18]	83.2%
Heterogeneity: $I^2 = 41\%$, $\tau^2 = 0.0611$, $p < 0.01$									
**Random effects model**	**2345**			**1847**			**−0.27**	**[−0.35; −0.18]**	**100.0%**
Heterogeneity: $I^2 = 40\%$, $\tau^2 = 0.0591$, $p < 0.01$									

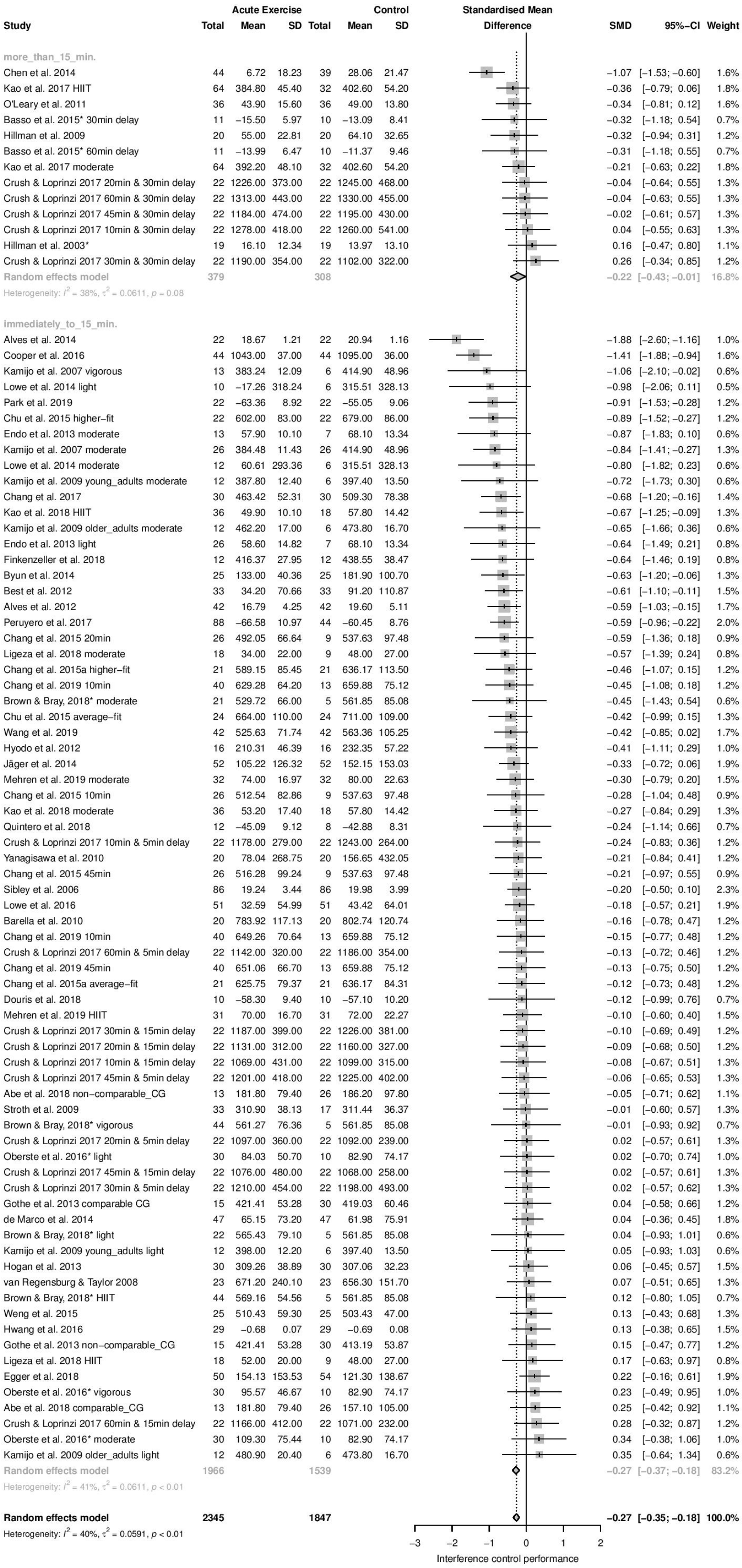

Figure 16 Subgroup analysis for delay between exercise cessation and interference control performance testing (time-dependent measures of interference control)

Study	Acute Exercise Total	Acute Exercise Mean	Acute Exercise SD	Control Total	Control Mean	Control SD	SMD	95%-CI	Weight
immediately to 15 min.									
Finkenzeller et al., 2018	12	−4.83	2.89	12	−3.08	2.19	−0.66	[−1.48; 0.17]	1.3%
Gothe et al. 2013 comparable CG	15	83.37	12.27	30	90.27	10.63	−0.61	[−1.24; 0.03]	2.2%
Oberste et al., 2016* moderate	30	−1.22	2.77	10	−0.45	1.89	−0.29	[−1.01; 0.43]	1.7%
Byun et al. 2014	25	93.60	6.50	25	95.60	8.00	−0.27	[−0.83; 0.29]	2.8%
Kamijo et al., 2009 young_adults moderate	12	94.90	7.97	6	96.30	2.77	−0.20	[−1.18; 0.79]	0.9%
Kamijo et al., 2009 young_adults light	12	95.20	6.24	6	96.30	2.77	−0.19	[−1.18; 0.79]	0.9%
Hogan et al., 2013	30	−22.39	6.39	30	−21.45	5.16	−0.16	[−0.67; 0.35]	3.4%
Brown & Bray, 2018* vigorous	22	91.79	7.35	5	92.92	3.87	−0.16	[−1.13; 0.81]	0.9%
Kamijo et al., 2007 moderate	24	−17.45	7.00	6	−16.42	7.23	−0.14	[−1.04; 0.75]	1.1%
Chang et al. 2015 45min	26	85.61	10.32	9	86.79	9.12	−0.11	[−0.87; 0.64]	1.5%
Ligeza et al., 2018 HIIT	18	−10.00	11.50	18	−9.00	9.00	−0.09	[−0.75; 0.56]	2.0%
Gothe et al. 2013 non−comparable_CG	15	83.37	12.27	30	84.37	12.61	−0.08	[−0.70; 0.54]	2.3%
Kamijo et al., 2009 older_adults moderate	12	94.70	4.50	6	94.80	4.16	−0.02	[−1.00; 0.96]	0.9%
Oberste et al., 2016* vigorous	30	−0.49	1.99	10	−0.45	1.89	−0.02	[−0.74; 0.70]	1.7%
Drolette et al., 2012	36	94.40	0.80	36	94.40	1.20	0.00	[−0.46; 0.46]	4.1%
Kamijo et al., 2007 vigorous	12	−16.34	6.28	6	−16.42	7.23	0.01	[−0.97; 0.99]	0.9%
Kao et al., 2018 HIIT	36	−5.91	5.34	18	−5.99	6.66	0.01	[−0.55; 0.58]	2.7%
Kao et al., 2018 moderate	36	−5.47	6.23	18	−5.99	6.66	0.08	[−0.49; 0.65]	2.7%
Brown & Bray, 2018* moderate	21	93.52	6.17	5	92.92	3.87	0.10	[−0.88; 1.08]	0.9%
Oberste et al., 2016* light	30	−0.26	1.80	10	−0.45	1.89	0.10	[−0.61; 0.82]	1.7%
Chang et al., 2019 10/20 min	40	94.05	7.37	13	93.10	7.70	0.13	[−0.50; 0.75]	2.2%
Cooper et al., 2016	44	94.10	10.30	44	93.00	6.40	0.13	[−0.29; 0.55]	4.9%
Chang et al., 2019 10/20 min	40	94.29	8.63	13	93.10	7.70	0.14	[−0.49; 0.77]	2.2%
Brown & Bray, 2018* HIIT	22	93.94	5.60	5	92.92	3.87	0.18	[−0.79; 1.16]	0.9%
Hyodo et al., 2012	16	−4.81	6.22	16	−6.10	5.92	0.21	[−0.49; 0.90]	1.8%
Chu et al., 2015 higher−fit	22	84.00	21.00	22	80.00	15.00	0.22	[−0.38; 0.81]	2.5%
Weng et al., 2015	25	93.00	5.11	25	91.60	6.70	0.23	[−0.33; 0.79]	2.8%
Chang et al., 2019 45 min	40	94.71	6.53	13	93.10	7.70	0.23	[−0.39; 0.86]	2.2%
Alves et al., 2012	42	−0.61	1.03	42	−1.01	2.07	0.24	[−0.19; 0.67]	4.7%
Brown & Bray, 2018* light	22	94.32	5.78	5	92.92	3.87	0.25	[−0.73; 1.22]	0.9%
Lowe et al., 2016	51	98.60	1.30	51	96.60	9.60	0.29	[−0.10; 0.68]	5.7%
Kamijo et al., 2009 older_adults light	12	96.10	3.81	6	94.80	4.16	0.32	[−0.67; 1.30]	0.9%
Chang et al. 2015 10min	26	89.13	6.31	9	86.79	9.12	0.32	[−0.44; 1.08]	1.5%
Ligeza et al., 2018 moderate	18	−6.00	5.00	18	−9.00	9.00	0.40	[−0.26; 1.06]	2.0%
Yanagisawa et al., 2010	20	−7.00	1.50	20	−8.00	1.40	0.68	[0.04; 1.31]	2.1%
Chang et al. 2015 20min	26	92.60	6.46	9	86.79	9.12	0.79	[0.01; 1.57]	1.4%
Chu et al., 2015 lower−fit	24	93.00	6.00	24	86.00	9.00	0.90	[0.30; 1.50]	2.4%
Random effects model	944			631			0.10	[0.00; 0.21]	77.5%
Heterogeneity: $I^2 = 0\%$, $\tau^2 = 0$, $p = 0.71$									
more than 15 min.									
Hillman et al., 2003*	19	91.55	6.97	19	92.97	4.68	−0.23	[−0.87; 0.40]	2.1%
Kao et al., 2017 moderate	64	91.30	6.90	32	91.10	7.20	0.03	[−0.40; 0.45]	4.8%
O'Leary et al., 2011	36	89.60	7.60	36	88.10	8.40	0.19	[−0.28; 0.65]	4.0%
Chen et al., 2014	44	93.86	0.25	39	93.77	0.36	0.29	[−0.14; 0.72]	4.6%
Kao et al., 2017 HIIT	64	93.20	5.80	32	91.10	7.20	0.33	[−0.10; 0.76]	4.7%
Hillman et al., 2009	20	−6.60	4.47	20	−10.30	8.50	0.53	[−0.10; 1.17]	2.2%
Random effects model	247			178			0.20	[0.00; 0.39]	22.5%
Heterogeneity: $I^2 = 0\%$, $\tau^2 = 0$, $p = 0.55$									
**Random effects model**	**1191**			**809**			**0.13**	**[0.03; 0.22]**	**100.0%**
Heterogeneity: $I^2 = 0\%$, $\tau^2 = 0$, $p = 0.74$									

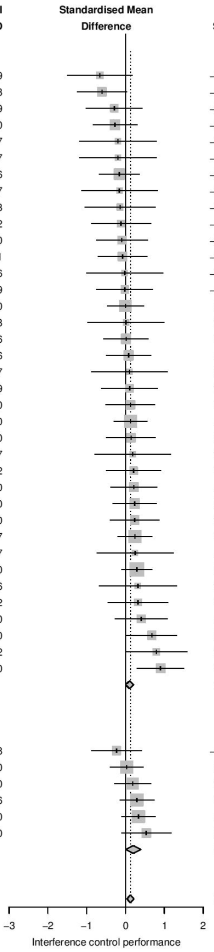

Figure 17 Subgroup analysis for delay between exercise cessation and interference control performance testing (accuracy measures of interference control)
